# SMYD3 drives the proliferation in gastric cancer cells via reducing EMP1 expression in an H4K20me3-dependent manner

**DOI:** 10.1038/s41419-023-05907-9

**Published:** 2023-06-29

**Authors:** Yi Zeng, Gang Ma, Fenglin Cai, Pengliang Wang, Han Liang, Rupeng Zhang, Jingyu Deng, Yong Liu

**Affiliations:** 1 Department of Gastric Surgery, Tianjin Medical University Cancer Institute & Hospital, National Clinical Research Center for Cancer, Key Laboratory of Cancer Prevention and Therapy, Tianjin, Tianjin’s Clinical Research Center for Cancer, Tianjin, 300060 PR China; 2 Department of Gastrointestinal Surgical Oncology, Clinical Oncology School of Fujian Medical University, Fujian Cancer Hospital, Fuzhou, 350000 Fujian PR China; 3 Department of Biochemistry and Molecular Biology, The Province and Ministry Co-sponsored Collaborative Innovation Center for Medical Epigenetics, School of Basic Medical Sciences, Tianjin Medical University, Tianjin, 300060 PR China; 4 Department of Gastrointestinal Surgery, Sun Yat-sen Memorial Hospital, Sun Yat-sen University, Guangzhou, 510120 PR China

**Keywords:** Gastric cancer, Cancer epigenetics

## Abstract

Protein lysine methyltransferase SET and MYND domain-containing 3 (SMYD3) is aberrantly expressed in various cancer settings. The mechanisms that SMYD3 activates the expression of critical pro-tumoral genes in an H3K4me3-dependent manner have been well described in previous reports. Besides H3K4me3, H4K20me3 is another catalytic product of SMYD3, however it is a transcriptionally repressive hallmark. Since it is not clear that how SMYD3-elicited transcriptionally repressive program functions in cancer, we used gastric cancer (GC) as a model to investigate the roles of SMYD3-H4K20me3. Herein, online bioinformatics tools, quantitative PCR, western blotting and immunohistochemistry assays demonstrated that SMYD3 expression was markedly increased in GC tissues from our institutional and The Cancer Genome Atlas (TCGA) cohort. Additionally, aberrantly increased SMYD3 expression was closely associated with aggressive clinical characteristics and poor prognosis. Depletion of endogenous SMYD3 expression using shRNAs significantly attenuates the proliferation in GC cells and Akt signaling pathway in vitro and in vivo. Mechanistically, chromatin immunoprecipitation (ChIP) assay showed that SMYD3 epigenetically repressed the expression of epithelial membrane protein 1 (EMP1) in an H4K20me3-dependent manner. Gain-of-function and rescue experiments validated that EMP1 inhibited the propagation of GC cells and reduced p-Akt (S473) level. Based on these data, pharmaceutical inhibition of SMYD3 activity using the small inhibitor BCI-121 deactivated Akt signaling pathway in GC cells and further impaired the cellular viability in vitro and in vivo. Together, these results demonstrate that SMYD3 promotes the proliferation in GC cells and may be a valid target for therapeutic intervention of patients with GC.

## Introduction

Gastric cancer (GC) remains a heavy global health problem since it is currently the fifth commonly diagnosed malignancy and the fourth leading cause of cancer-related deaths in the world [1]. However, few effective therapeutical options exist, especially for patients with advanced GC. Beside the standard chemotherapy drugs (fluoropyrimidines, platinums, taxanes and irinotecan), less than 20% of patients with GC have HER2 protein overexpression and could benefit from trastuzumab [2]. Angiogenic signaling pathway is another target of treating GC, as ramucirumab or bevacizumab could improve survival of patients who have progressed from the first-line chemotherapy [2]. As for patients who become refractory to the first- and second-line chemotherapy, immune checkpoint blockade agents came to the forefront, but the efficacy is largely determined by patients’ microsatellite instability (MSI) status and PD-1/PD-L1 expression [3]. Basic and translational research in the past decades not only identified critical genes contributing to the initiation and progression of GC but they also uncovered the complexity of this heterogeneous disease. These discoveries are paramount in identifying novel targets and spurring more effective drugs. High-throughput technologies have revealed significant epigenetic aberrations, and evidence of epigenetic abnormalities to promote GC development in supporting the idea that aberrant epigenetic alterations could be promising targets in GC treatment is accumulating [4, 5]. However, few epigenetic factors are now established as a treatment for GC, thus deeper functional understanding of how these factors affect aggressive phenotypes of GC cells is significant.

As the components of the nucleosome, histones are subjected to the diverse posttranslational modifications (PTMs), which are essential for maintaining the proper structure and function of chromatin [6]. The methyl groups are commonly added to the specific lysine residues within histones, especially H3 and H4, by methyltransferases (KMTs) and are removed by demethylases (KDMs). Thus, it is plausible that disruption of these key enzymes could promote tumorigenesis [7, 8]. The inhibitors targeting histone methyltransferase factors have been explored for cancer therapies and have undergone clinical trials [9–11]. The Su(Var)3-9, Enhancer-of-zeste and Trithorax (SET) and Myeloid, Nervy, and DEAF-1 (MYND) domain-containing (SMYD) protein family have five members in mice and humans, namely SMYD1, SMYD2, SMYD3, SMYD4 and SMYD5. The pro-tumoral functions of SMYD3 have been well investigated in different cancer settings [12, 13]. One major mechanism that SMYD3 displays is that this methyltransferase interacts with one of its catalytic products H3K4me3, as an epigenetic hallmark of active transcription, to potentiate the transcription of several key genes favoring survival and proliferation of cancer cells [13]. One seminal study in this field was reported that bind of SMYD3 to H3K4Me3-modified histone tails facilitated this enzyme to assemble in the core promoter regions of active transcriptional genes, further markedly increasing transcription of a set of oncogenes in hepatocellular and colorectal cancer [14]. On the other hand, H4K20me3, another methylated histone product of SMYD3, is usually enriched in heterochromatin and dormant genes [15–17]. Notably, H4K20 methylation plays important roles in regulating genomic integrity, which is an essential process in cancer biology [18, 19]. However, the functions and the underlying mechanisms of H4K20me3 in cancer remain largely unknown.

This study has demonstrated that SMYD3 exhibits high expression levels in GC tissues, which is indicative of an unfavorable prognosis in GC patients. The attenuation of proliferation and deactivation of Akt signaling in GC cells was observed upon SMYD3 knockdown. Furthermore, the study has revealed that SMYD3 deficiency leads to a reduction in H4K20me3 expression in the promoter of epithelial membrane protein 1 (EMP1), resulting in the re-expression of EMP1. The effect of SMYD3 deficiency on the proliferation and Akt signaling in GC cells was largely recapitulated by EMP1. Finally, pharmacological inhibition of SMYD3 activity further suggested that SMYD3 may be a valid target for treatment of patients with GC.

## Results

### SMYD3 is increased in GC tissues and indicates patients’ poor prognosis

**Fig. 1:**
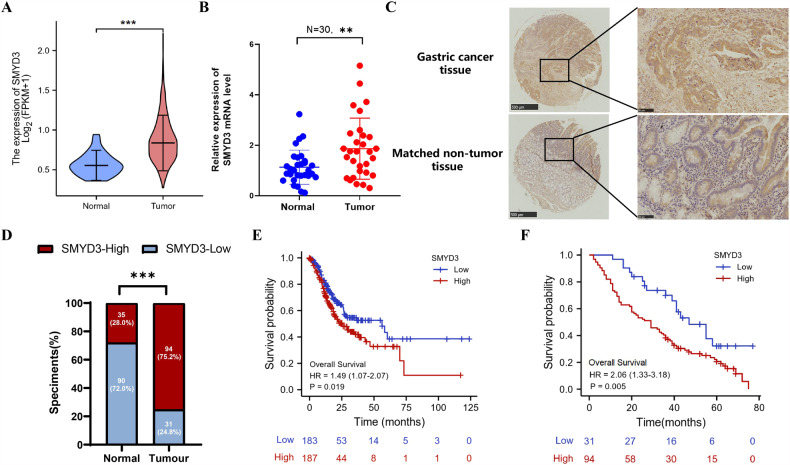
Increased expression of *SMYD3* in GC tissue is indicative of poor prognosis. **A** SMYD3 mRNA level is significantly upregulated in GC tissues (*n* = 375) compared with normal tissues (*n* = 32) from TCGA GC database (*p* < 0.001). **B** SMYD3 mRNA expression is markedly increased in in-house GC tissues (*n* = 30) by qPCR. **C** SMYD3 staining in GC tissues is stronger than that in normal tissues. Representative IHC images are shown here. A total of 125 pairs of tumor and normal tissues were analyzed. **D** SMYD3 staining was scored (0–12) and SMYD3 protein level was remarkably increased in GC samples relative to normal ones (*p* < 0.001). **E** Higher SMYD3 expression is indicative of poorer overall survival rate from TCGA GC database (*p* = 0.019). **F** Higher SMYD3 expression is indicative of poorer overall survival rate in institutional patients (*p* = 0.005).

In order to elucidate SMYD3’s role in GC, we first examined SMYD3 expression in GC tissues. A significant increase in SMYD3 mRNA levels was found in GC tissues (*N* = 375) when compared with normal tissue (*N* = 32) in the GC cohort from The Cancer Genome Atlas (TCGA) (*p* < 0.001) (Fig. 1A). We found that SMYD3 mRNA expression was significantly higher in our institutional GC tissues (*N* = 30, *p* < 0.01) than it was in the normal tissues matched for GC (Fig. 1B). We performed an immunohistochemistry (IHC) assay on 125 pairs of in-house GC and matched noncancerous tissues, showing that SMYD3 staining in the cell cytoplasm and nucleus was positive (Fig. 1C). Based on SMYD3 staining intensity (0–12), GC samples displayed substantially stronger staining than normal ones (*p* < 0.001) (Fig. 1D). Furthermore, patients with higher SMYD3 expression had larger tumor sizes and a more advanced pT stage (Table 1). As demonstrated in earlier studies [20, 21], higher SMYD3 expression was associated with a worse prognosis in several types of cancer, including hepatocellular carcinoma and claudin-low breast cancer. In consistent with these findings, we also found that patients with lower SMYD3 expression had better overall survival (OS) than those with higher SMYD3 expression in the TCGA cohort (Fig. 1E). In our institutional patients with GC, increased SMYD3 expression was associated with poor prognoses (Fig. 1F). As a result of multivariate regression analysis, SMYD3 was found to be an independent prognostic factor for OS (*p* = 0.005) (Table 2).

**Table 1:** Patient characteristics.

Characteristic	Low expression of SMYD3 (*n* = 31)	High expression of SMYD3 (*n* = 94)	*p*
Gender, *N* (%)			0.795
Female	10 (32.3%)	28 (29.8%)	
Male	21(67.7%)	66 (70.2%)	
Age, *N* (%)			0.407
≤65	22 (71.0%)	59 (55.6%)	
>65	9 (29.0%)	35 (44.4%)	
pT stage, *N* (%)			0.048
T2	4 (12.9%)	3 (3.2%)	
T3	4 (12.9%)	4 (4.3%)	
T4a	21 (67.7%)	75 (79.8%)	
T4b	2 (6.5)	12 (12.7%)	
pN stage, *N* (%)			0.604
N0	20 (21.3%)	10 (32.3%)	
N1	9 (9.6%)	4 (12.9%)	
N2	23 (24.5%)	7 (22.6%)	
N3a	22 (23.4%)	4 (12.9%)	
N3b	20 (21.3%)	6 (19.4)	
Lauren classification, *N* (%)			0.135
Intestinal	5 (16.1%)	28 (29.8%)	
Diffuse	26 (83.9%)	66 (70.2%)	
Soft tissue invasion			
No	24 (77.4%)	66 (70.2%)	0.438
Yes	7 (22.6%)	28 (29.8%)	
Tumor size			
<4 cm	8 (25.8%)	10 (10.6%)	0.037
≥4 cm	23 (74.2%)	84 (89.4%)	
Tumor location, *N* (%)			0.921
Upper third	4 (12.9%)	15 (16.0%)	
Middle third	3 (9.7%)	11 (11.7%)	
Lower third	17 (54.8%)	45 (47.9%)	
>2/3 stomach	7 (22.6%)	23 (24.4%)

**Table 2:** Univariate and multivariate analysis.

Variable	Cases	Five-year OS rate (%)	Univariate *p* value^a^	Hazard ratio (95% CI)	Multivariate *p* value^b^
Gender			0.041	1.627 (1.029–2.573)	0.037
Female	38	15.9			
Male	87	27.5			
Age (years)			0.052	1.476 (0.937–2.325)	0.093
≤60	81	28.9			
>60	44	13.7			
Tumor size (cm)			0.055	1.576 (0.801–3.101)	0.188
<4	18	23.9			
≥4	107	23.6			
Tumor location			0.500	1.023 (0.836–1.253)	0.823
Upper third	19	25.1			
Middle third	14	14.3			
Lower third	62	26.5			
>2/3 stomach	30	24.1			
Soft tissue invasion			0.724	0.627 (0.383–1.025)	0.063
Yes	35	22.9			
No	90	24.1			
Lauren classification			0.476	1.026 (0.597–1.763)	0.927
Intestinal	464	33.4			
Diffuse	410	21.0			
pT stage			0.204	1.044 (0.699–1.560)	0.834
T2	7	33.3			
T3	8	20.8			
T4a	96	25.1			
T4b	14	9.5			
pN stage			0.002	1.342 (1.140–1.579)	<0.001
N0	30	41.9			
N1	13	26.6			
N2	30	12.1			
N3a	26	18.1			
N3b	26	12.4			
SMYD3 expression^c^			0.005	1.803 (1.035–3.140)	0.037
Low	31	20.7			
High	94	32.2			

Values in parentheses are 95 percent confidence intervals.

^a^Log-rank test.

^b^Cox proportional hazards model.

^c^Determined by immunohistochemical staining.

### SMYD3 silencing attenuates the proliferation in GC cells

**Fig. 2:**
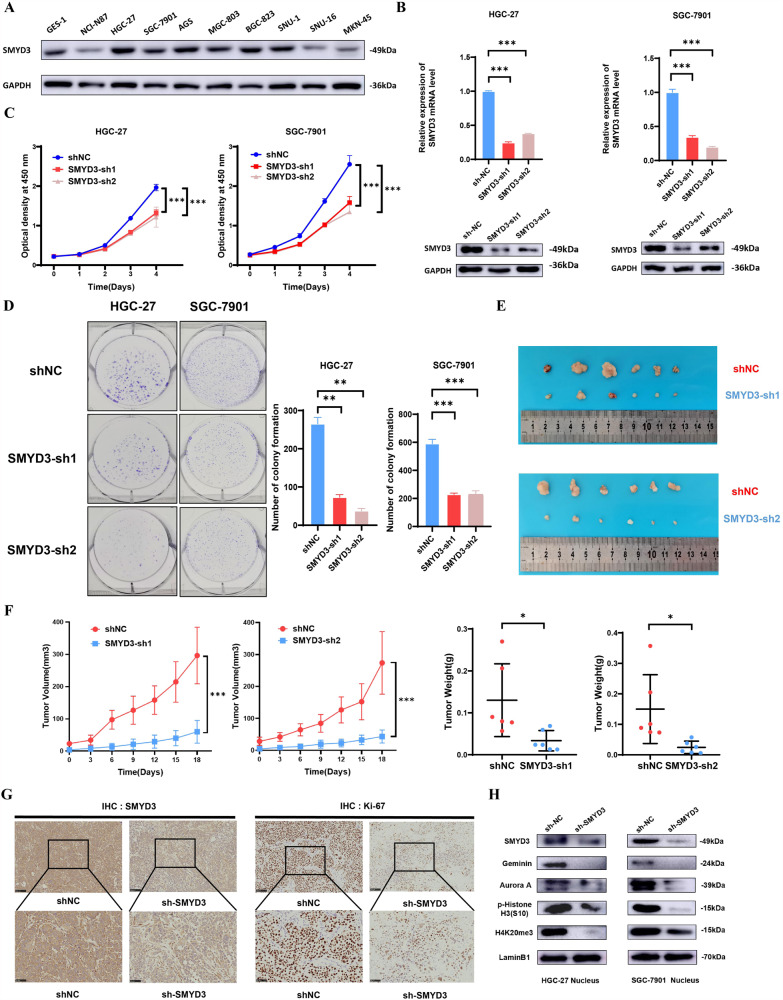
SMYD3 silencing inhibits the proliferation in GC cells. **A** SMYD3 expression in the immortalized human gastric epithelial cell line GES-1 and a set of GC cell lines were examined by western blotting assay. **B** The endogenous SMYD3 expression in HGC-27 and SGC-7901 cells was depleted using shRNAs verified by qPCR assay and western blotting assay. **C** SMYD3 depletion markedly inhibits the propagation in HGC-27 and SGC-7901 cells as shown by CCK8 assay. **D** SMYD3 depletion markedly suppresses the colony formation in HGC-27 and SGC-7901 cells. **E** SMYD3 depletion inhibited growth of tumors in vivo. **F** Tumor growth curves and tumor weight shows the suppressive effect of SMYD3 depletion in vivo. **G** IHC shows that Ki-67 staining was markedly weaker in tumor masses originated from SMYD3-depleted SGC-7901 cells. **H** The nuclear level of Geminin, Aurora A, p-Histone H3 (S10) and H4K20me3 dramatically decreased in HGC-27/SGC-7901 cells with SMYD3 knockdown.

Our study assessed the role of SMYD3 in GC cell proliferation given that increased SMYD3 expression was associated with enlarged tumor sizes and advanced pT stage. Before performing the functional investigations, we found that SMYD3 protein level was remarkably increased in nine GC cell lines compared to those in GES-1, an immortalized human gastric epithelial cell line (Fig. 2A). Then we used shRNAs to deplete the endogenous SMYD3 expression in HGC-27 and SGC-7901 cells (Fig. 2B). CCK-8 and colony formation assay demonstrated that SMYD3 depletion significantly inhibited HGC-27 and SGC-7901 cell growth within 4 days and reduced the number of colonies, in comparison to control cells (Fig. 2C, D). Then, the control and the SMYD3-knockdown SGC-7901 cells were injected subcutaneously into immunocompromised mice to test the growth of GC cells in vivo. As a result of reduced SMYD3 expression, SGC-7901 cells grew at a significantly decreased rate, and the weights of the harvested tumor masses were markedly reduced (Fig. 2E, F). Moreover, IHC confirmed that SMYD3-depleted SGC-7901 tumor masses manifested significantly weaker Ki-67 staining than the tumor masses derived from the control cells (Fig. 2G). The cytoplasmic and nuclear fractions were separated, and we detected a possible effect of SMYD3 silencing on cells' proliferation proteins, finding that the nuclear level of Geminin, Aurora A and p-Histone H3 (S10) dramatically decreased in HGC-27 and SGC-7901 cells with SMYD3 knockdown (Fig. 2H). Together, these results indicated that SMYD3 stimulated the proliferation in GC cells.

### SMYD3 inhibits EMP1 expression in an H4K20me3-dependent manner

**Fig. 3:**
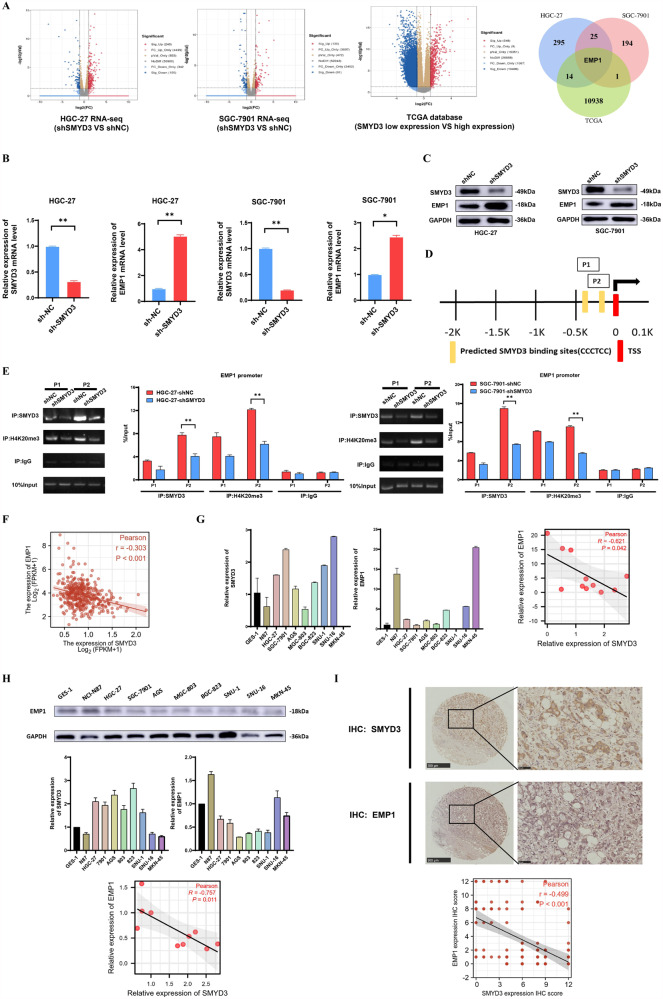
SMYD3 dictates EMP1 expression in an H4K20me3-dependent manner. **A** The differentially expressed genes (DEGs) from RNA-seq data (GSE214155) were presented in the volcanic maps, with 345 genes altered in HGC-27 and 221 genes in SGC-7901 cells (|log2(FC)| > 0.5 and *p* < 0.05). The GC cohort from TCGA was stratified into the low (0–50%) and the high (50–100%) SMYD3-expression group, and the DEGs, with 10,954 altered between the two groups (|log2(FC)| > 0.5 and *p* < 0.05). Among the DEGs overlapped in the three datasets, EMP1 was the highest confidence target for SMYD3-H4K20me3. **B** Knockdown of endogenous SMYD3 expression elicited significantly increased EMP1 mRNA expression in HGC-27 and SGC-7901 cells by qPCR assay. **C** Knockdown of endogenous SMYD3 expression elicited significantly increased EMP1 protein expression in HGC-27 and SGC-7901 cells by immunoblot assay. **D** The specific SMYD3 DNA binding site (–CCCTCC-) P1/P2 in the EMP1 promoter. **E** ChIP assay revealed that SMYD3 and H4K20me3 were significantly enriched on the binding site P2. **F** EMP1 expression reversely correlated with SMYD3 expression in GC tissues (*r* = −0.303, *p* < 0.001) in TCGA GC cohort. **G** SMYD3 mRNA level was reversely correlated with EMP1 expression in GES-1 cells and a panel of GC cell lines (*r* = −0.621, *p* = 0.042) by qPCR assay. **H** SMYD3 protein level was reversely correlated with EMP1 expression in GES-1 cells and a panel of GC cell lines (*r* = −0.621, *p* = 0.042) by immunoblot assay. **I** EMP1 protein level also correlated reversely with SMYD3 protein level (*r* = −0.757, *p* = 0.011) via IHC in the same in-house GC specimens (*n* = 125).

SMYD3 has been identified as being responsible for producing H4K20me3 in previous studies [15–17]. Meanwhile, we found that SMYD3-deficient cells manifested a marked reduction in H4K20me3 levels (Fig. 2H). We profiled the gene expression pattern between the control (shNC) and the SMYD3-deficient (shSMYD3) HGC-27 or SGC-7901 cells, respectively. Knockdown of SMYD3 led to 345 differentially expressed genes (DEGs) in HGC-27 cells and 221 DEGs in SGC-7901 cells, which were displayed in the volcanic maps (Fig. 3A). In addition, GC tissues from TCGA gastric cohort was stratified into the low SMYD3 expression (0–50%) and the high SMYD3 expression (50–100%) group, and DEGs between these two groups were compared accordingly, finding that 10,954 genes were significantly altered (Fig. 3A). As shown in the Venn diagram in Fig. 3A, EMP1 was the gene in common among the three groups of DEGs. Thus, we set out to examine the function and the mechanism of EMP1 in GC cells.

qPCR and immunoblot assay confirmed that knockdown of endogenous SMYD3 expression elicited significantly increased EMP1 expression in HGC-27 and SGC-7901 cells (Fig. 3B, C). As H4K20me3 is an epigenetic hallmark of inactive transcription, we speculated that EMP1 transcription was directly regulated by SMYD3-H4K20me3. Previous reports identified that the binding site sequence of SMYD3 was 5’-CCCTCC-3’ [22, 23], thus we analyzed the sequence covering 2 kb upstream the transcription start site (TSS) and found two potential binding sites (P1/P2) (Fig. 3D). ChIP assay revealed that SMYD3 and H4K20me3 were significantly enriched on the binding site P2 (Fig. 3E). In addition, EMP1 expression reversely correlated with SMYD3 expression in GC tissues (*N* = 375) from TCGA GC cohort (*r* = −0.303, *p* < 0.001) (Fig. 3F). A panel of GC cell lines and GES-1 cells also showed a reverse correlation between SMYD3 and EMP1 expression at both mRNA (*r* = −0.621, *p* = 0.042) and protein level (*r* = −0.757, *p* = 0.011) (Fig. 3G, H). EMP1 protein expression was then detected via IHC in the same in-house GC specimens (*N* = 125), showing that it also correlated reversely with SMYD3 (*r* = −0.499, *p* < 0.001) (Fig. 3I). These results collectively demonstrated that SMYD3-H4K20me3 directly repressed the transcription of *EMP1* in GC cells.

### SMYD3 deficiency and increased EMP1 mitigate Akt signaling in GC cells

**Fig. 4:**
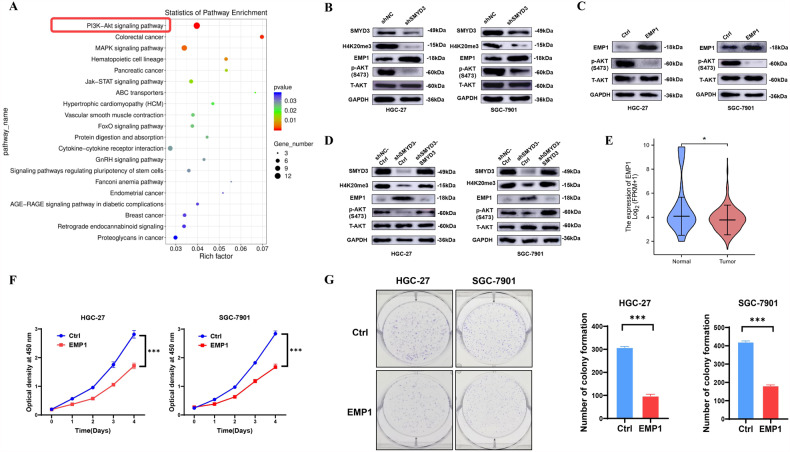
SMYD3 deficiency and increased EMP1 mitigate Akt signaling in GC cells. **A** KEGG pathway enrichment analysis of RNA-seq data (GSE214155) found that PI3K-Akt was the most influenced signaling pathway. **B** Immunoblotting analysis for H4K20me3 level, as well as EMP1, p-AKT(S473) and T-AKT in HGC-27 and SGC-7901 cells with SMYD3 deficiency. **C** Immunoblotting analysis for p-AKT(S473) level, as well as T-AKT in HGC-27 and SGC-7901 cells with exogenous expression of EMP1. **D** Immunoblotting analysis for H4K20me3 level, as well as EMP1, p-AKT(S473) and T-AKT in HGC-27 and SGC-7901 cells with SMYD3 deficiency combined with re-expression of SMYD3. **E** EMP1 expression was decreased in GC tissues (*N* = 375) relative to normal ones (*N* = 32) (*p* < 0.05) in TCGA GC cohort. **F** EMP1 inhibits the cell growth in HGC-27 and SGC-7901 cells by CCK-8 assay. **G** EMP1 suppresses the colony formation in HGC-27 and SGC-7901 cells.

We conducted Kyoto Encyclopedia of Genes and Genomes (KEGG) pathway enrichment analysis based on the RNA-seq data (GSE214155) and found that PI3K-Akt was the most affected signaling pathway by SMYD3 deficiency (Fig. 4A). Immunoblot assay confirmed that p-Akt (S473) level was markedly decreased in GC cells with SMYD3 deficiency or exogenous expression of EMP1 (Fig. 4B, C). Re-expression of exogenous SMYD3 restored p-Akt (S473) level while reduced EMP1 expression in GC cells with depletion of endogenous SMYD3 (Fig. 4D).

To explore the role of EMP1 in GC, we first examined its expression in GC tissues, finding that it was decreased in GC tissues (*N* = 32) relative to normal ones (*N* = 375) from TCGA cohort (*p* < 0.05) (Fig. 4E). Functional investigations demonstrated that HGC-27 and SGC-7901 cells with exogenous EMP1 expression manifested delayed growth and impaired capacity of colony formation, when compared with control cells (Fig. 4F, G). Together, these data suggested that overexpressed EMP1 inhibited cellular propagation via deactivating PI3K-Akt signaling pathway, the phenotype and the mechanism which were reminiscent of deficient SMYD3 in GC cells.

### The pro-proliferation effect of SMYD3 is dependent on the reduced EMP1 expression in GC cells

**Fig. 5:**
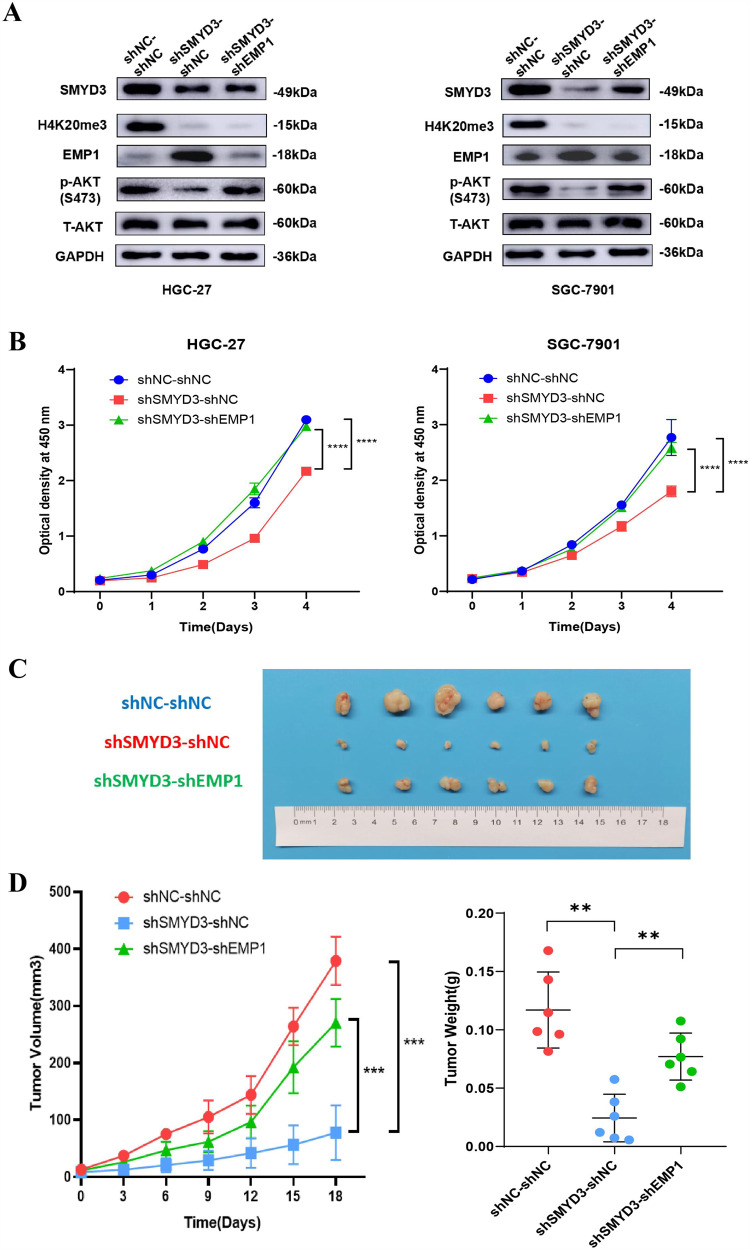
The pro-proliferative effect of SMYD3 is dependent on reducing EMP1 expression in GC cells. **A** Reduced EMP1 expression relieved the deactivation of SMYD3 knockdown on the level of p-Akt (S473) in both HGC-27 and SGC-7901 cells by immunoblot assay. **B** Decreased EMP1 expression reignited the proliferation of HGC-27 and SGC-7901 cells in vitro by CCK-8 assay which was suppressed by knockdown of SMYD3 alone. **C** Decreased EMP1 expression reignited the proliferation of HGC-27 and SGC-7901 cells in vivo. **D** Tumor growth curves and tumor weight shows that decreased EMP1 expression reignited the GC cells proliferation in vivo.

To test whether silenced EMP1 expression was required for the proliferation-promoting function of SMYD3, we reduced the endogenous expression of EMP1 via shRNA in the presence of control shRNA or that targeting SMYD3 (Fig. 5A). The level of p-Akt (S473) was also increased in the GC cells with depletion of both SMYD3 and EMP1 (Fig. 5A). Functional analysis showed that decreased EMP1 relieved the inhibition on the proliferation induced by knockdown of SMYD3 in HGC-27 and SGC-7901 cells in vitro and in vivo (Fig. 5B–D). Together, our results demonstrated that repression of EMP1 expression was indispensable, at least partially, for the hyperproliferative function of SMYD3 in GC cells.

### Pharmaceutical inhibition of SMYD3 inhibits the proliferation in GC cells

**Fig. 6:**
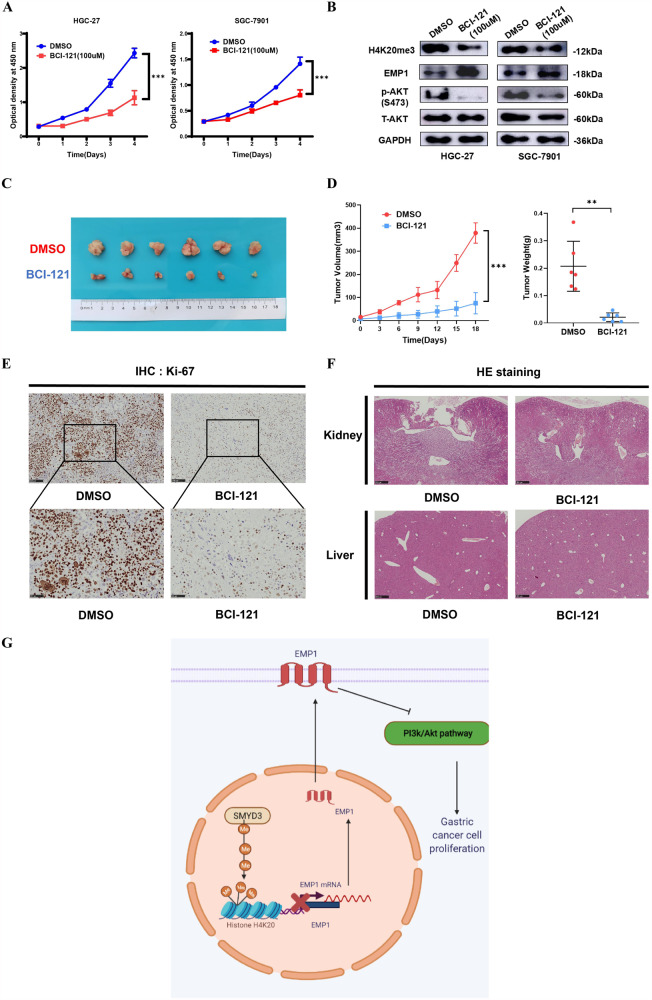
Pharmaceutical inhibition of SMYD3 inhibits the proliferation in GC cells. **A** SMYD3 inhibitor BCI-121 (100 μM) substantially mitigated the cellular proliferation of HGC-27 and SGC-7901 cells within the indicated time interval by CCK-8 assay. **B** BCI-121 remarkably reduced the level of H4K20me3 and p-Akt (S473), as well as increased EMP1 protein level in HGC-27 and SGC-7901 cells by immunoblot assay. **C** Intratumoral injection of BCI-121 (100 μM) markedly repressed the growth of SGC-7901 cells in vivo. **D** Tumor growth curves and tumor weight shows that BCI-121 markedly repressed the growth of SGC-7901 cells in vivo. **E** IHC shows that Ki-67 staining was markedly weaker in tumor masses originated from BCI-121-treated mice. **F** Administration of BCI-121 in vivo did not elicit evident damages to the livers and kidneys of the BCI-121-treated mice by HE staining assay. **G** Schematic diagram: SMYD3 promoted Akt signaling pathway and thus the proliferation via H4K20me3-mediated suppression of EMP1 expression in GC cells.

Since SMYD3 plays critical roles in several cancer types, a few specific inhibitors, such as BCI-121, were developed [24]. BCI-121 was then tested in GC cells. BCI-121 (100 μM) substantially mitigated the cellular proliferation of HGC-27 and SGC-7901 cells within the indicated time interval, as shown by CCK-8 assay (Fig. 6A). Meanwhile, BCI-121 decreased H4K20me3 and p-Akt (S473) levels, whereas increased the expression level of EMP1 protein in HGC-27 and SGC-7901 cells (Fig. 6B). More important was that intratumoral injection of BCI-121 (100 μM) markedly repressed the growth of SGC-7901 cells in vivo (Fig. 6C–E). BCI-121 administration in vivo did not elicit obvious liver or kidney damage in these treated mice (Fig. 6F). All these data corroborated that targeting SMYD3 using small molecule inhibitors slowed down the growth of GC cells, suggesting that SMYD3 could be a therapeutic target for patients with GC.

## Discussion

The aberrant overexpression of SMYD3 in multiple cancer types indicates its pro-tumor functions. For example, SMYD3 promoted the transcription of sphingosine-1-phosphate receptor 1 (S1PR1) to enhance growth and migration in hepatocellular carcinoma [21]. SMYD3 also facilitated implant metastasis of ovarian cancer cells via increasing the expression of ITGB6 and ITGAM [25]. In pancreatic ductal adenocarcinoma, S100A11 bound to SMYD3 and then activated the expression of transketolase (TKT), thus stimulating pentose phosphate pathway to aggregate the malignant phenotypes [26]. Another piece of evidence corroborating the implication of SMYD3 in cancer metabolism was that SMYD3 increased pyruvate kinase M2 (PKM2) expression to support a hyperproliferative phenotype in diffuse large B-cell lymphoma (DLBCL) [27]. We previously reported that SMYD3 was markedly increased in GC tissues and indicative of unfavorable prognosis [28]. Another group showed that SMYD3 upregulated the expression of ASCL2, which was a stem cell transcription factor, in GC cells [29]. The ASCL2-positive GC cell population displayed stronger capacity of self-renewal and tumorigenicity, thereby suggesting that SMYD3 was implicated in regulating stemness in GC cells [29]. In this study, we confirmed that SMYD3 promoted the propagation in GC cells, which was consistent with the previous findings obtained in other cancer settings. Unlike these investigations, we focused on the transcription repression program induced by SMYD3’s product H4K20me3, since the regulatory mechanism that SMYD3 activated the transcription of genes favoring cancerous traits in the H3K4me3-dependent manner have been well described before [14, 21, 25–27, 29].

H4K20me3, as an evolutionarily conserved modification, is primarily enriched in heterochromatin regions, such as telomeres, centromeres and repetitive DNA elements [18]. Like H4K20me1 and H4K20me2, H4K20me3 plays critical roles in DNA replication. H4K20me3 dictated replication initiation sites by maintaining the activity of some ORCA/LRWD1-related origins, thus preventing the delay in replication of heterochromatin regions [30]. Additionally, H4K20me3 is paramount to sustaining telomere integrity. Depletion of SUV4-20H1/2, the histone methyltransferases producing H4K20me3, reduced the overall level of H4K20me3 and subsequent elongation of telomeres [31]. More molecules implicated in this process have been identified recently. TERRAs, the long non-coding RNAs generated from telomeres, interacted with PRC2 to establish sites of H4K20me3 in telomeres [32]. Periodic tryptophan protein 1 (PWP1) deficiency decreased H4K20me3 expression and resulted in telomere shortening in mouse and human cells [33]. Thus, it is plausible that aberrant reduction in H4K20me3 level disturbs genome integrity, which in turn makes cells become more susceptible to malignant transformation. In the preneoplasia tissues of lungs, H4K20me3 level was markedly reduced and it continued to decrease as the disease progressed [34]. Increased telomere length attributable to loss of SUV4-20H/H4K20me3 promoted the potential of neoplasia in pluripotent stem cells [35]. Notably, since dysfunctional SMYD3 undoubtedly resulted in abnormal expression of H4K20me3 across genome, the expression of some genes that alleviate aggressive phenotypes could be silenced accordingly in cancer cells, which is probably an additional mechanism involved in the cancer-promoting functions of SMYD3. However, direct evidence supporting this paradigm is yet sparse. One report showed that SMYD3 depletion reduced H4K20me3 level to upregulate *CCND2* expression, whose restoration attenuated the hyperproliferative phenotype in prostate cancer LNCaP cells [17, 36]. In GC cells, we found that SMYD3 silencing decreased H4K20me3 level, and SMYD3/H4K20me3 were enriched in the upstream region of *EMP1* transcription start site. Restored EMP1 expression phenocopied the effect of SMYD3 deficiency on the Akt signaling pathway and the propagation in GC cells, thereby demonstrating that H4K20me3-mediated repression in the expression of some critical genes, such as *EMP1*, was a requisite for GC progression.

EMP1 belongs to the epithelial membrane protein family, and its functions vary depending on distinct cancer contexts. EMP1 contributed to gefitinib resistance in lung cancer as well as in head and neck cancer [37, 38], indicating that EMP1 could be a marker of therapeutic efficacy. One latest study showed that EMP1 marked a subpopulation of colorectal cancer cells with remarkably enhanced tumor relapse, and genetic elimination of this EMP1^high^ cell population halted metastatic recurrence [39]. Likewise, EMP1 was positively involved in invasion induced by constitutively activated EGFR signaling pathway [40]. On the other hand, EMP1 knockdown promoted metastasis by resisting oxidative stress and ferroptosis in bladder cancer cells [41]. In our model, we found that EMP1 inhibited the proliferation in GC cells, this result was in line with the previous report [42]. Moreover, we revealed that EMP1 mitigated Akt signaling pathway, however we did not clarify the mechanism by which EMP1 regulated the phosphorylation of Akt in this study. Pilot experiments in our lab recently showed that EMP1 could interact with LAPTM4B, which probably blocked the activation of PI3K-Akt signaling pathway (Data not shown) [43]. Notably, one seminal investigation showed that SMYD3 directly methylated MAP3K2 to activate MAPK signaling [44], thus SMYD3-mediated repressed EMP1 expression may be an additional regulation on Akt activation. More data are required to validate this idea.

It has been established that deregulated epigenome boosted tumor initiation and progression. Therefore, the dysfunctional epigenome machinery could be a promising therapeutic target. Several drugs, which target DNMT, HDAC, EZH2, DOTL1 and LSD1, are used in clinical practice or under extensive clinical trials [45, 46]. Although a few small inhibitors targeting SMYD3 have been developed, the investigations into these drugs are limited to pre-clinical models. We found that BCI-121 inhibited the proliferation in vitro and in vivo in GC cells, suggesting that SMYD3 might be a targetable enzyme in GC treatment. More importantly, SMYD3 could potentiate Akt signaling pathway in GC, pancreatic ductal adenocarcinoma, lung adenocarcinoma, breast cancer, colon cancer, and bladder cancer [44, 47–50]. Hyperactivated Akt signaling pathway is commonly detected across almost all human malignancies; however, targeting Akt itself is still under way [51]. Based on our and the others’ findings, we propose that the combination of SMYD3 inhibitor might be an alternative approach worthy of consideration.

Our findings collectively provide evidence that SMYD3 plays a significant role in the proliferation of GC cells, partially through the repression of a set of essential genes in an H4K20me3-dependent manner (Fig. 6G). Among the inhibited genes, EMP1 was identified, and its restoration led to the deactivation of the PI3K/Akt signaling pathway (Fig. 6G). Furthermore, our results suggest that pharmaceutical inhibition of SMYD3 activity could be a promising therapeutic target for GC treatment.

## Materials and methods

### Patients and tissue samples

Tissue samples were collected from 125 patients with GC who underwent curative gastrectomy at Tianjin Medical University Cancer Hospital (Tianjin, China) from January 2004 to September 2007. All these patients enrolled did not receive neoadjuvant therapy before gastrectomy. Follow-up was performed every 3–6 months and completed in September 2012. The median was 32.0 months (range: 3–72 months). GC and matched normal gastric mucosa specimens (*N* = 30) were collected from patients receiving curative gastrectomy in 2021 at Tianjin Medical University Cancer Hospital (Tianjin, China) to detect SMYD3 mRNA level. All experiments used these samples were approved by the Institutional Research Ethics Committee of Tianjin Medical University Cancer Institute and Hospital (Tianjin, China).

### Cell lines and cell culture

Human GC cell lines (NCI-N87, SNU-1, AGS and SNU-16) were obtained from American Type Culture Collection (VA, USA). HGC-27, SGC-7901, MGC-803, BGC-823, and human immortalized gastric epithelial cells (GES-1) were from the National Infrastructure of Cell Line Resource (Beijing, China). The cell line MKN45 was a gift from Prof. Hui Li from Department of Gastrointestinal Cancer Biology at Tianjin Medical University Cancer Institute and Hospital, Tianjin, China. HEK293T cells were generously provided by Prof. Zhihua Liu from the National Cancer Center/Cancer Hospital, Beijing, China. All cell lines were cultured in RPMI-1640 supplemented with 10% fetal bovine serum (FBS, Newzerum, Christchurch, New Zealand), except for HEK293T, which were cultured in DMEM containing 10% FBS, and AGS, cultured in F12K with 10% FBS. Cells were maintained in a cell incubator with 5% CO_2_ at 37 °C. All cells with no more than 20 continuous passages were used in this study. All cell lines were verified as *Mycoplasma* negative.

### Plasmids, lentivirus production, and generation of stable cell lines

The vector pLVX-IRES-neo and pSIH-H1-puro were generously provided by Prof. Zhihua Liu from the National Cancer Center/Cancer Hospital, Beijing, China. Lentivirus was produced by simultaneously introducing the indicated lentiviral vectors, psPAX2 (Plasmid #12260) and pMD2.G (Plasmid #12259) into HEK293T cells. The intact EMP1 ORF was cloned into pLVX-IRES-neo vector. Short hairpin RNA (shRNA) sequences targeting SMYD3 or EMP1 were engineered in pSIH-H1-puro vector. Empty pLVX-IRES-neo vector and pSIH1-H1-puro vector were used as negative controls. The shRNA sequences were as follows: 5’-CGCTACTGTTATTATGCTATT-3’ (shEMP1), 5’-AGCCTGATTGAAGATTTGATT-3’ (SMYD3-sh1) and 5’-GCTTCCCGATATCAACATCTA-3’ (SMYD3-sh2). Cells were incubated with the indicated lentivirus and polybrene (1 μl/ml) for 24 h. G418 (400 μg/ml) or puromycin (2 μg/ml) was used to establish stable cell populations.

### Gastric cancer dataset analysis

The analysis of SMYD3 and EMP1 mRNA expression in TCGA GC cohort was performed using online integrated tools (https://portal.gdc.cancer.gov/ and https://www.xiantao.love/).

### RNA extraction and RT-qPCR

Total RNA was extracted using RNAiso plus (Takara Bio, Shiga, Japan). The cDNAs were generated using GoScript™ Reverse Transcription Kit (Promega, Madison, WI, USA). The mRNA levels of all genes were determined on the QuantStudio 5 real-time PCR system (Applied Biosystems, Foster City, CA, USA) using TB Green Premix Ex TaqTM II (TaKaRa). GAPDH was used for data normalization, and the 2^−ΔΔCt^ method was used to evaluate the relative abundance of the indicated genes. The primer sequences are listed in Supplementary Table S1.

### RNA-sequencing and analysis

Total RNA was extracted from SMYD3-knockdown and control GC cells. The sequence and the data analysis were conducted by LC-Bio (Hangzhou, Zhejiang, China). Differentially expressed genes (DEGs) were defined as fold change >2 or fold change <0.5 and *p* < 0.05, and then Gene Ontology (GO) and Kyoto Encyclopedia of Genes and Genomes (KEGG) pathway enrichment analyses were done. All services were provided by LC Biotech Corporation (Hangzhou, China). The data are deposited under GSE214155 in GEO database.

### CCK-8 assay and colony formation assay

The cell suspension was seeded in 96-well plates with 1000 cells/well in sextuple. CCK-8 (Zeta Life, China) was added to the cell suspension at a ratio of 1:10 and incubated at 37 °C for 2 h. The absorbance at 450 nm was measured using a microplate reader (BioTek). The CCK-8 assay for cells treated with BCI-121(100 μM) was performed in a similar manner.

The same number of cells (1000 cells/well) from the control and the treated group were seeded into each well of 6-well plates and cultured at 37 °C for 12–14 days. The medium was changed at regular intervals until a macroscopic clone formed. The colonies were fixed using methanol for 15 min and stained with 0.1% crystal violet. Colone numbers were counted and the images were then captured.

### Immunohistochemistry

IHC staining was performed to examine the expression of SMYD3 and EMP1 in GC samples with the anti-SMYD3 antibody (Abcam, ab187149, 1:200) and the anti-EMP1 antibody (Abcam, ab230445, 1:100). The staining index (SI) was evaluated by the intensity and proportion of positively stained tumor cells as follows. Scores of staining intensities were: 0, negative; 1, weak; 2, moderate; 3, strong. Scores of positively stained cell proportion were: 0, no positive; 1, <10%; 2, 10%–35%; 3, 35%–75%; 4, >75%. Using this method, SI with possible scores of 0, 1, 2, 3, 4, 6, 8, 9, and 12 were obtained among the GC samples. High and low expression was then defined with the optimal cutoff value of 6.

### Chromatin immunoprecipitation (ChIP)

According to manufacturer’s instructions, ChIP was performed using ChIP-IT Express kit (Active Motif). For this experiment, we fixed cells with 1% formaldehyde for 10 min, quenched the reaction with glycine, and suspended the fixed cells in cold lysis buffer. This kit contains an enzyme shearing cocktail that was added to the chromatin to shear it, followed by EDTA to stop the reaction. To facilitate ChIP with specific antibody, the sheared chromatin was pre-cleared with agarose beads coated with Protein A/G. Following cell harvest and lysing according to the manufacturer’s instructions, every group’s lysates were incubated with 4 g of Anti-SMYD3 antibody (ab228015, Abcam), anti-H4K20me3 antibody (39671, Active Motif) or normal rabbit IgG (ChIP kit) overnight at 4 °C. We collected protein A/G agarose beads with antibody-bound protein/DNA complexes after centrifugation. After eluting, crosslinking, and treating DNA with proteinase K, the chromatin was incorporated into DNA. A PCR was performed to detect DNA that had been enriched by ChIP. The EMP1 promoter ChIP primers were as follows: P1-F, 5′-TCTGATAATTCCTGACAGTGAGC-3′; P1-R, 5′-TGTTTACTGAAGCCCATTCCT-3′; P2-F, 5′-CAGGCTGAAACCTTGTGTT-3′; P2-R, 5′-TGGGAGTGAGCCATCAATTC-3′.

### Protein extraction and western blotting

Cells were washed with pre-chilled PBS buffer and total proteins were extracted with RIPA buffer (Boster Biological Technology, China) supplemented with protease inhibitors (MCE, USA) and phosphatase inhibitors (MCE, USA). The cytoplasmic and nuclear proteins were collected using Minute™ Cytoplasmic and Nuclear Extraction Kit (Invent Biotechnologies, Inc., Beijing, China). GAPDH is a cytoplasmic loading control and LaminB1 as a nuclear loading control. BCA Protein Assay Kit (Thermo Scientific, USA) was used to quantify protein concentration. Denatured proteins were electrophoresed by vertical SDS-PAGE system (Bio-Rad, USA) and transferred to polyvinylidene fluoride (PVDF) membranes. Membranes were blocked with 5% nonfat milk buffer for 1.5 h and then incubated with primary antibodies overnight at 4 °C. Following washing with TBST, the membrane was incubated with secondary antibody for 2 h and was visualized using chemiluminescence reagent (Thermo Scientific, USA). The primary antibodies used for Western blot are as follows: rabbit anti-SMYD3 antibody (Abcam, ab187149, 1:1000), rabbit anti-EMP1 antibody (Abcam, ab230445, 1:1000), rabbit anti-H4K20me3 antibody (Abcam, ab177190, 1:1000), rabbit anti-Geminin antibody (Abcam, ab195047, 1:500), rabbit anti-Aurora A antibody (Abcam, ab52973, 1:50,000), rabbit anti-Histone H3 (phospho-S10) (CST, 53348S, 1:1000), rabbit anti-AKT1 (phosphor-S473) (Abcam, ab81283, 1:5000), rabbit anti-AKT (Abcam, ab8805, 1:5000), mouse anti-LaminB1 (proteintech, 66095, 1:1000) and mouse anti-GAPDH (Abcam, ab8245, 1:1000).

### Xenograft tumor model and tissue staining

Female 4-week-old Balb/c nude mice were purchased from Vital River Laboratories (Beijing, China) and housed under specific-pathogen-free (SPF) conditions. For the subcutaneous injection model, two groups (6 mice/group) were randomly divided. In total, 2 × 10^6^ SGC-7901 cells were suspended in 100 μl of PBS and injected into the dorsal flanks of the mice. For pharmaceutical inhibition experiment, 50 μl of BCI-121 (100 μM) was administrated intratumorally twice a week after 1 week of incubation [52]. The tumor volume was measured every 2 days using calipers. The tumor volume was calculated using the following formula: *V* = (width^2^ × length) × 0.5. The nude mice were then sacrificed, and their transplanted tumors were removed for other experiments. If the nude mice showed signs of pain during the process of tumor growth, such as significant weight loss, lethargy, or tumor rupture, the nude mice were sacrificed by cervical dislocation. The tumor was collected and weighed at the 18 days after the implantation. After being photographed, tumors were embedded into paraffin. Paraffin-embedded xenografts were then sliced into serial 6.0 μm sections for hematoxylin and eosin (H&E) staining and IHC staining using anti-Ki67 antibody (Abcam, ab16667, 1:200). All experimental animal procedures were carried out with the approval of the Institutional Animal Care and Research Advisory Committee of Tianjin Medical University (Tianjin, China).

### Chemical reagents

The SMYD3 inhibitor BCI-121 was purchased from MedChemExpress (Princeton, NJ, USA) and dissolved in dimethyl sulfoxide (DMSO) at a stock concentration of 20 mM.

### Statistical analysis

All experiments, with the exception of animal and IHC assays, were conducted independently at least twice. Statistical analysis was performed using a *t*-test to compare differences between two groups or a one-way analysis of variance to compare differences among three groups. The correlation analysis between SMYD3 expression and various clinicopathological characteristics was conducted using a chi-square test. The statistical analyses were performed using R software version 4.1.0 (The R Foundation for Statistical Computing, Vanderbilt University, Nashville, TN) and GraphPad Prism Version 8.0 software. Overall survival (OS) was using the Kaplan–Meier method and a log-rank test performed to determine significance. The multivariate analysis of OS was performed by the Cox proportional hazard model with forwarding step procedures. *p* value < 0.05 was considered statistically significant. *, ** and *** indicated *p* < 0.05, *p* < 0.01, *p* < 0.001, respectively.

## Supplementary information

aj-checklist

Western Blot original data file

Supplementary Table S1

## Data Availability

The GC datasets (GSE214155) from the GEO repository database (https://www.ncbi.nlm.nih.gov/gds) was used in this study. The data that support the findings of this study are available from the corresponding author upon reasonable request.
