# Supplementary material for: SMYD3 drives the proliferation in gastric cancer cells via reducing EMP1 expression in an H4K20me3-dependent manner
Source: Cell Death Dis. 2023 Jun 29;14(6):386. doi: 10.1038/s41419-023-05907-9 (PMC10310787; doi:10.1038/s41419-023-05907-9)

Figure 2A.

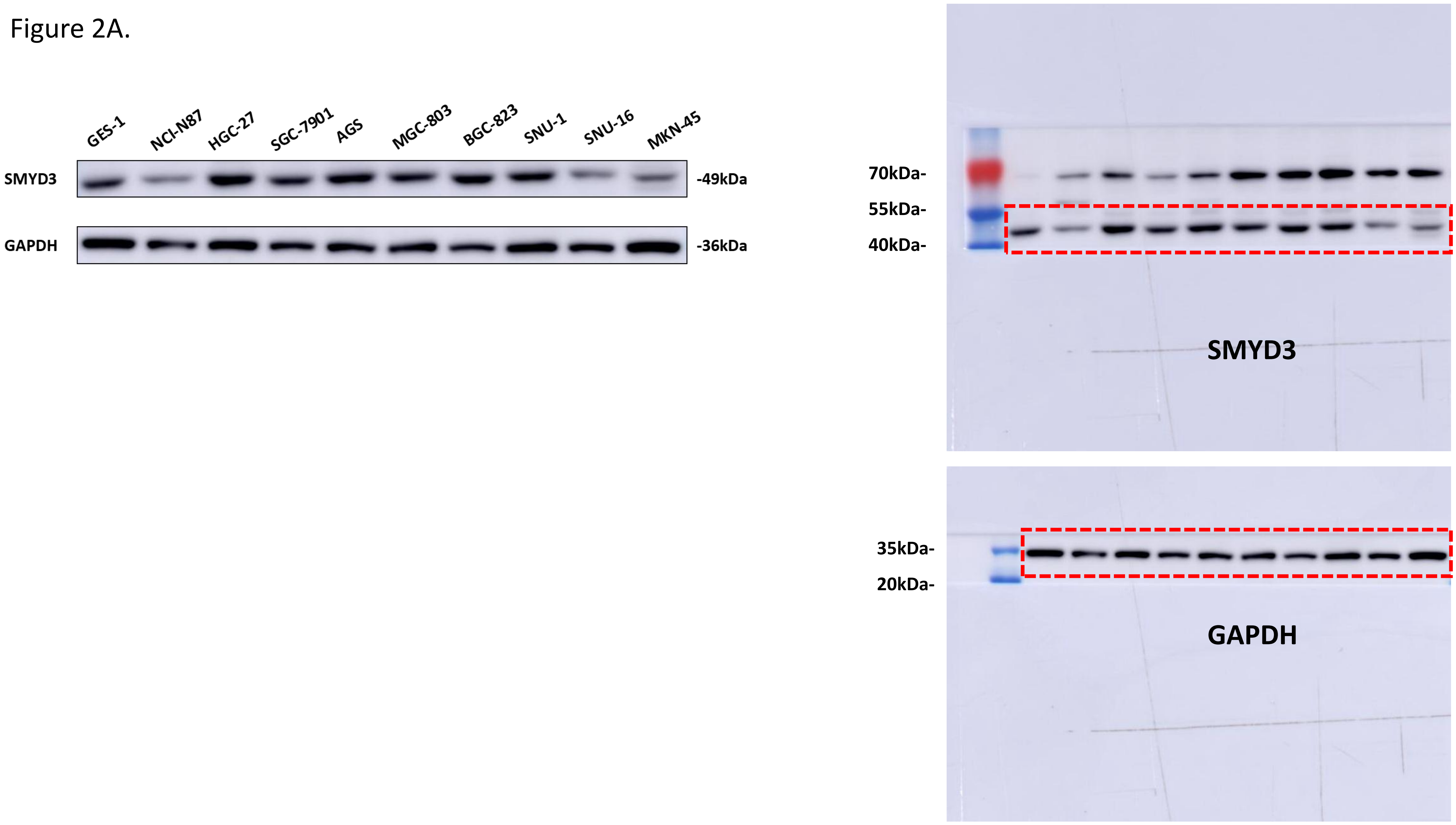
GES-1
NCI-N87
HGC-27
SGC-7901
AGS
MGC-803
BGC-823
SNU-1
SNU-16
MKN-45
SMYD3
-49kDa
GAPDH
-36kDa
70kDa-
55kDa-
40kDa-
SMYD3
35kDa-
20kDa-
GAPDH

Figure 2B.（HGC-27）

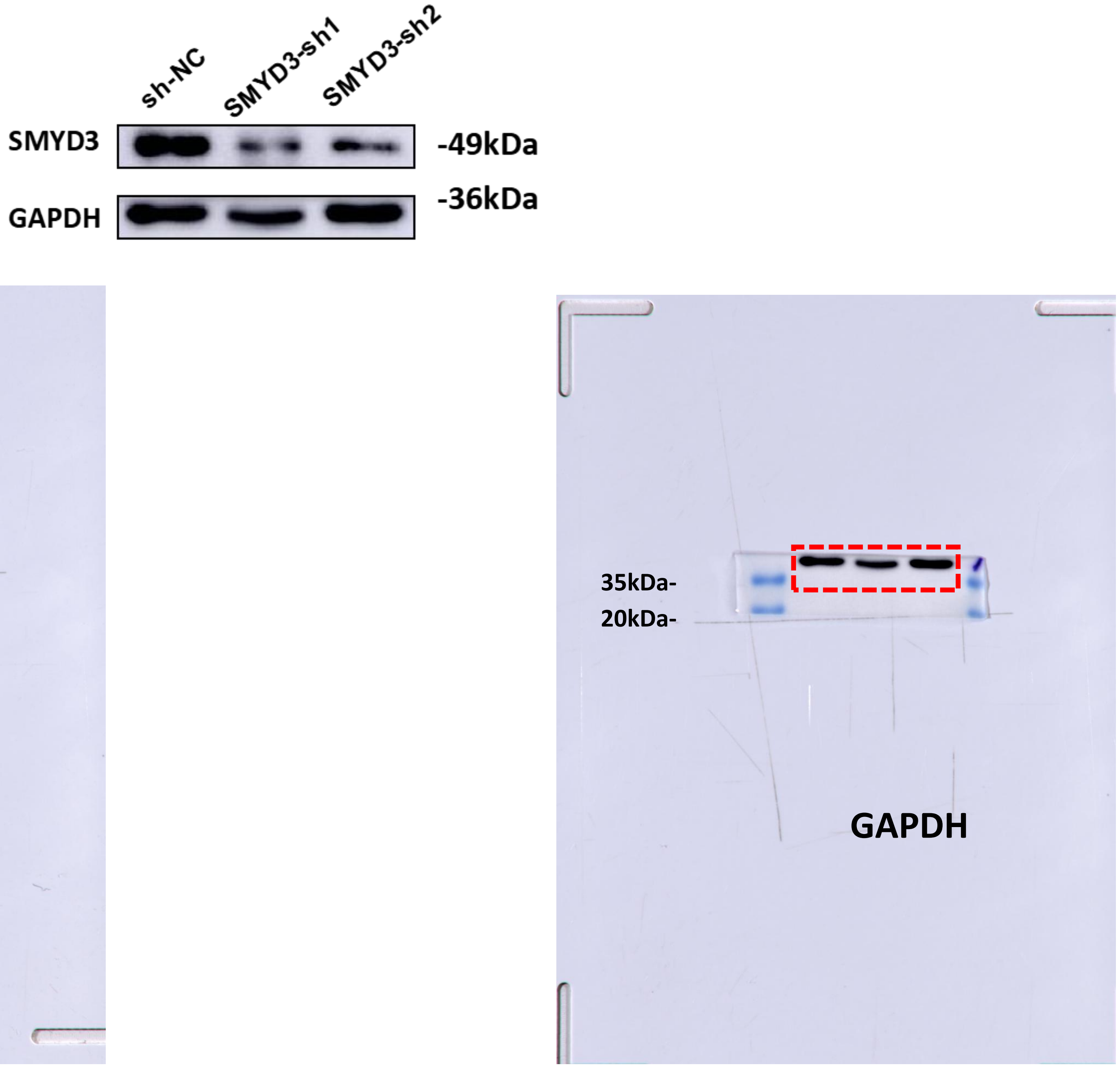

Figure 2B.（SGC-7901）

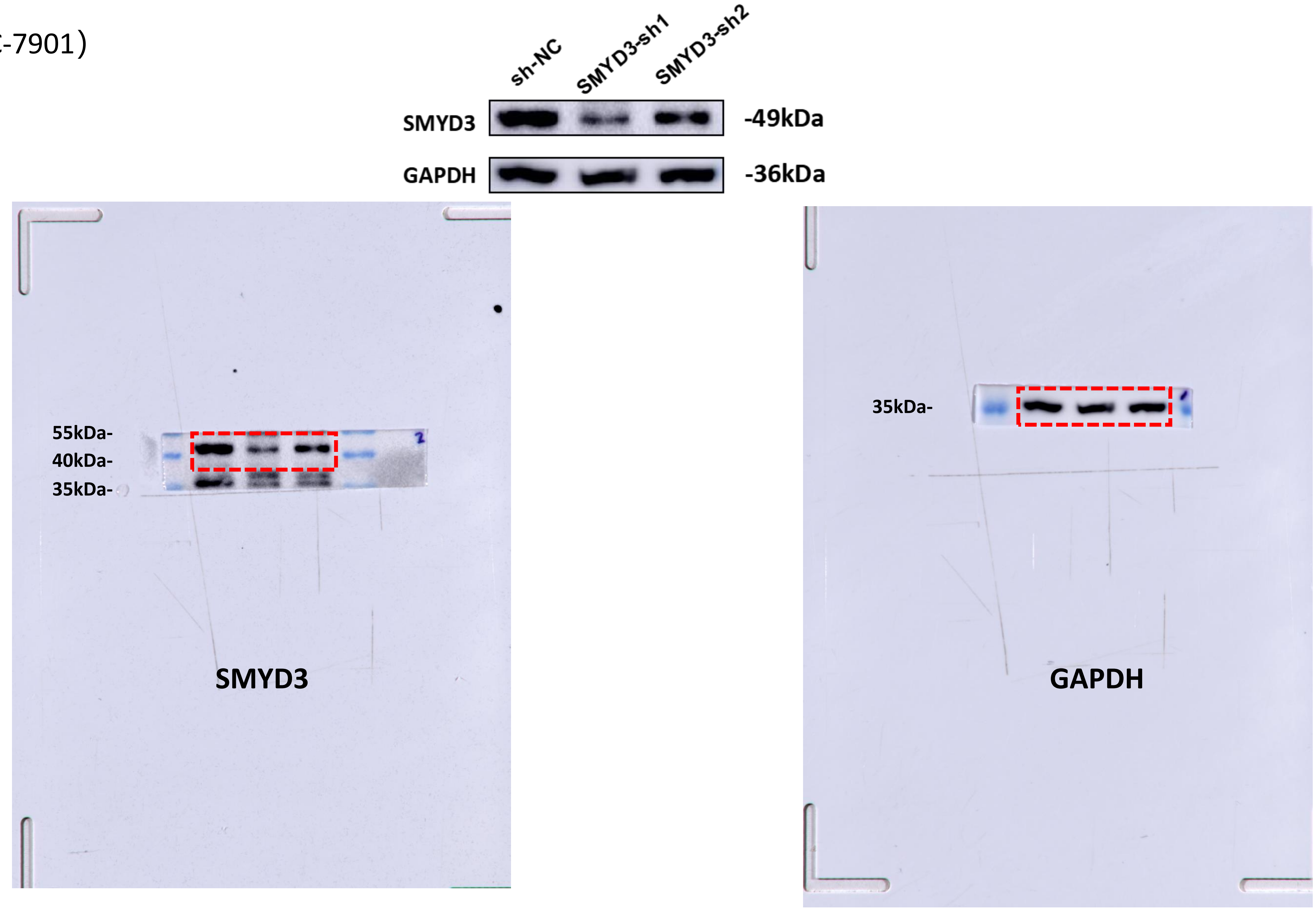

Figure 2H. （HGC-27）

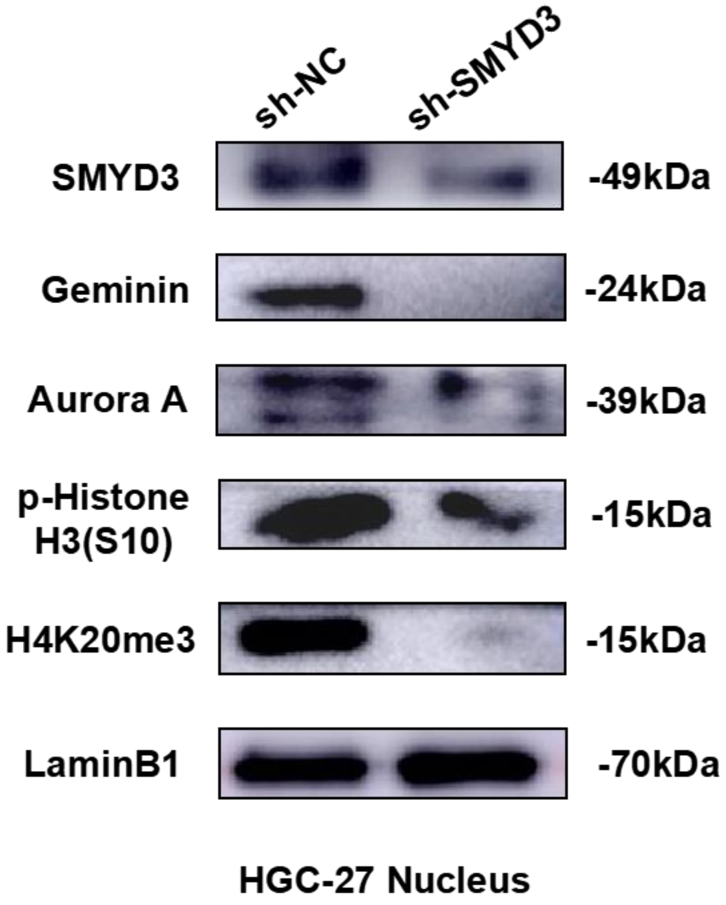

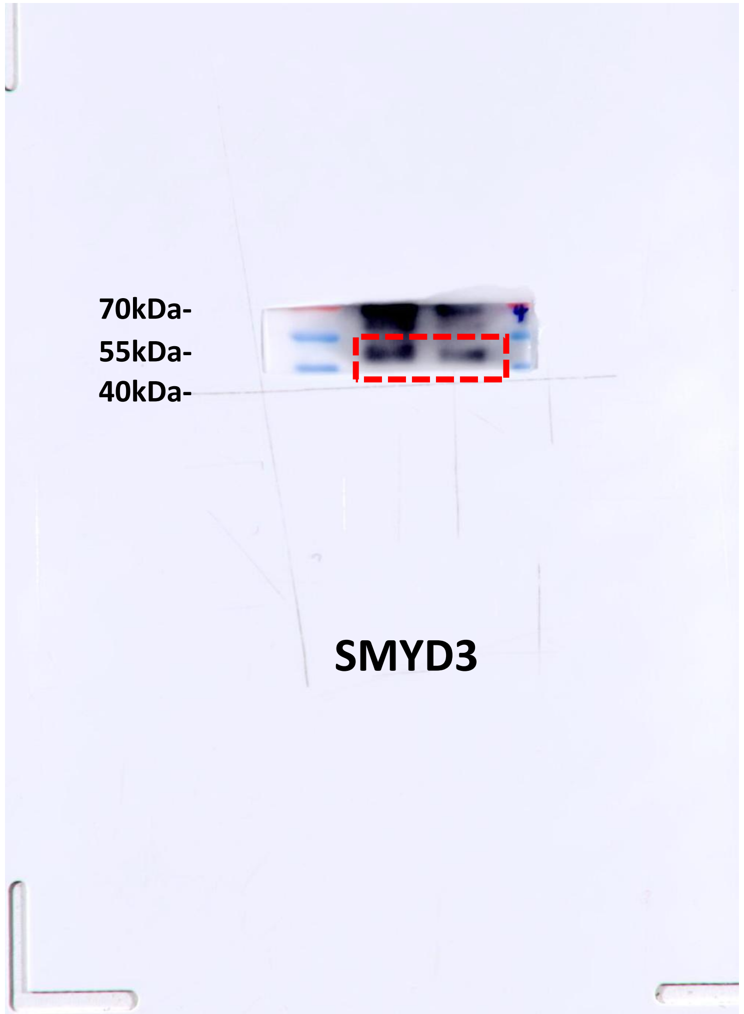

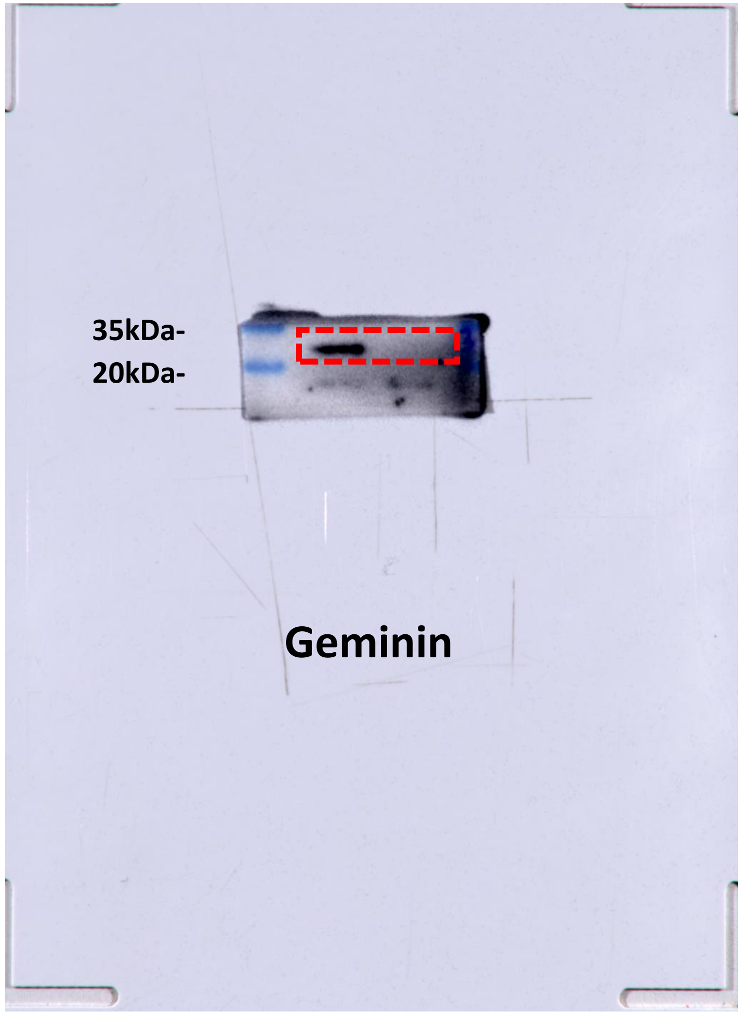

Figure 2H.（HGC-27）

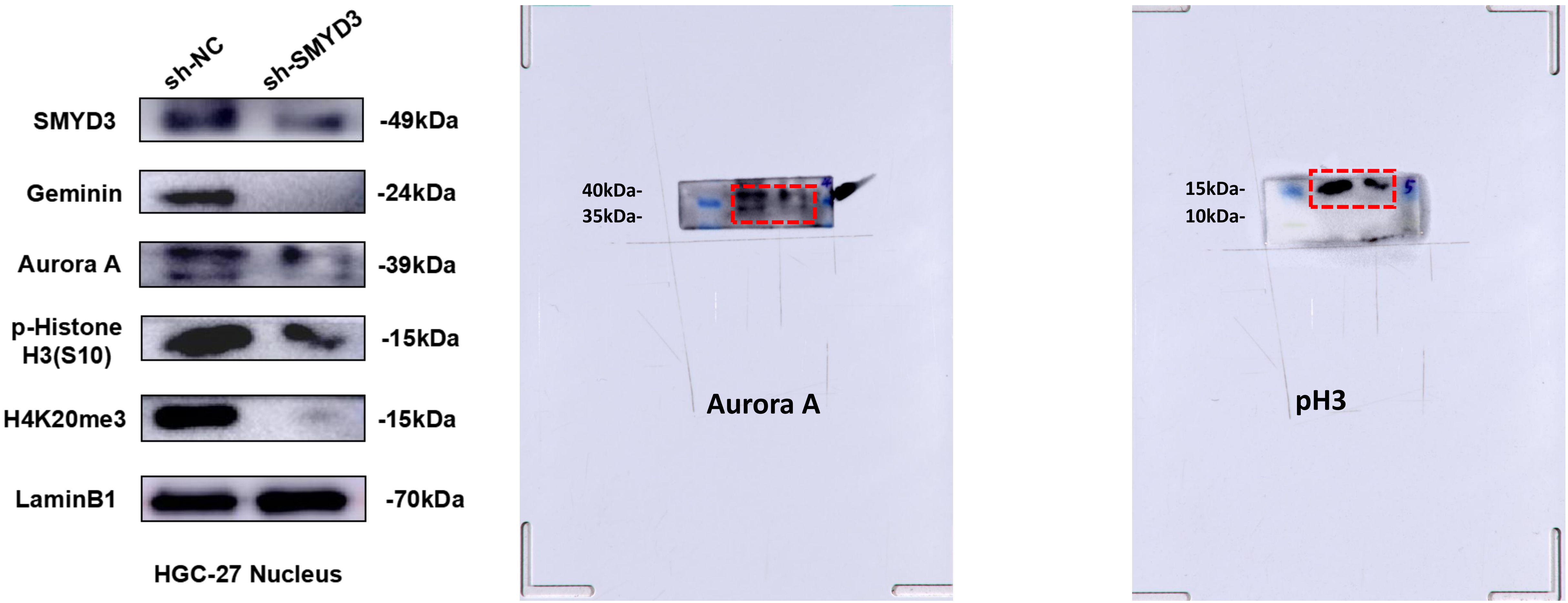

Figure 2H.（HGC-27）

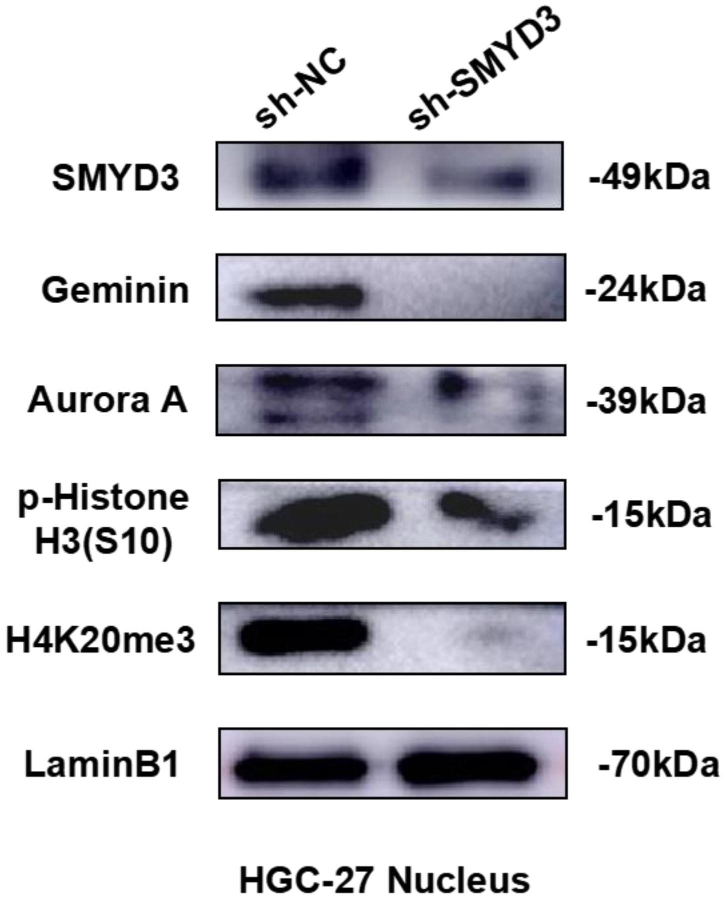

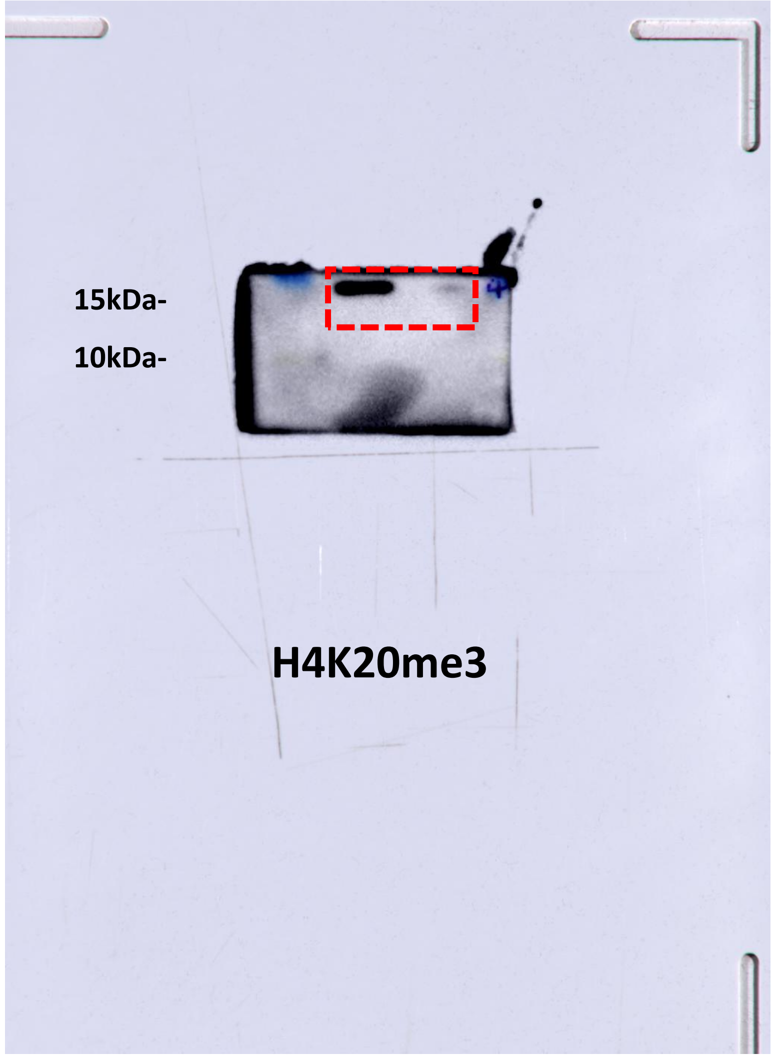

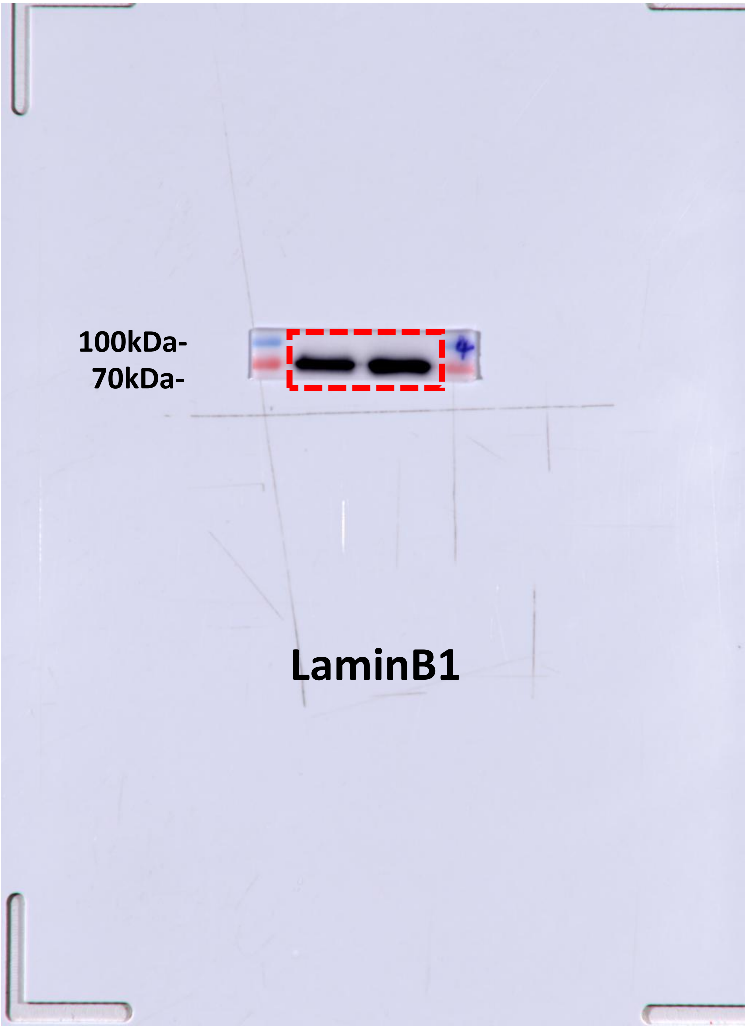

Figure 2H. （SGC-7901）

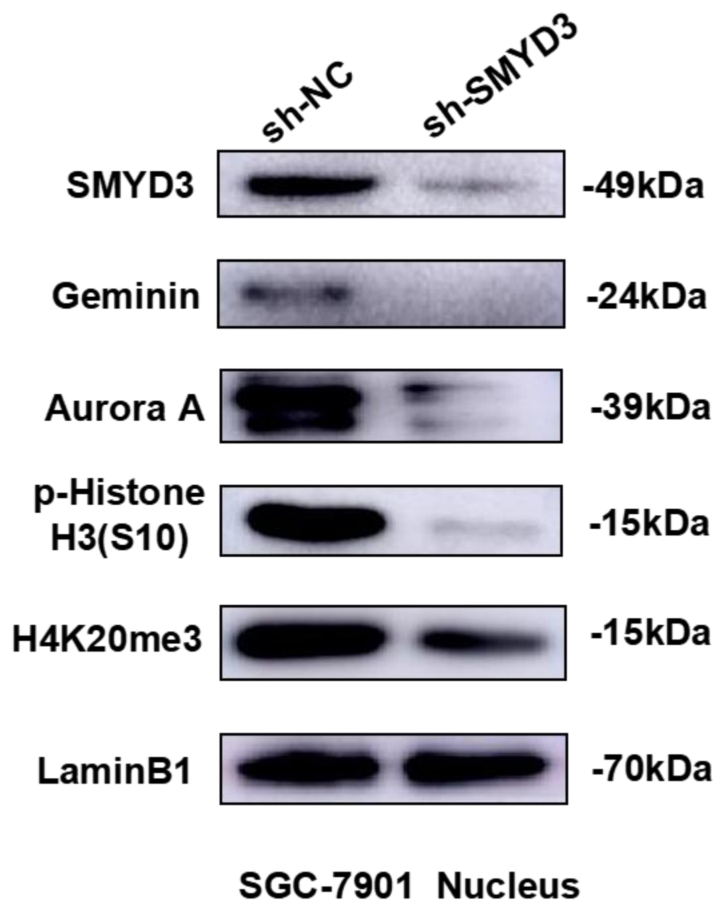

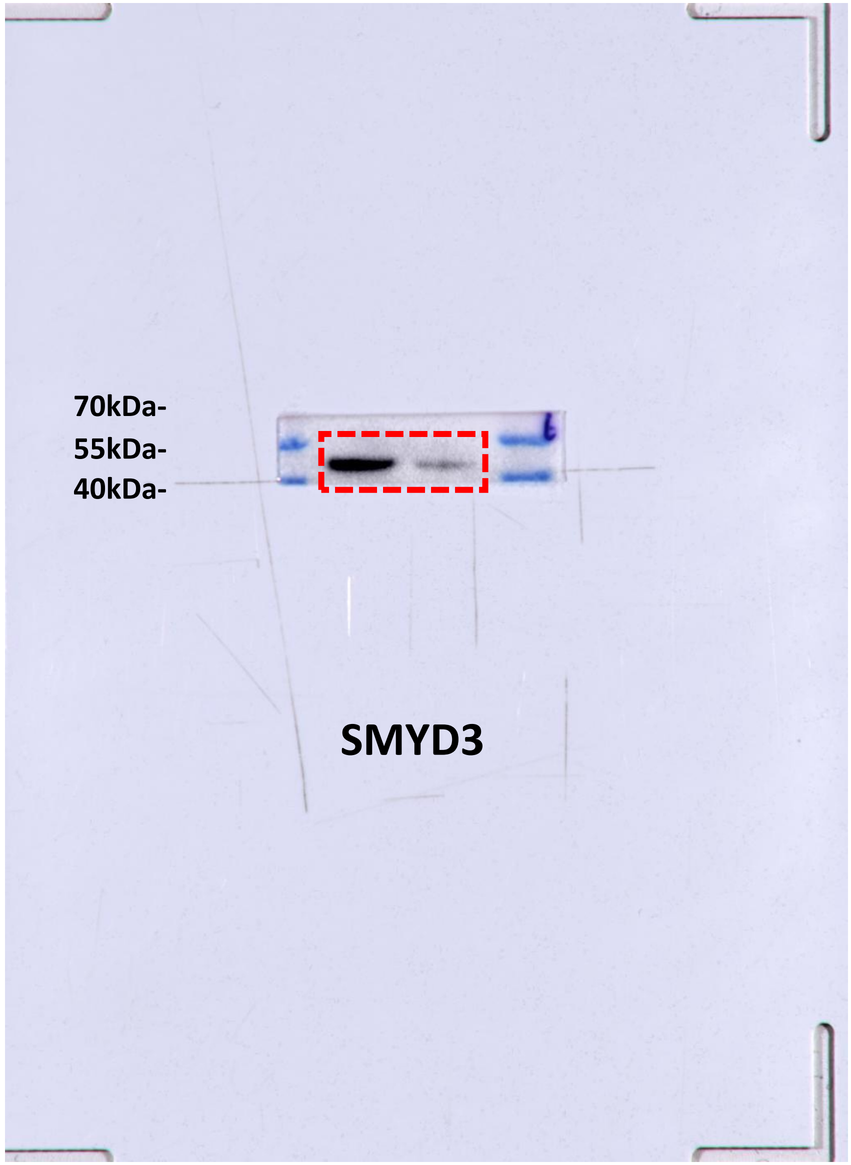

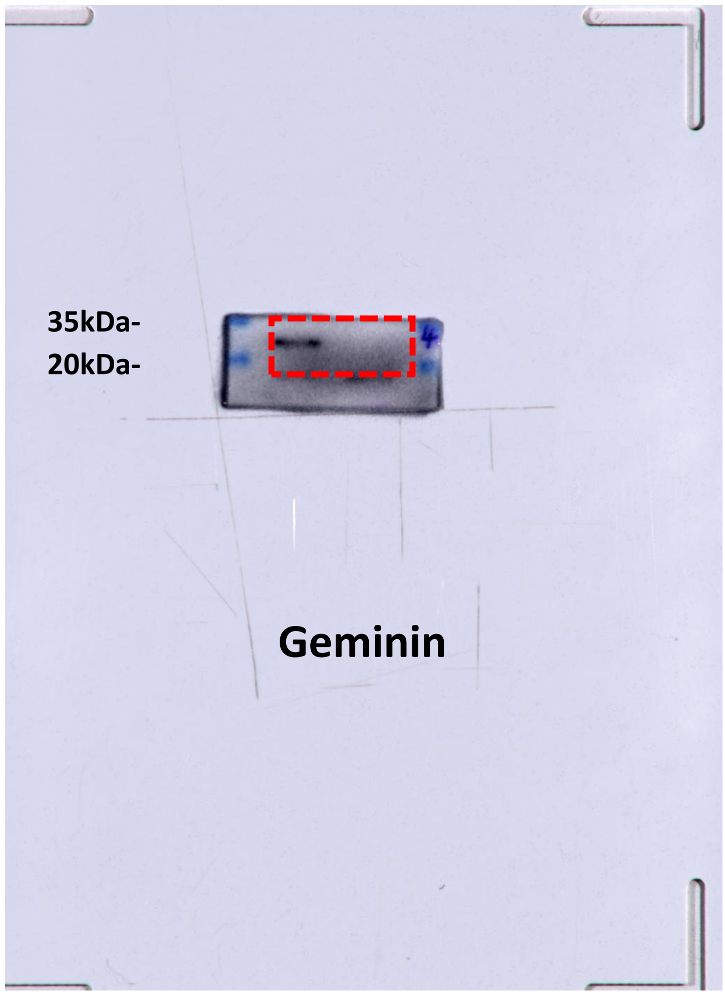

Figure 2H. （SGC-7901）

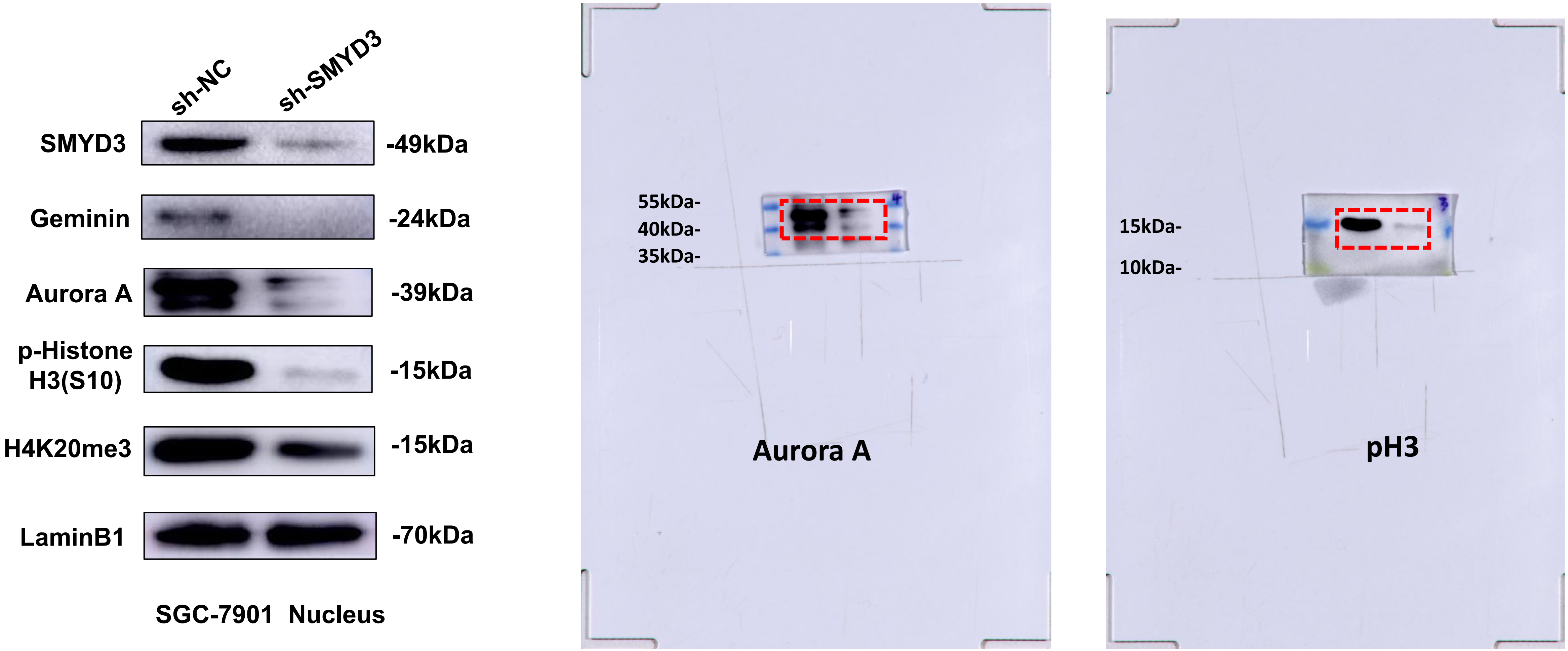

Figure 2H. （SGC-7901）

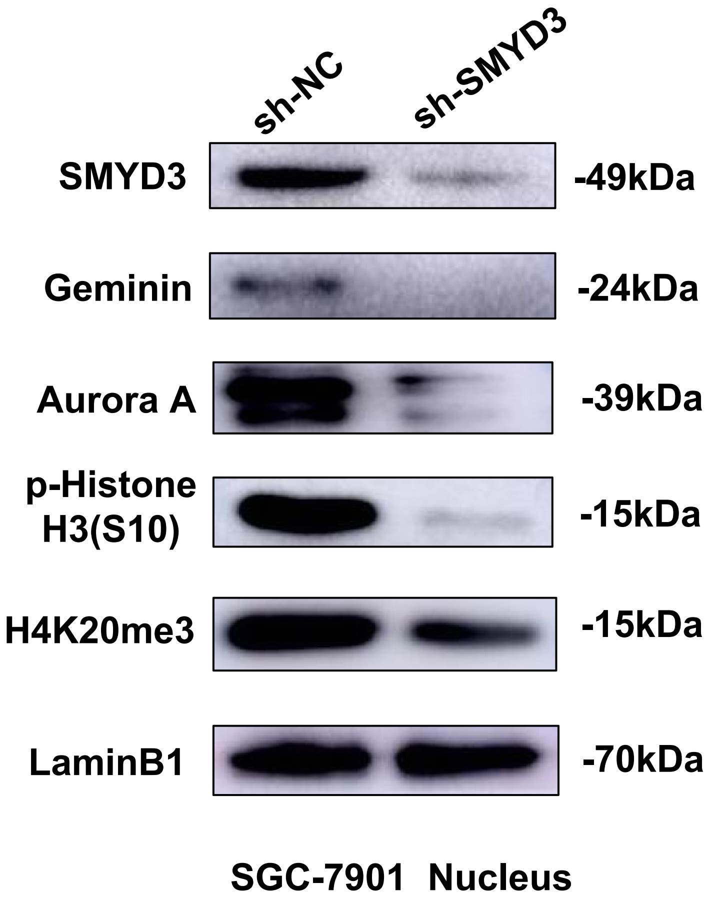

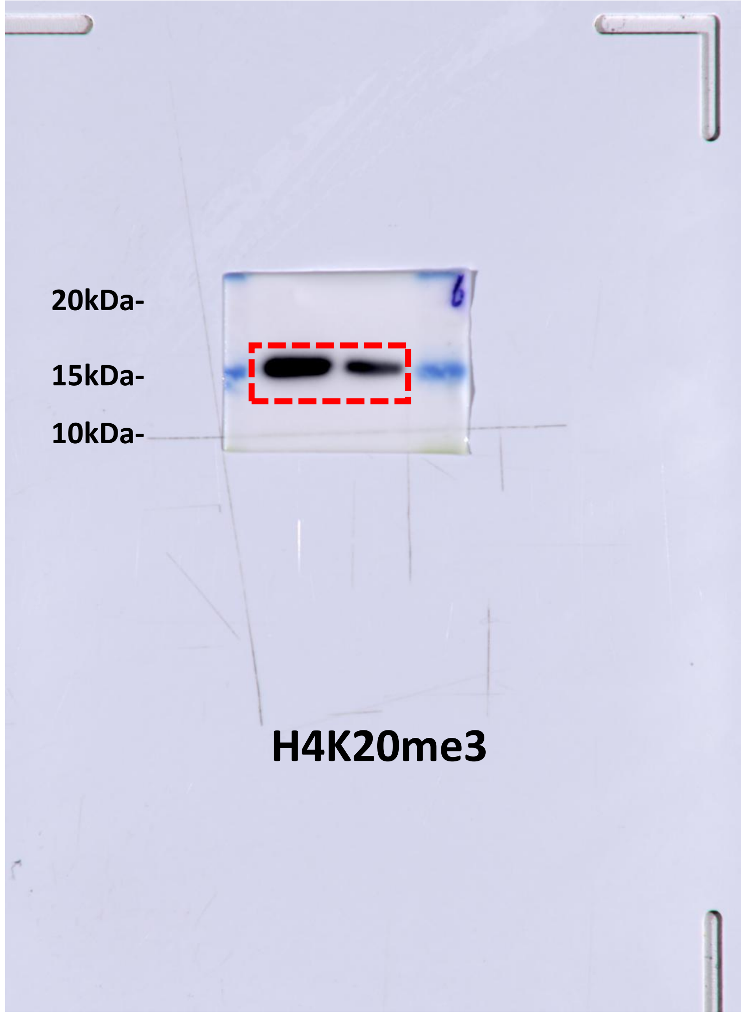

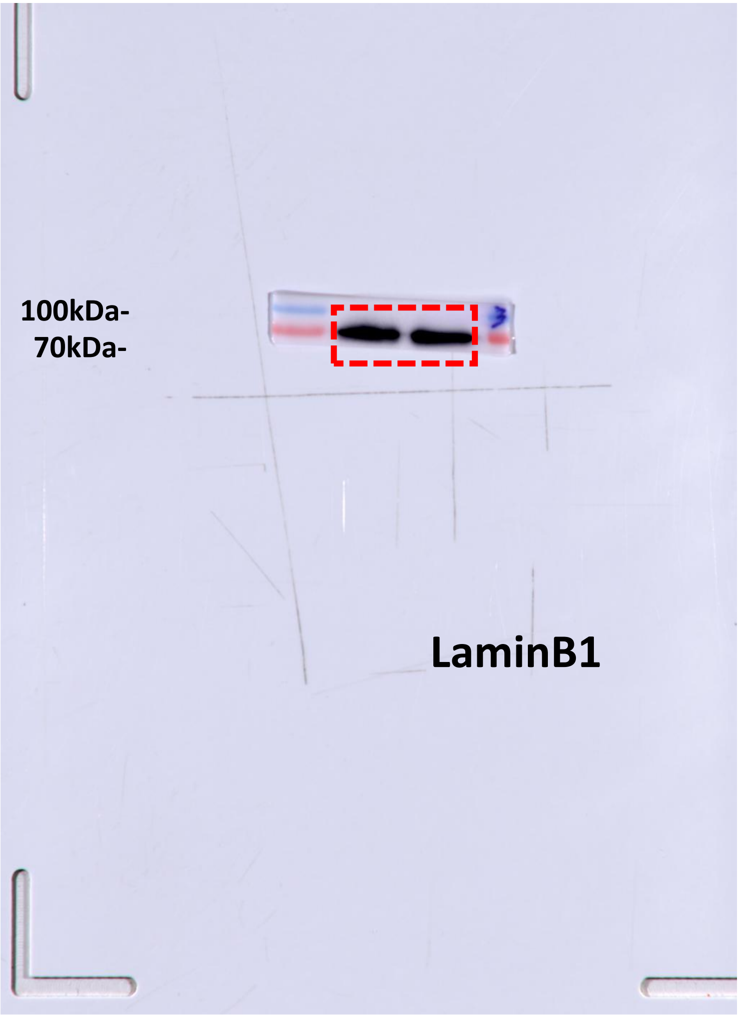

Figure 3C.（HGC-27）

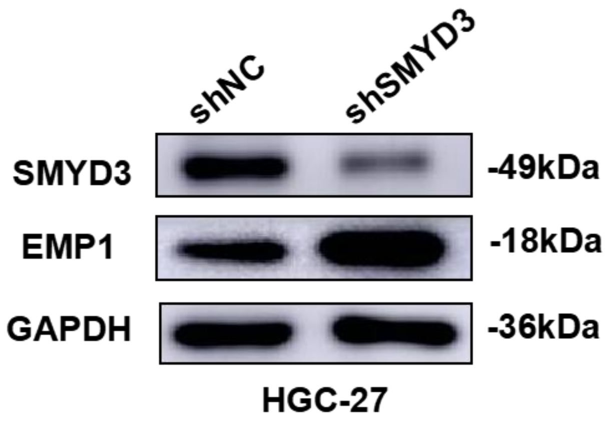

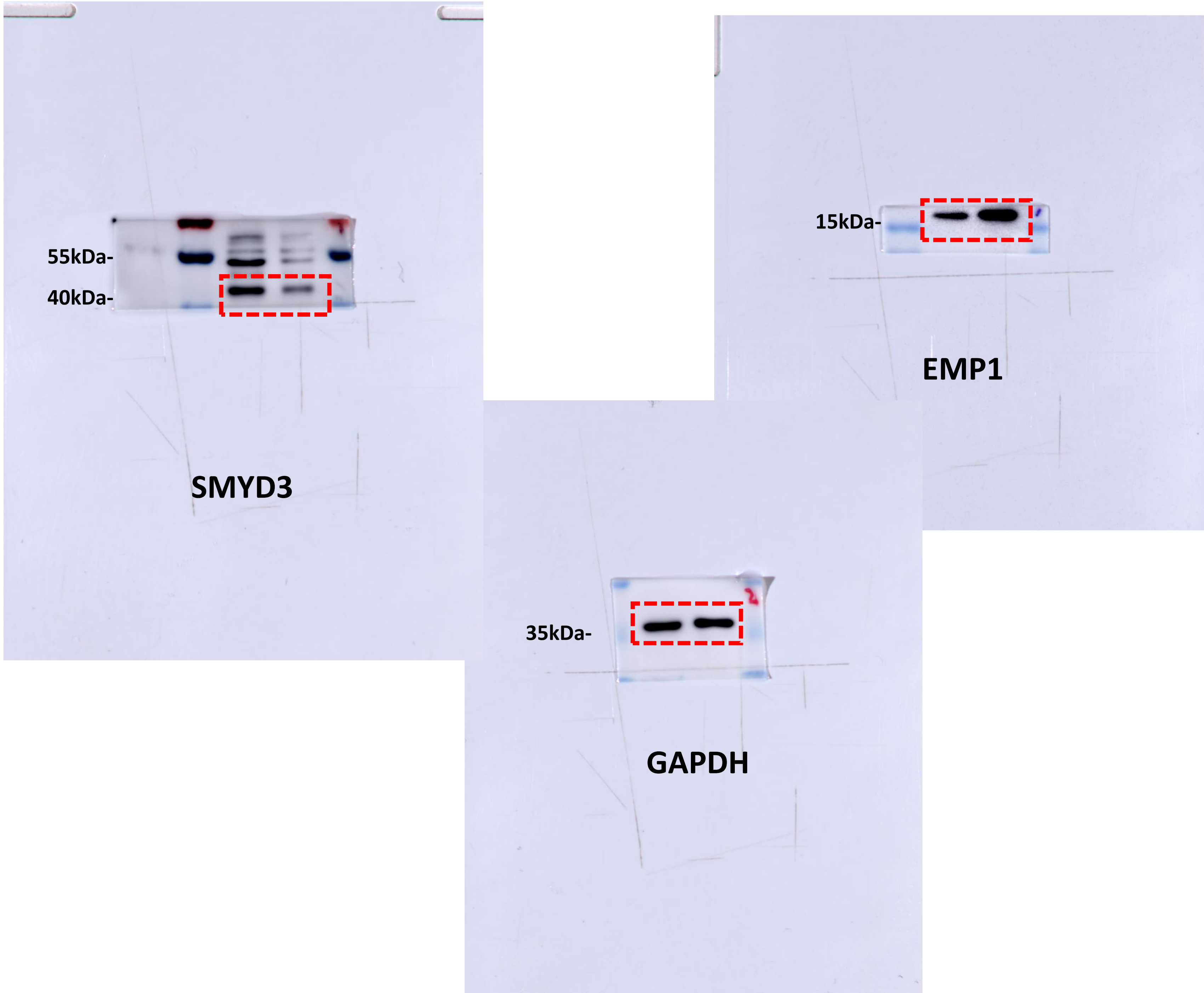

Figure 3C. （SGC-7901）

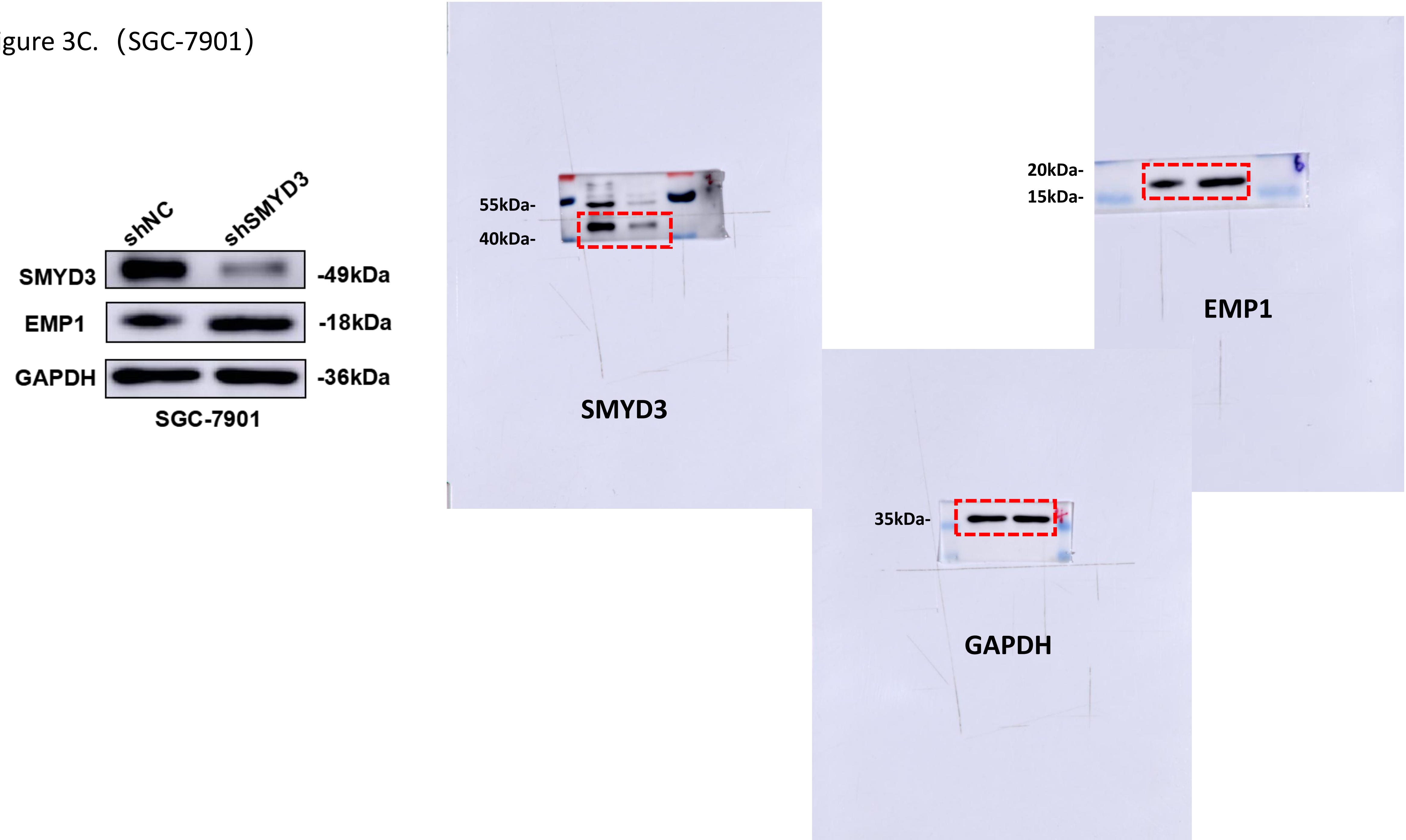

Figure 3H.

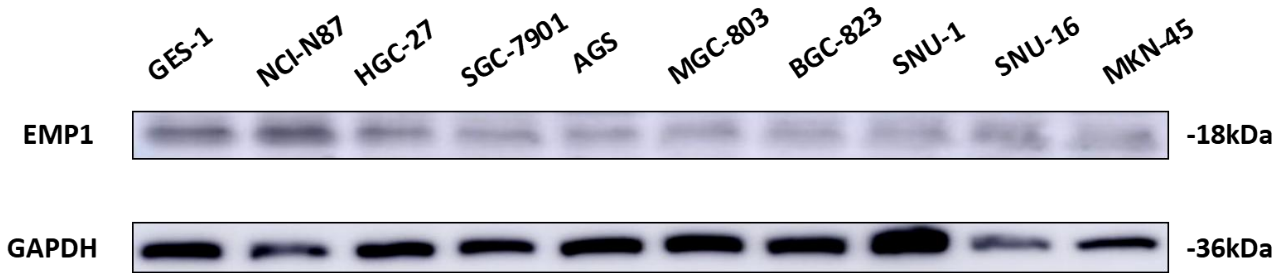
GES-1
NCI-N87
HGC-27
SGC-7901
AGS
MGC-803
BGC-823
SNU-1
SNU-16
MKN-45
EMP1
GAPDH
-18kDa
-36kDa

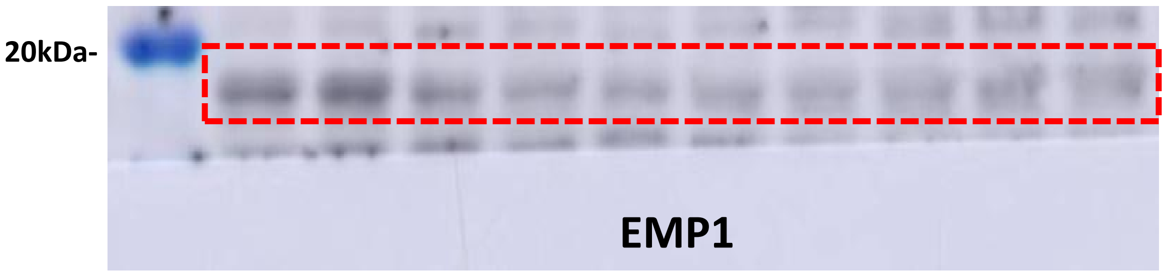
20kDa-
EMP1

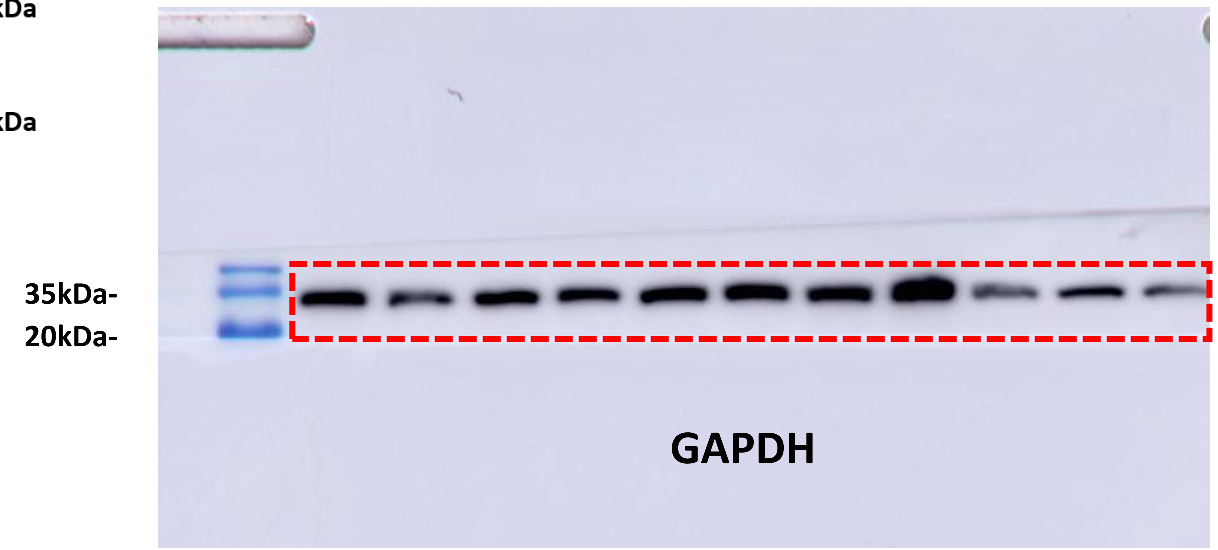
35kDa-
20kDa-
GAPDH

Figure 4D. （HGC-27）

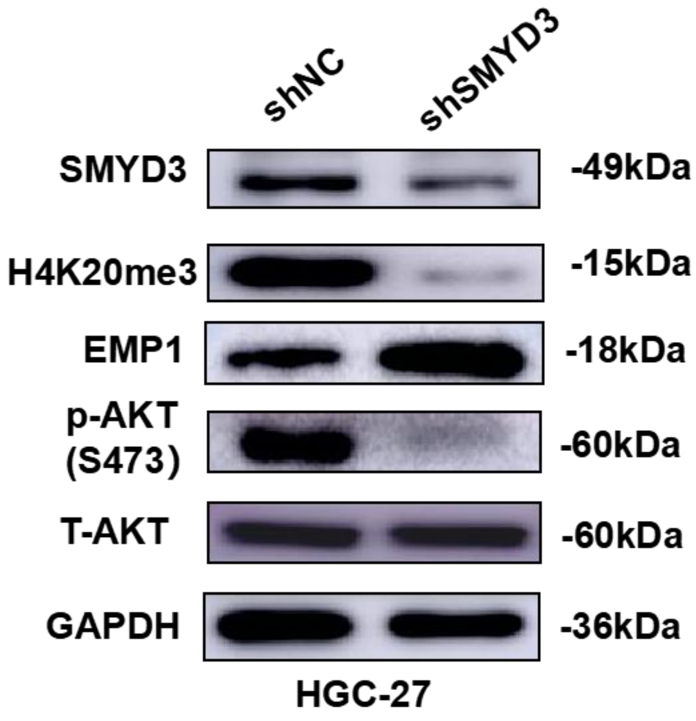

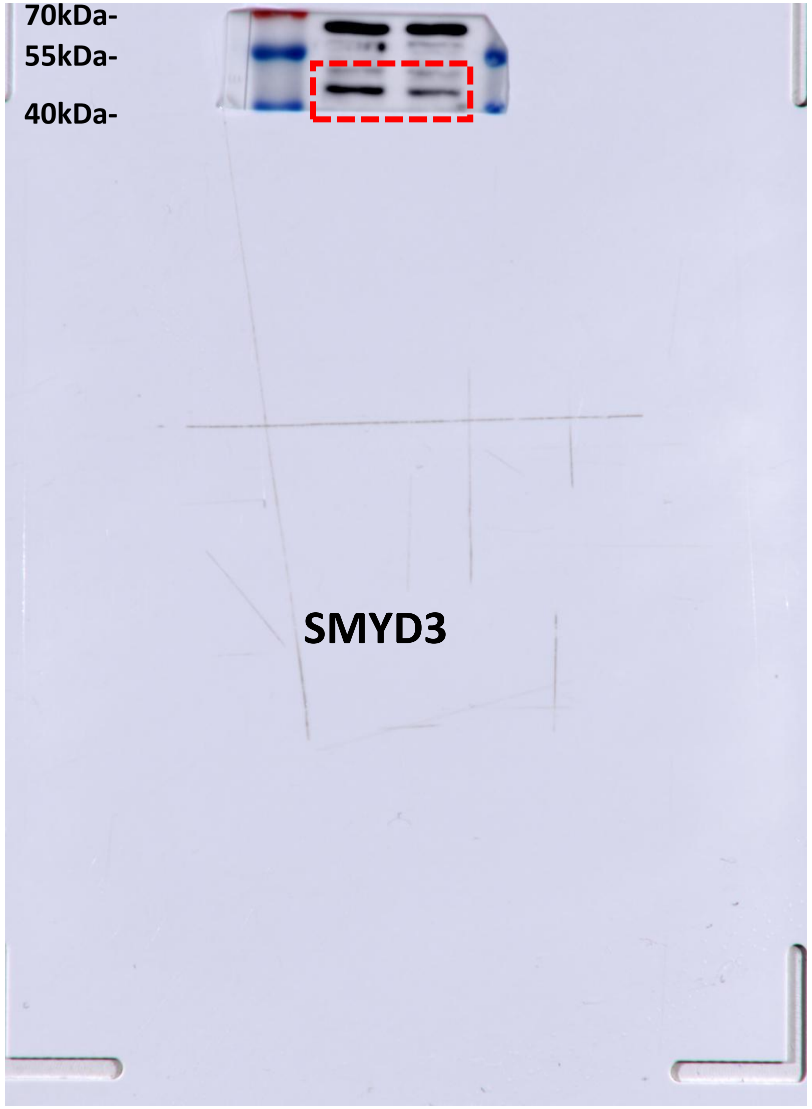

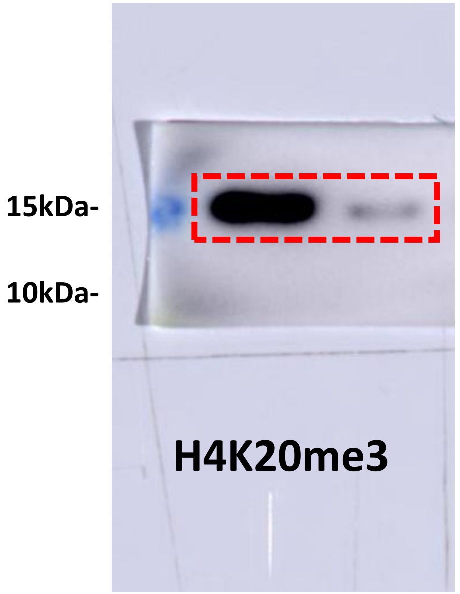

Figure 4D.（HGC-27）

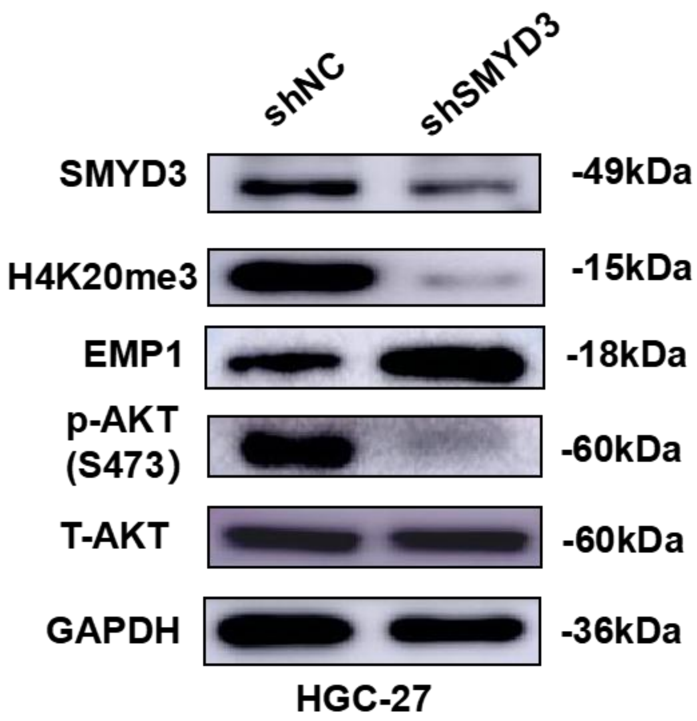

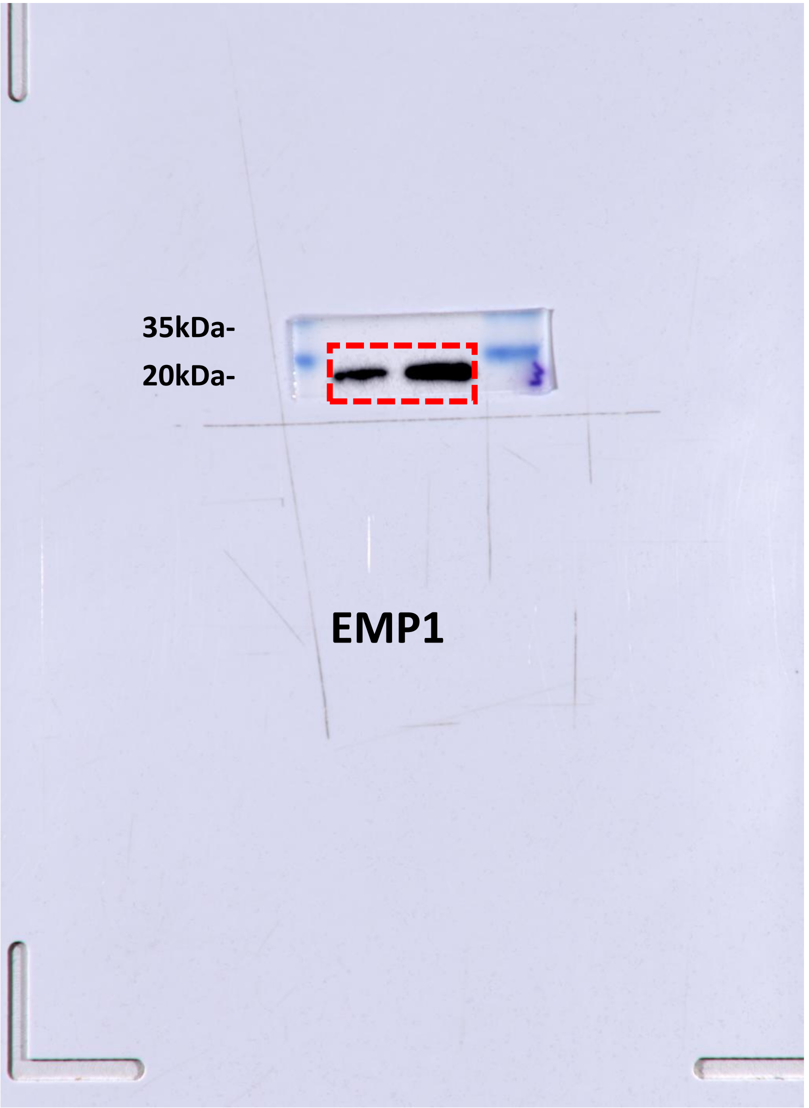

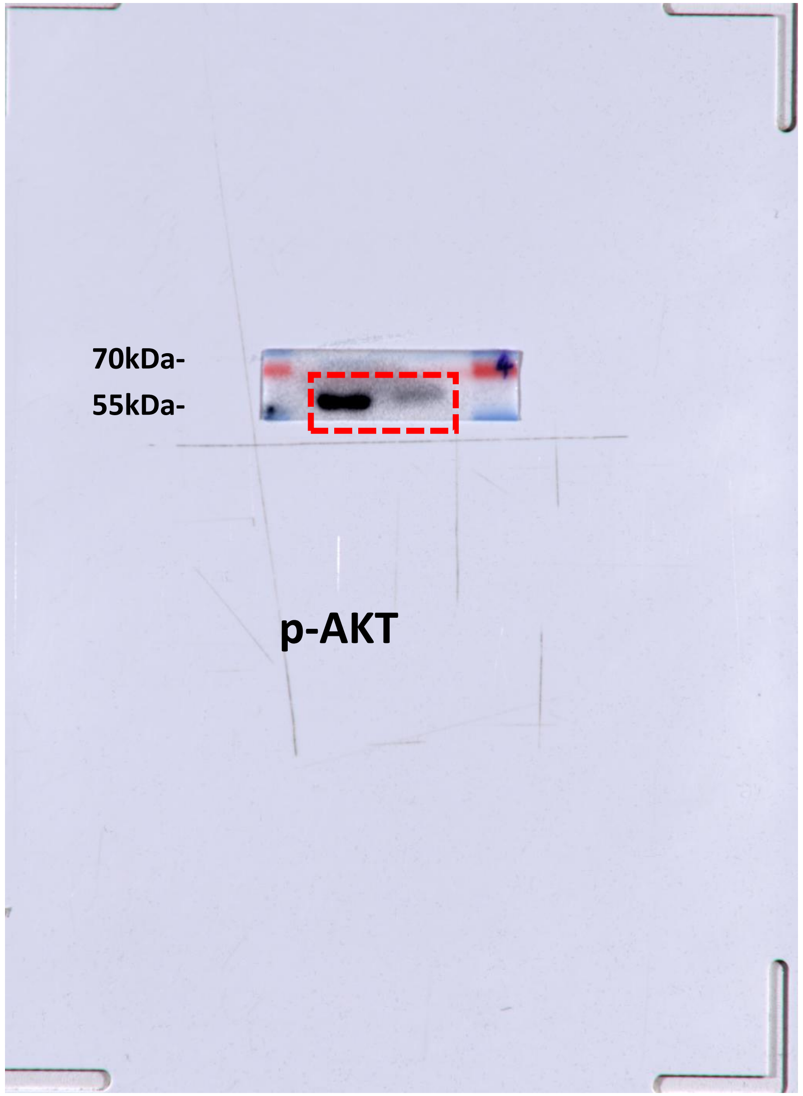

Figure 4D. （HGC-27）

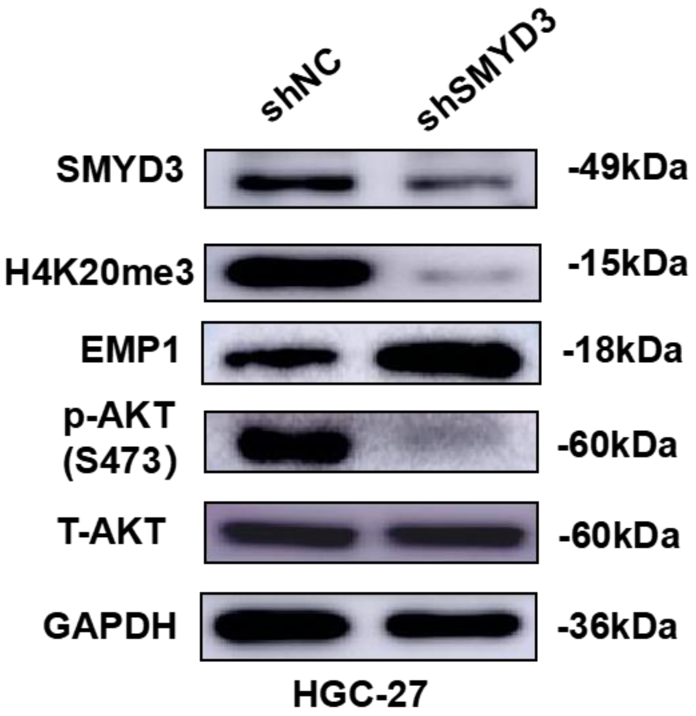

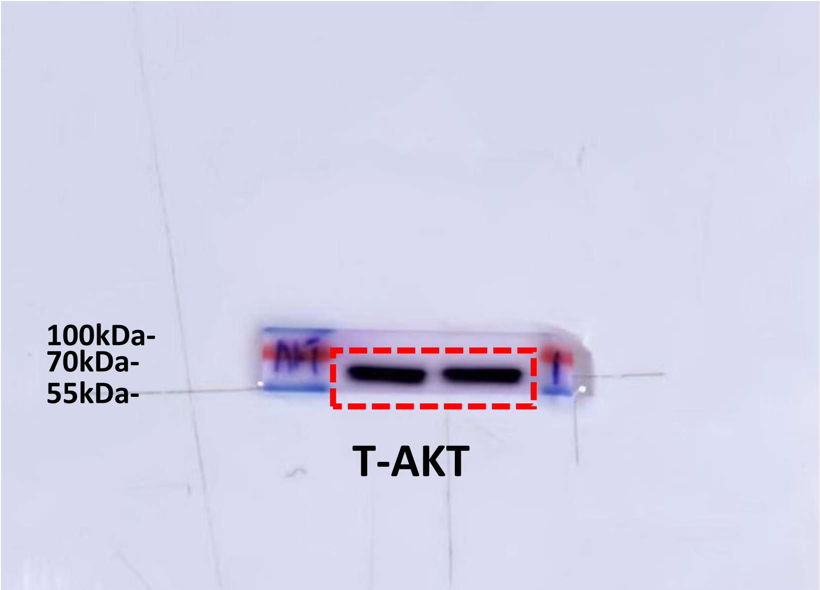

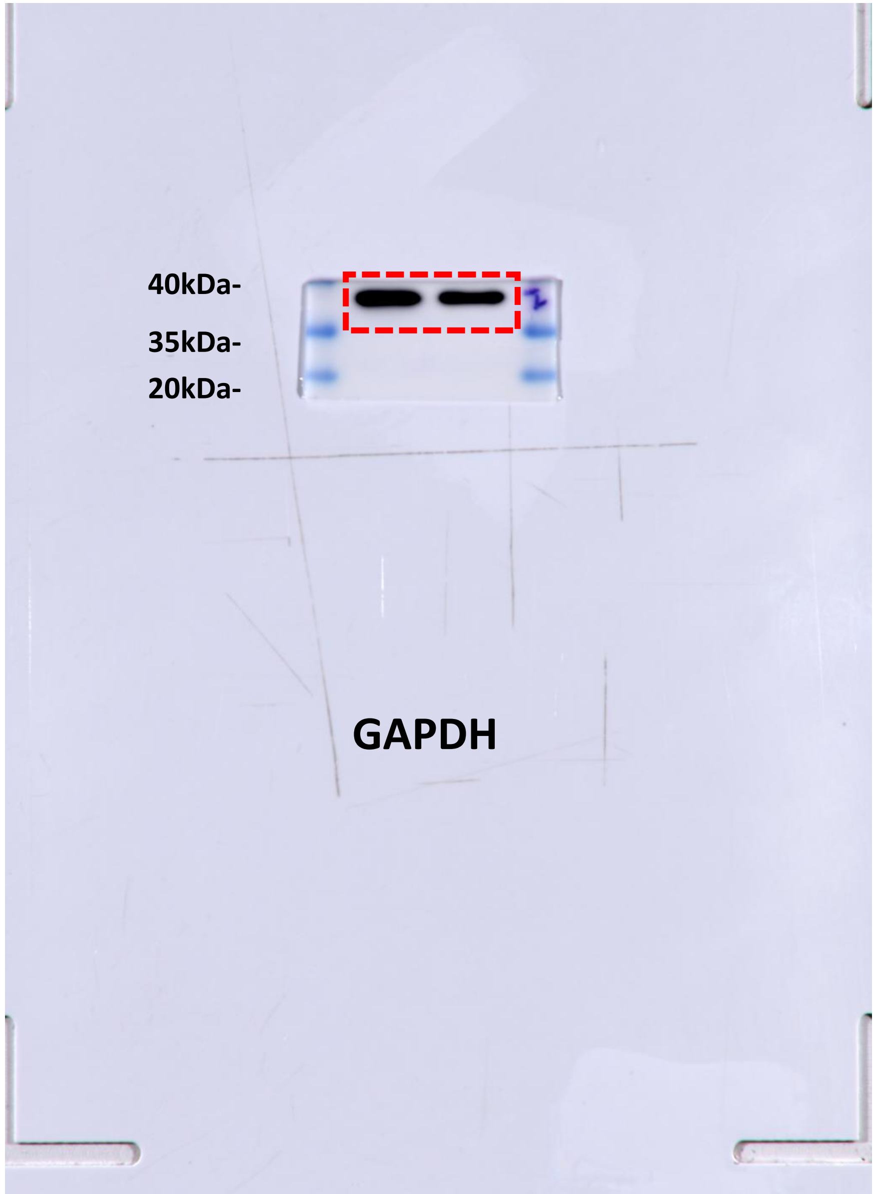

Figure 4D. （SGC-7901）

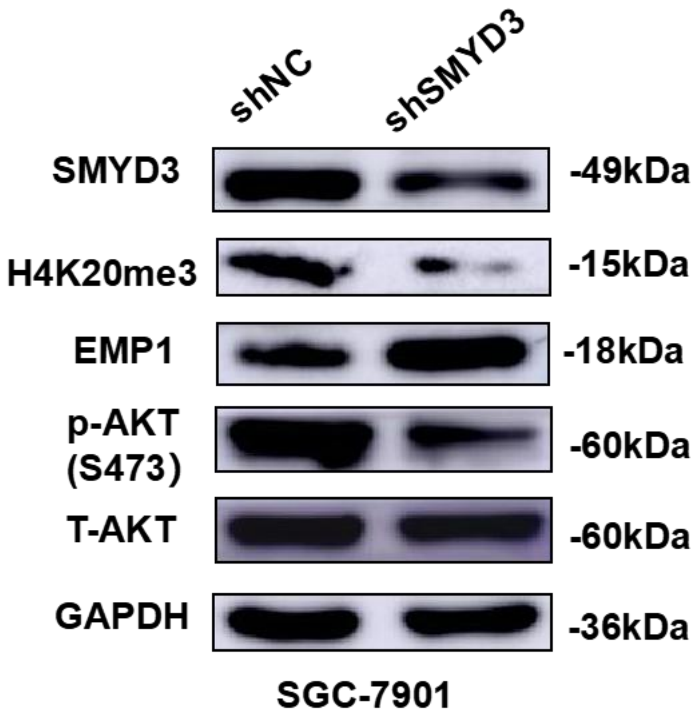

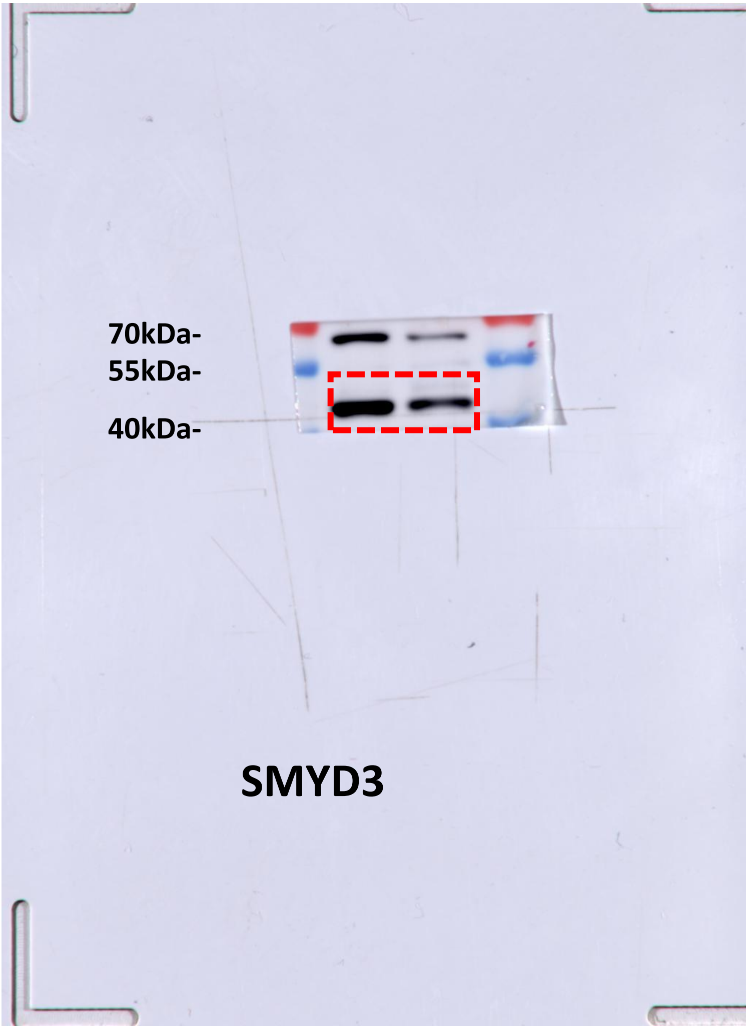

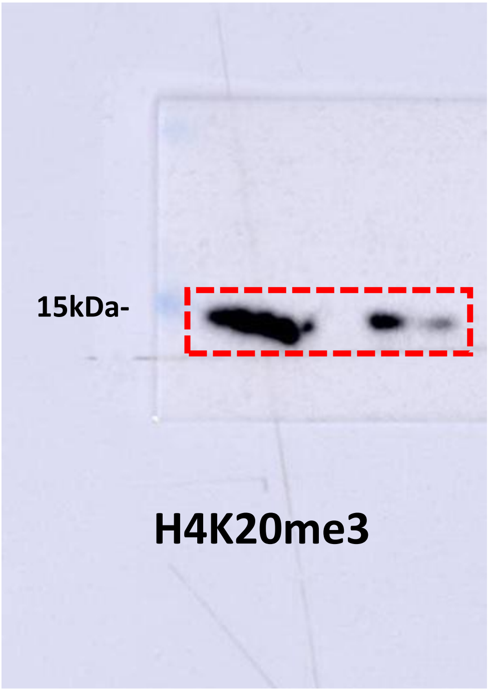

Figure 4D. （SGC-7901）

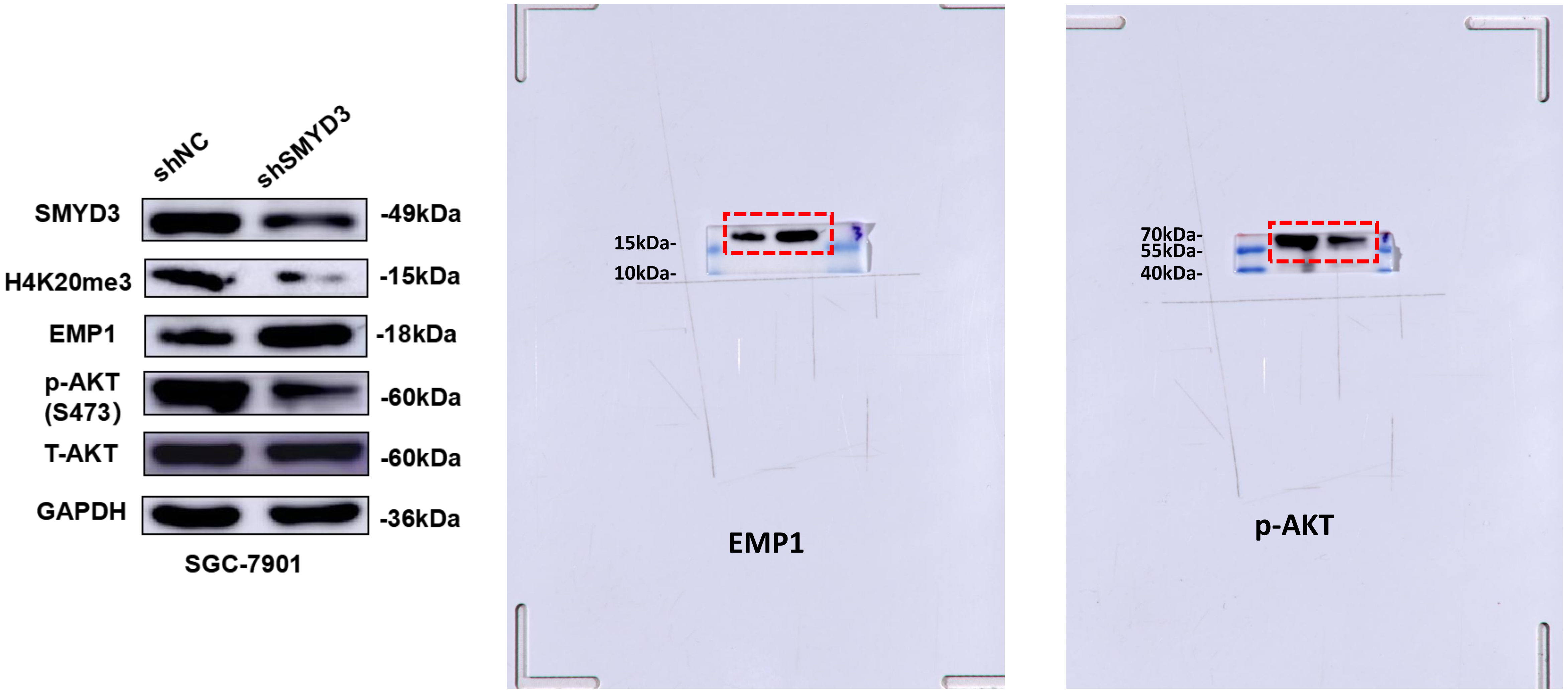

Figure 4D.（SGC-7901）

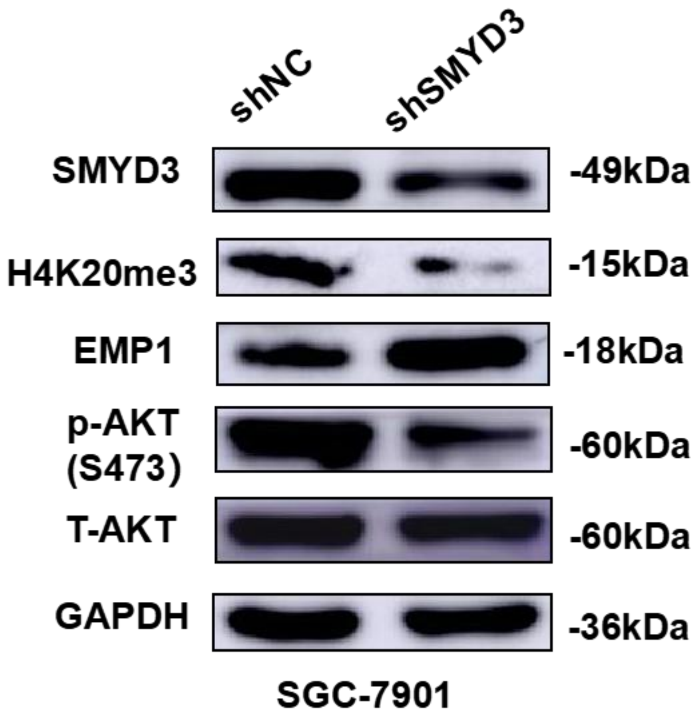

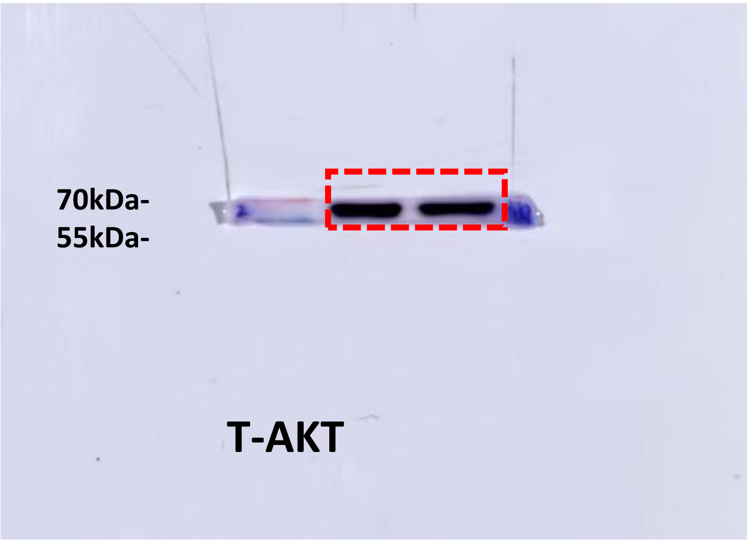

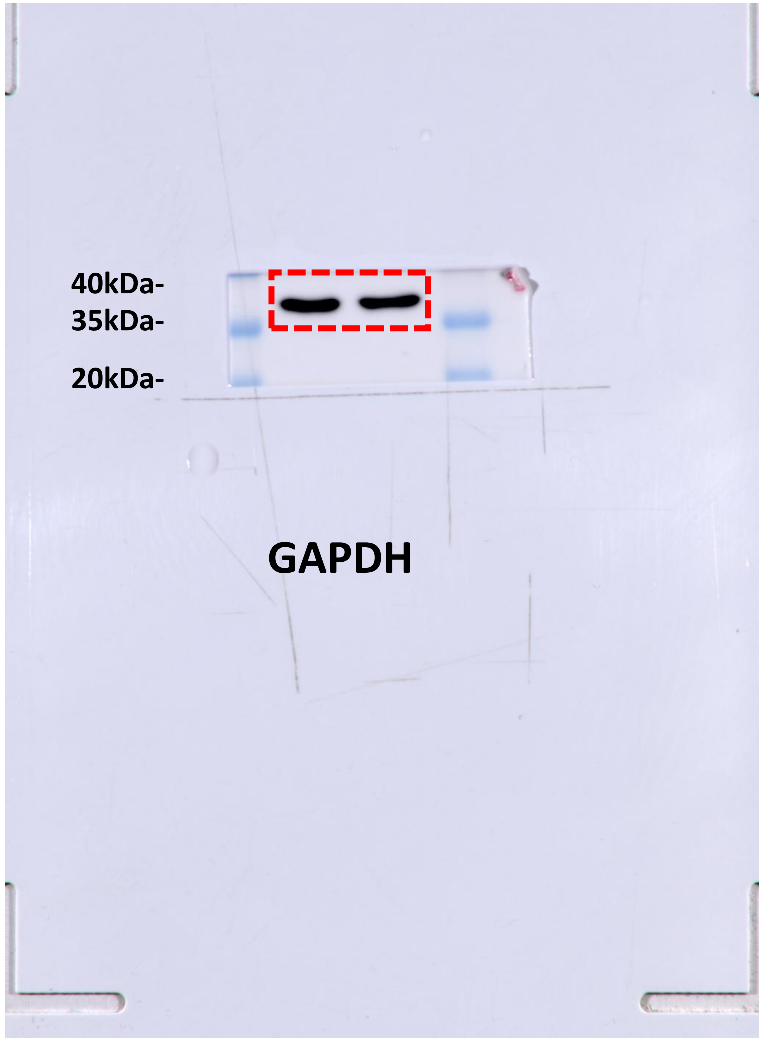

Figure 4E. （HGC-27）

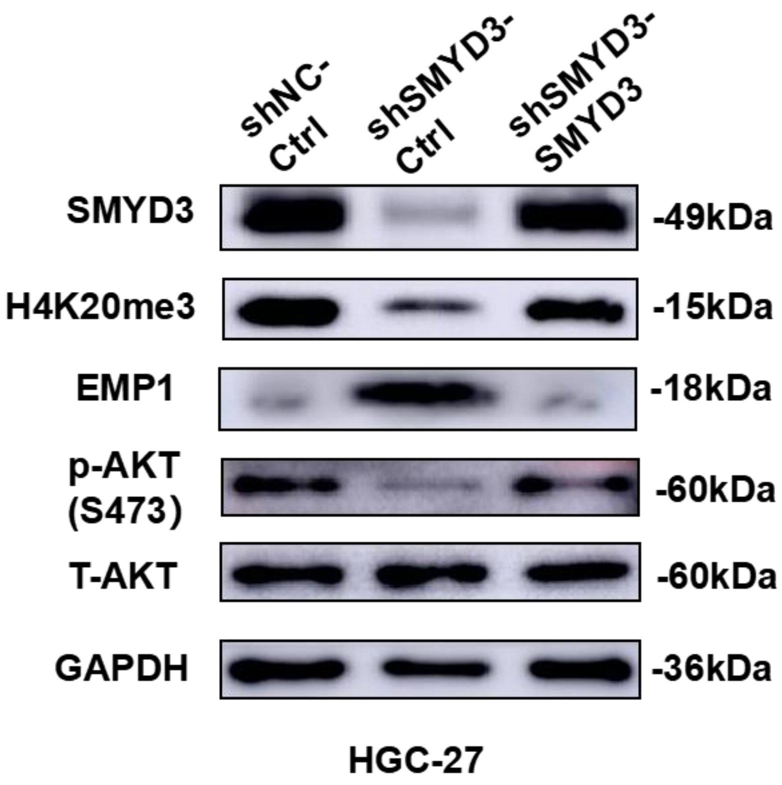

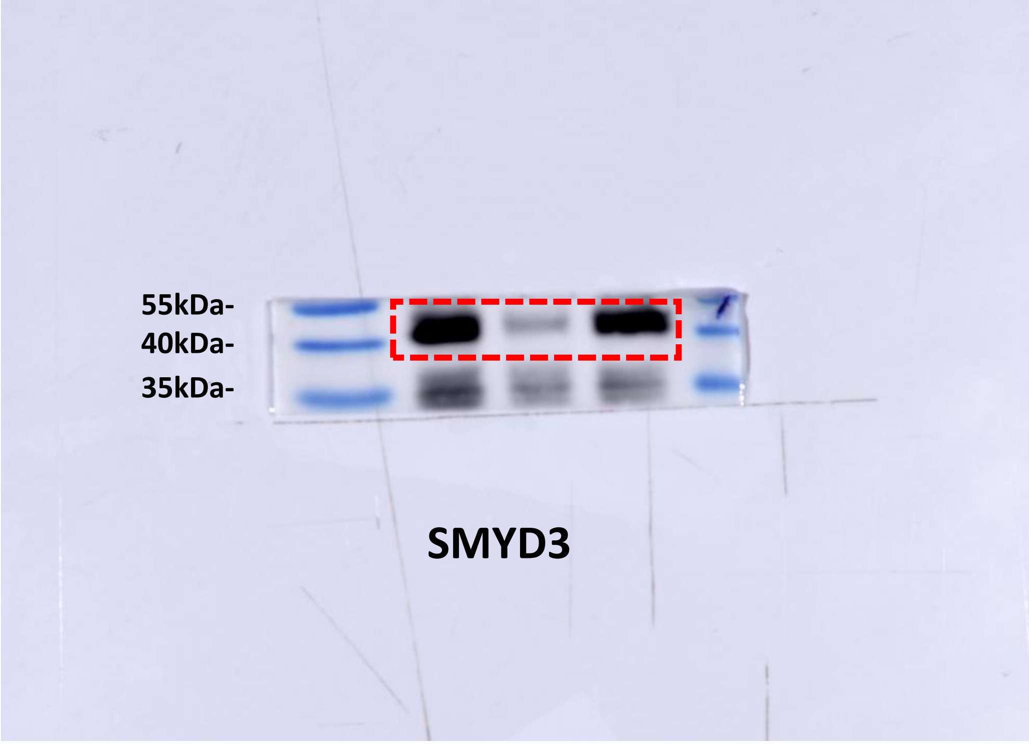

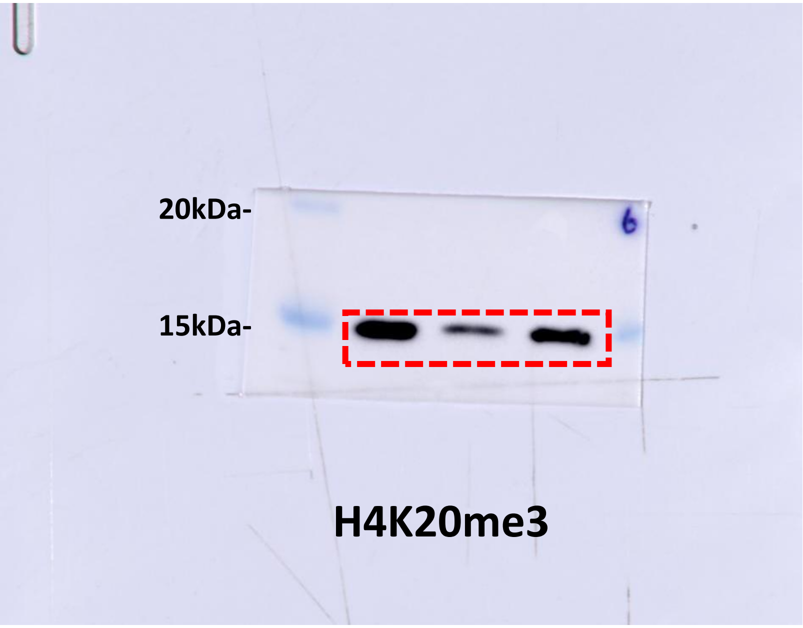

Figure 4E.（HGC-27）

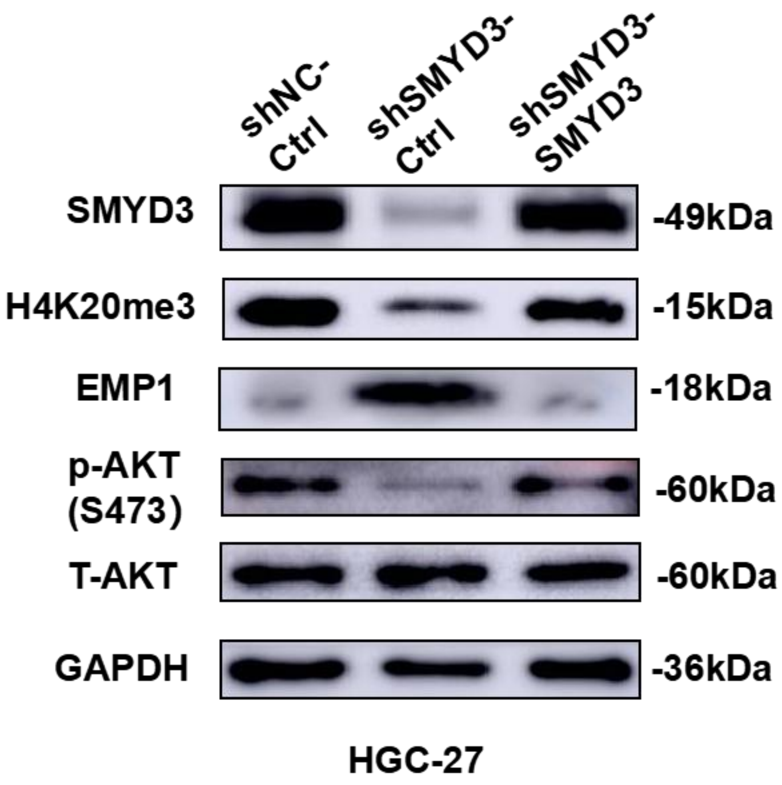

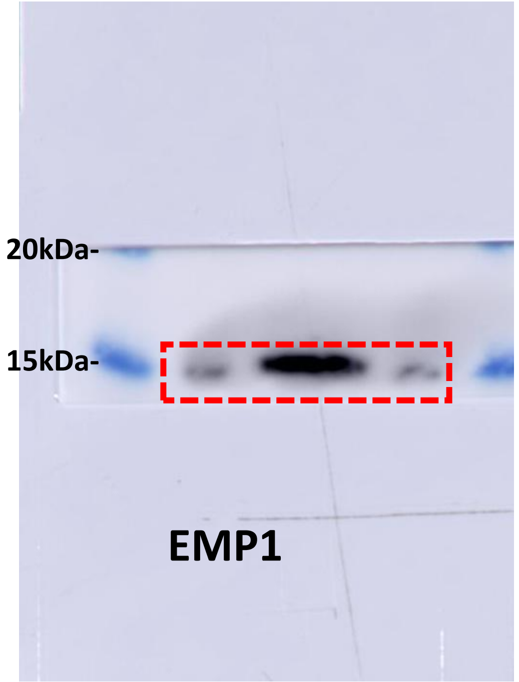

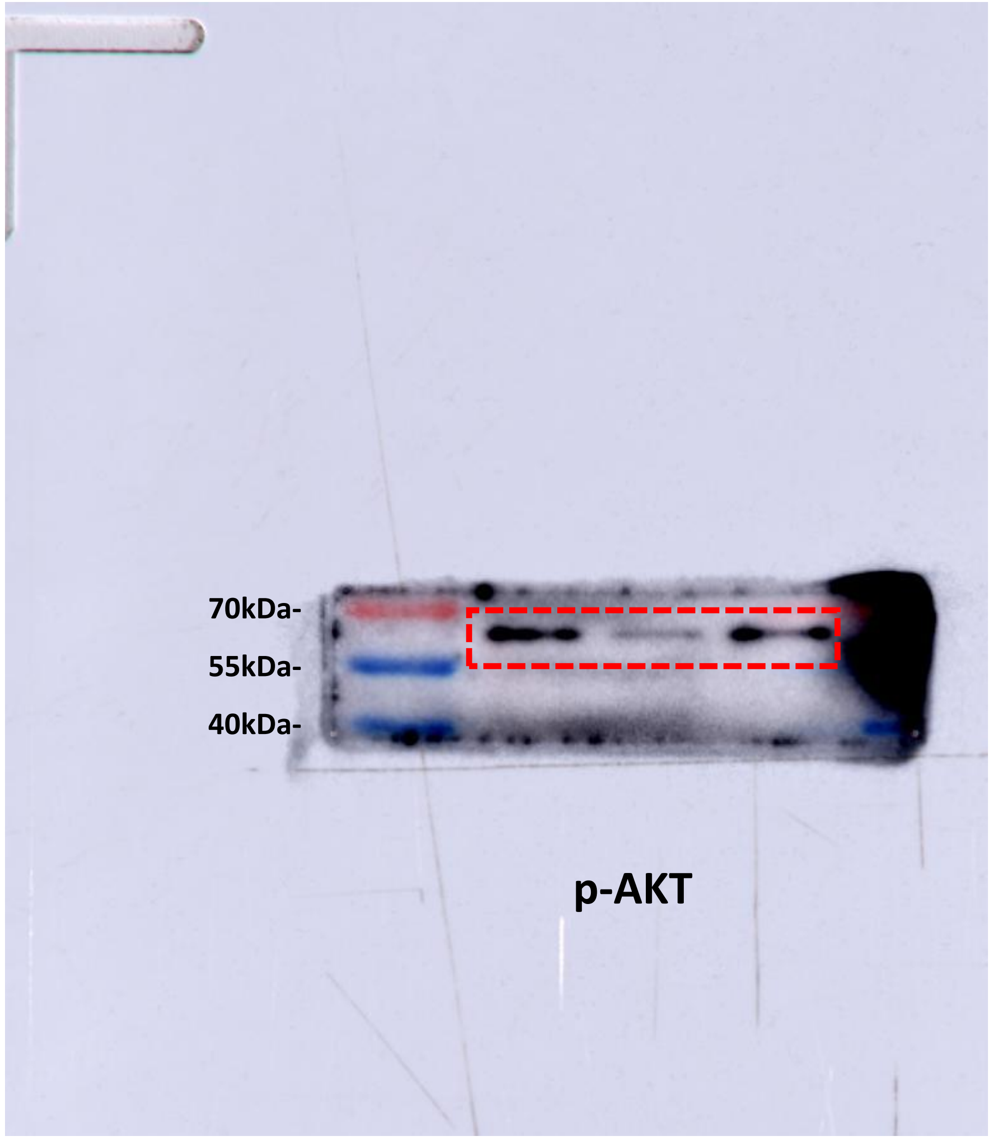

Figure 4E. (HGC-27)

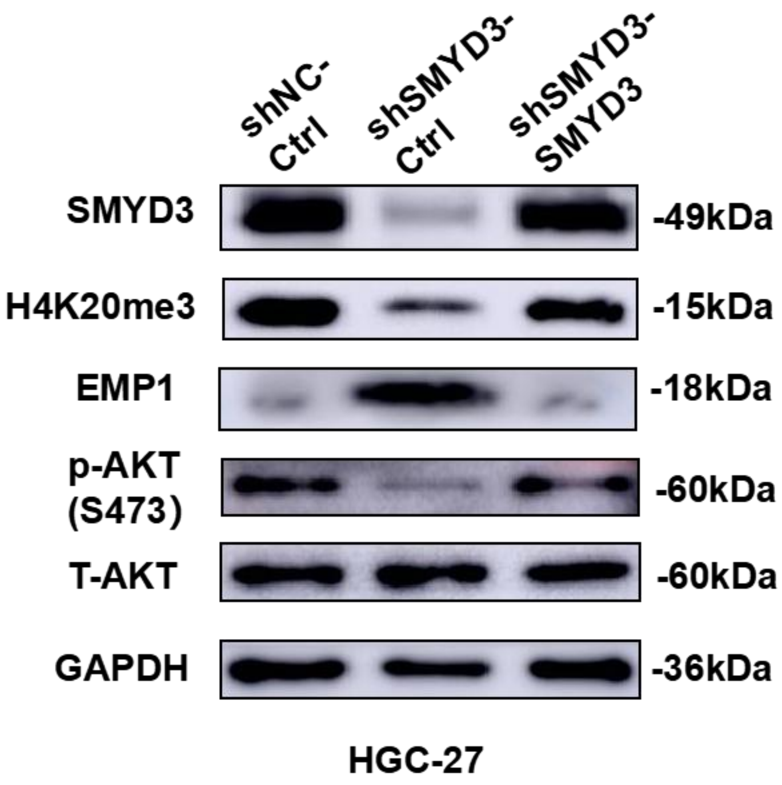

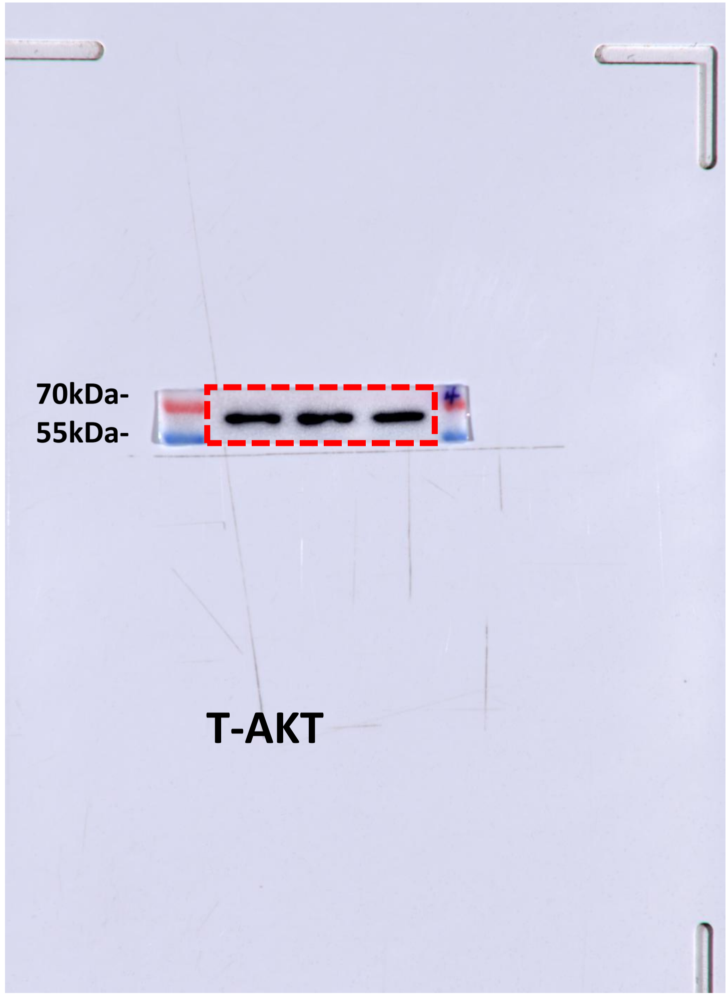

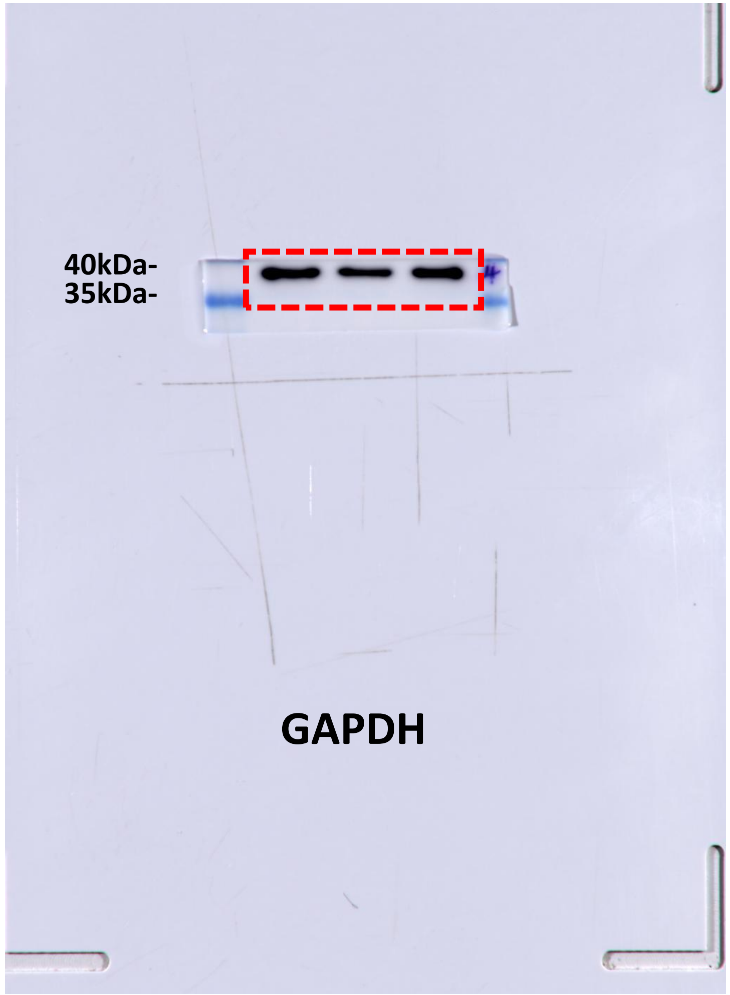

Figure 4E. （SGC-7901）

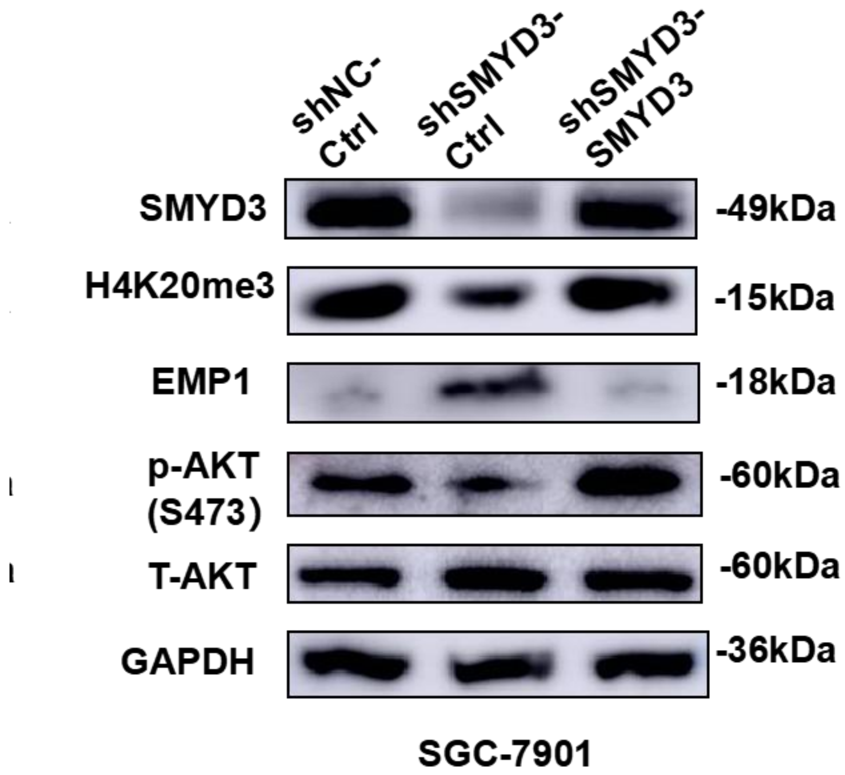

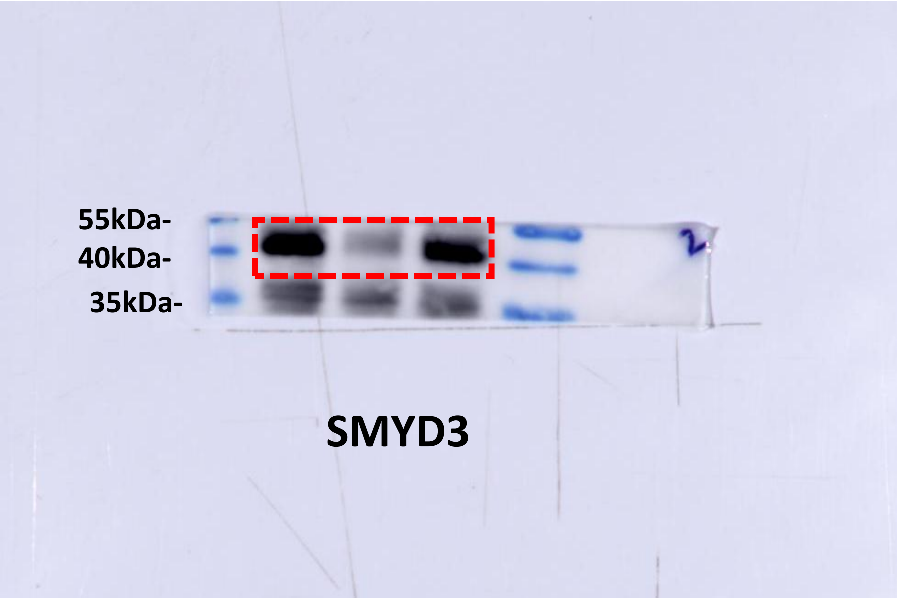

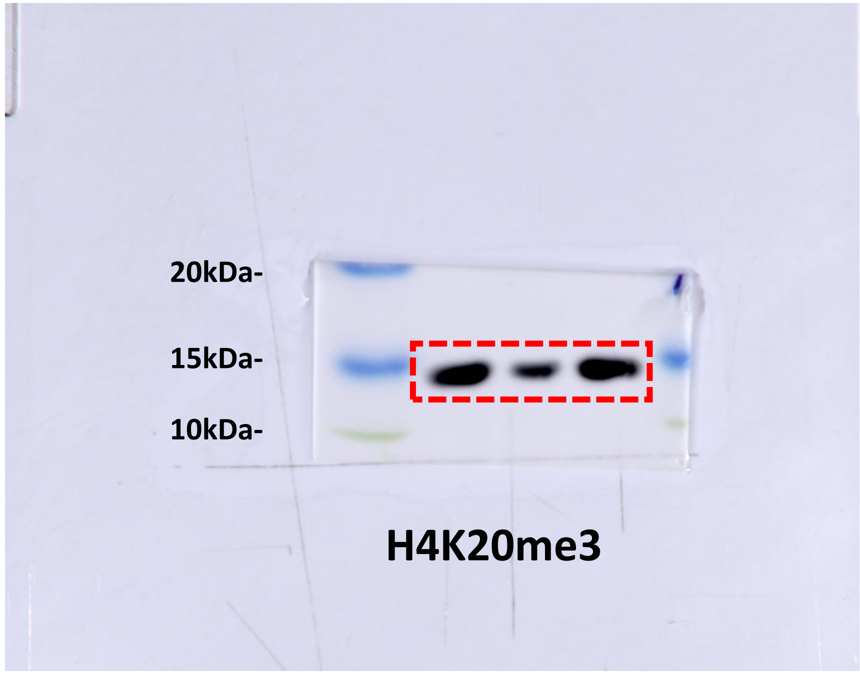

Figure 4E. （SGC-7901）

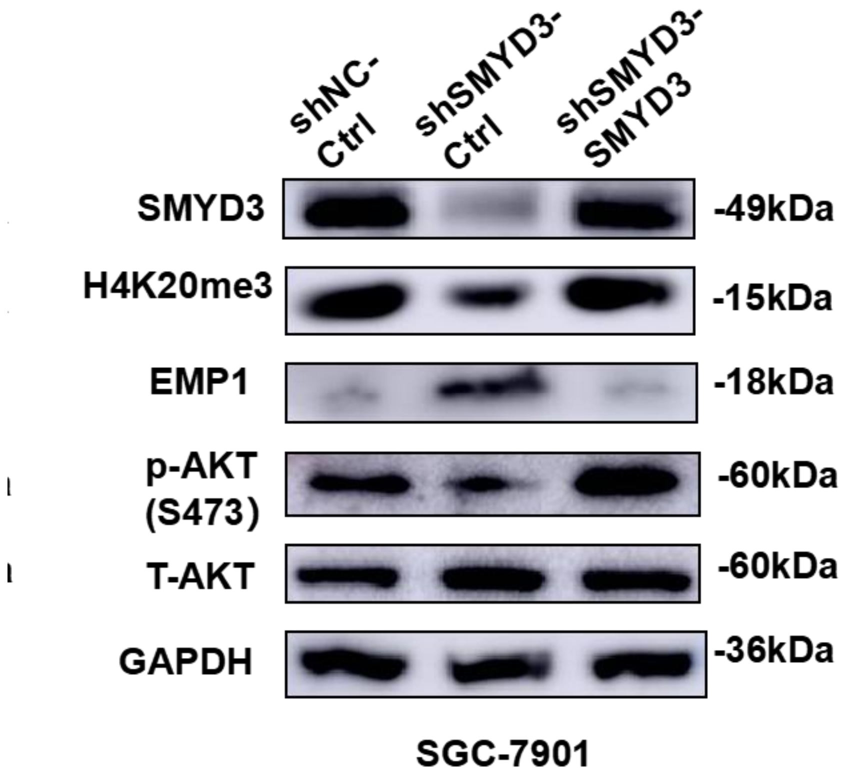

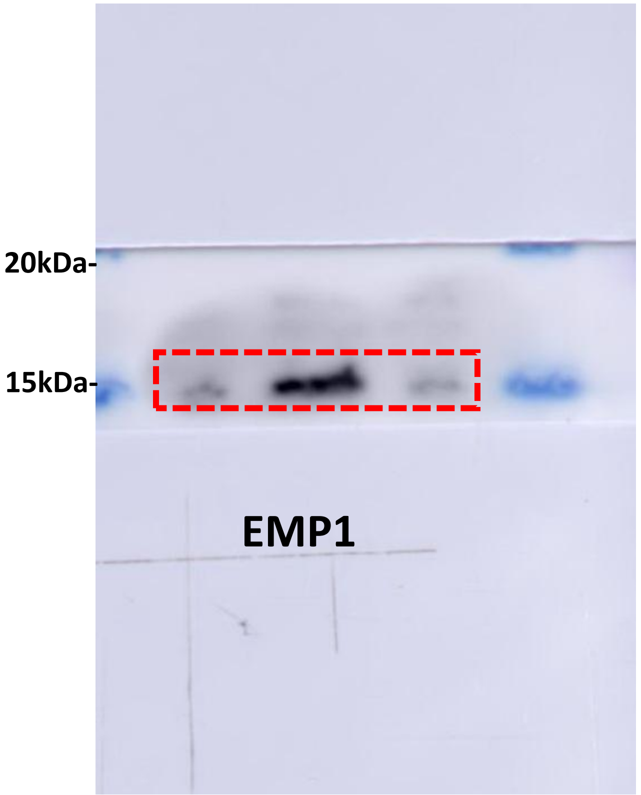

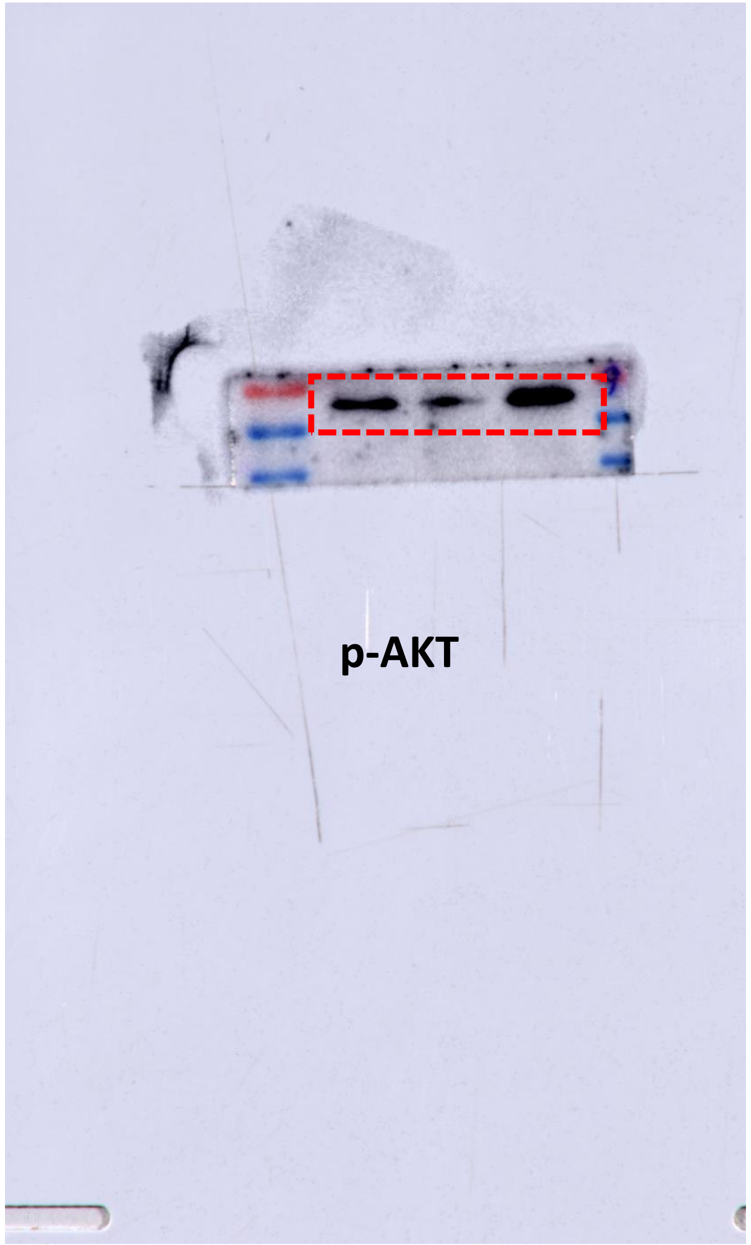

Figure 4E.（SGC-7901）

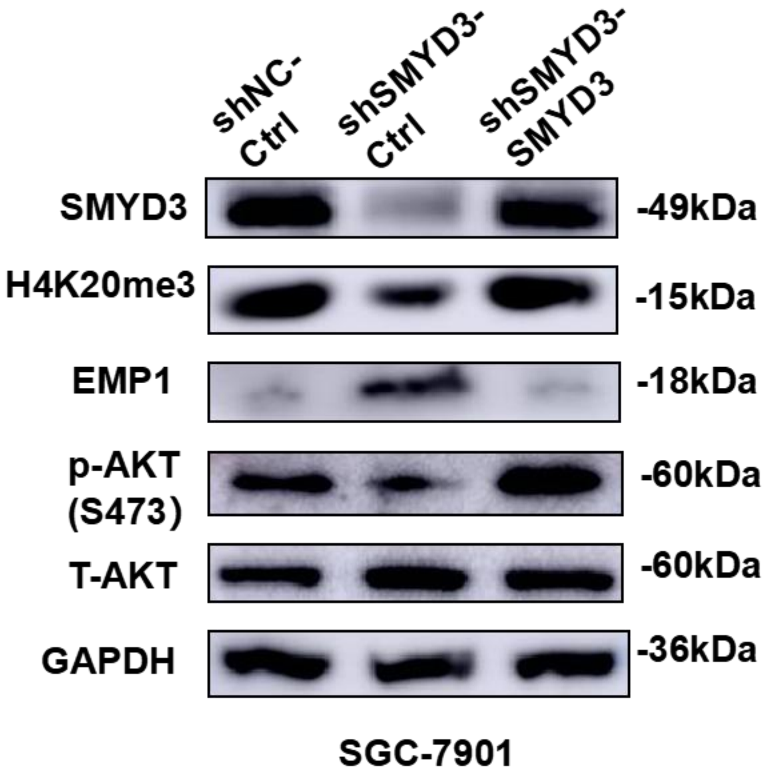

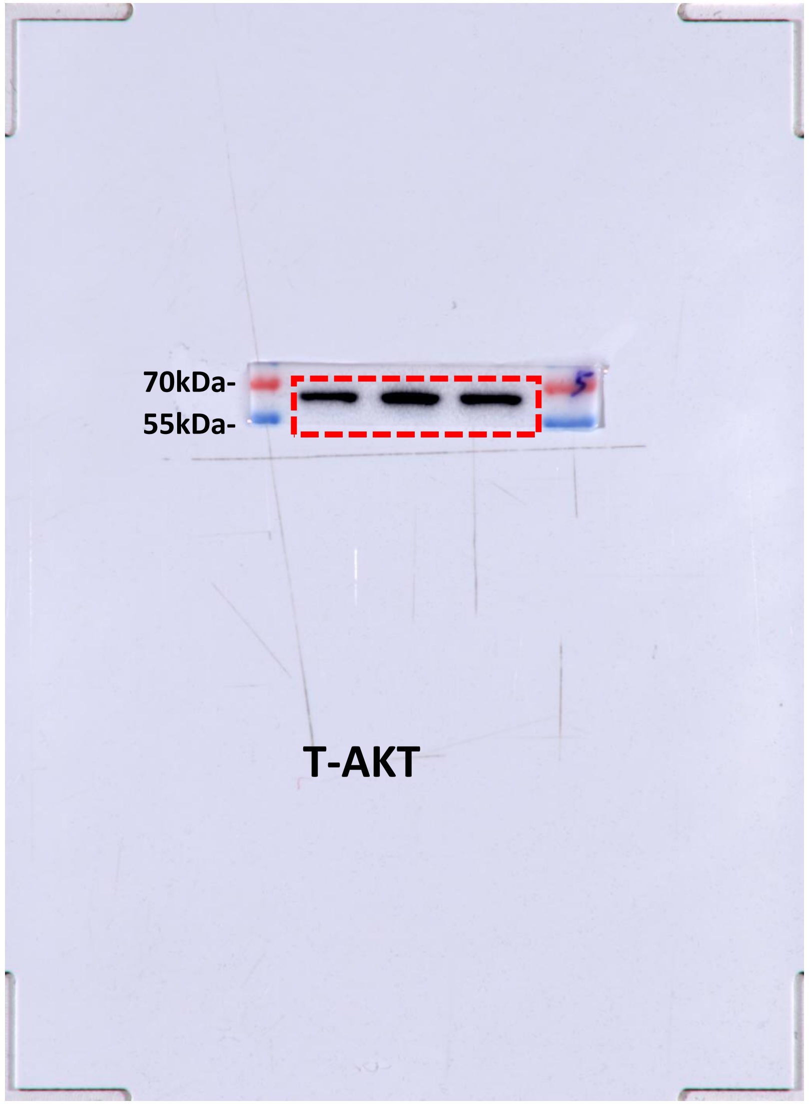

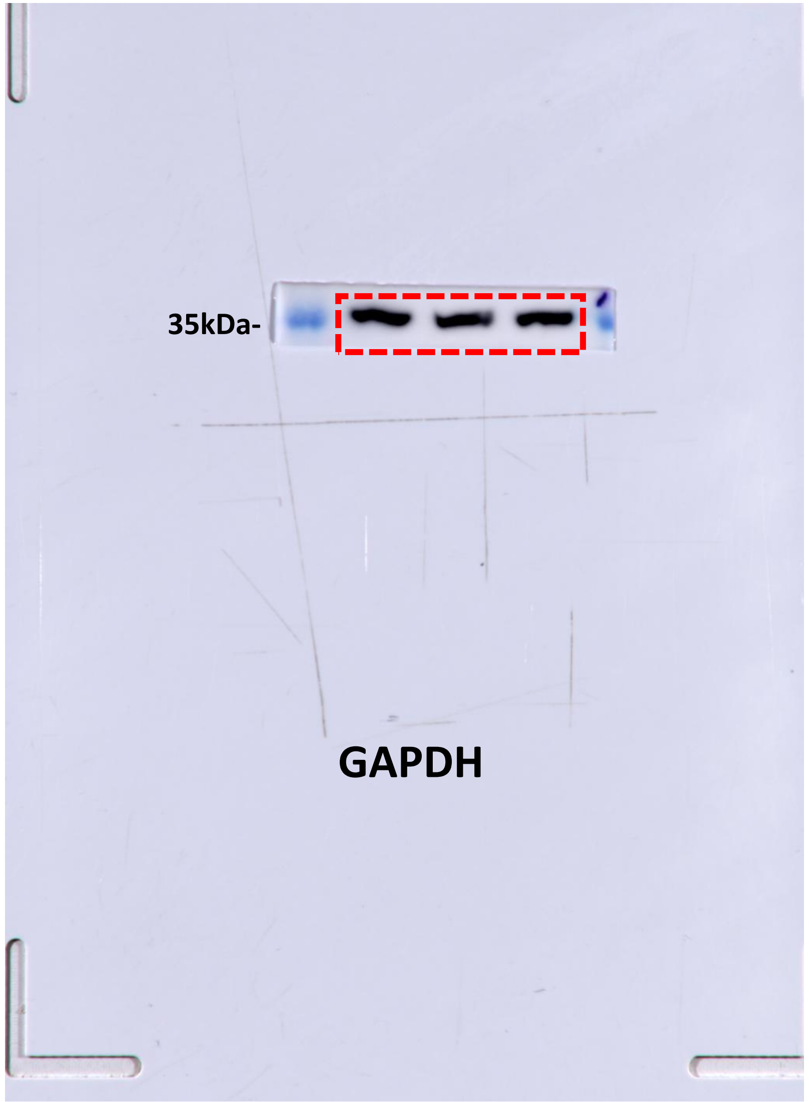

Figure 4F. （HGC-27）

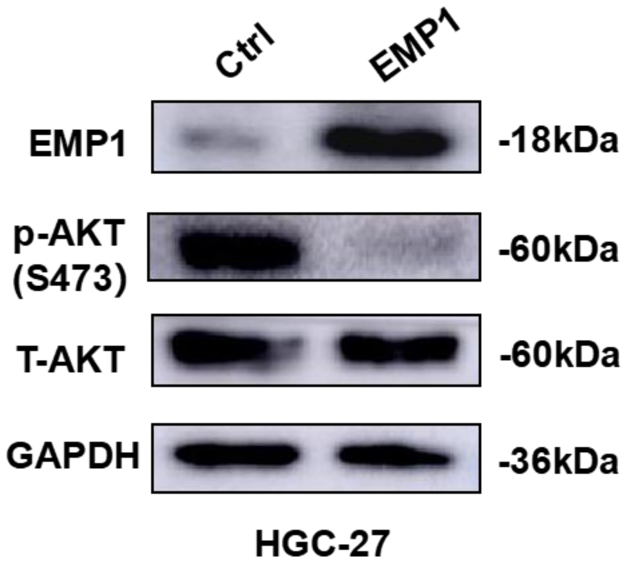

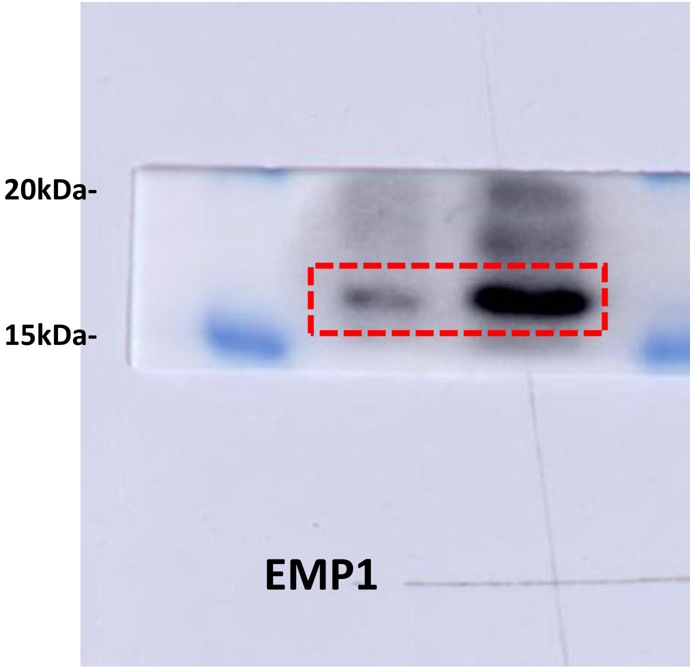

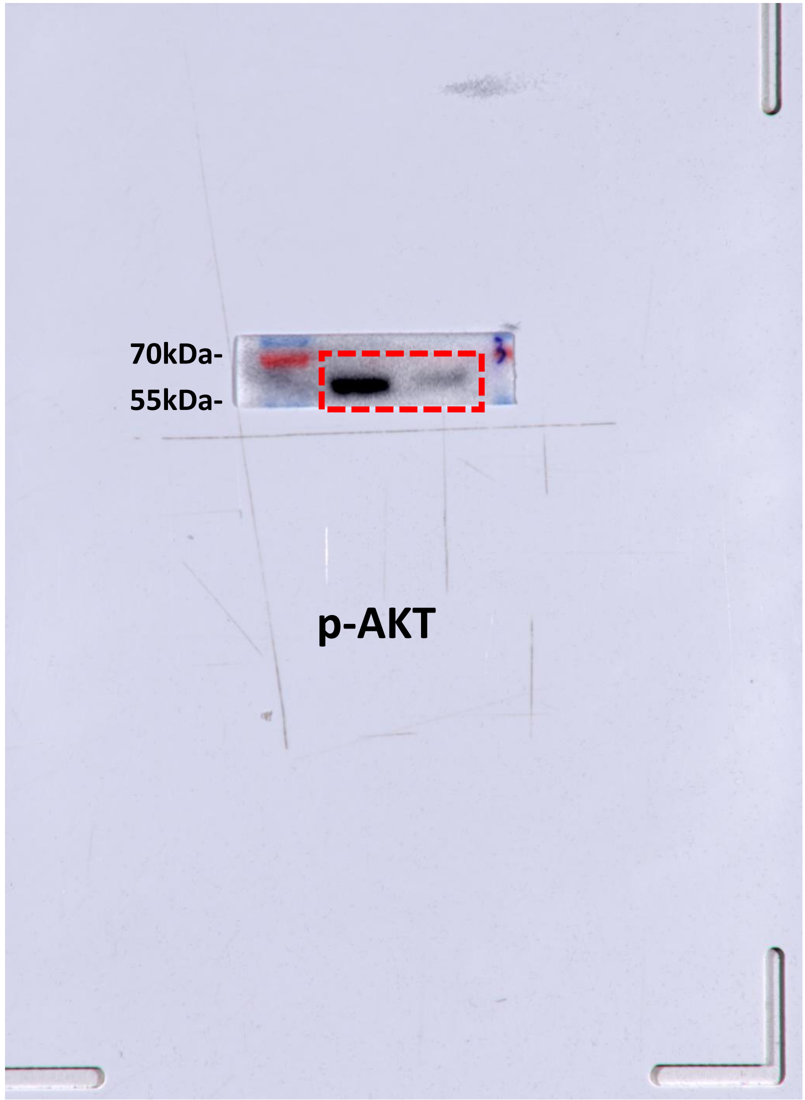

Figure 4F.（HGC-27）

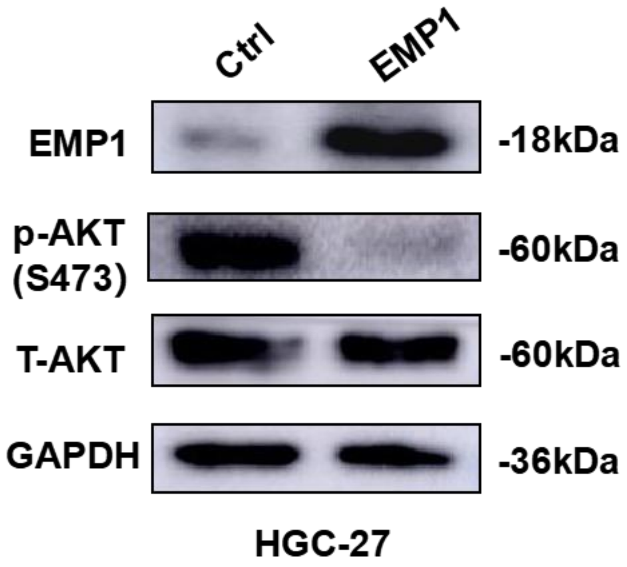

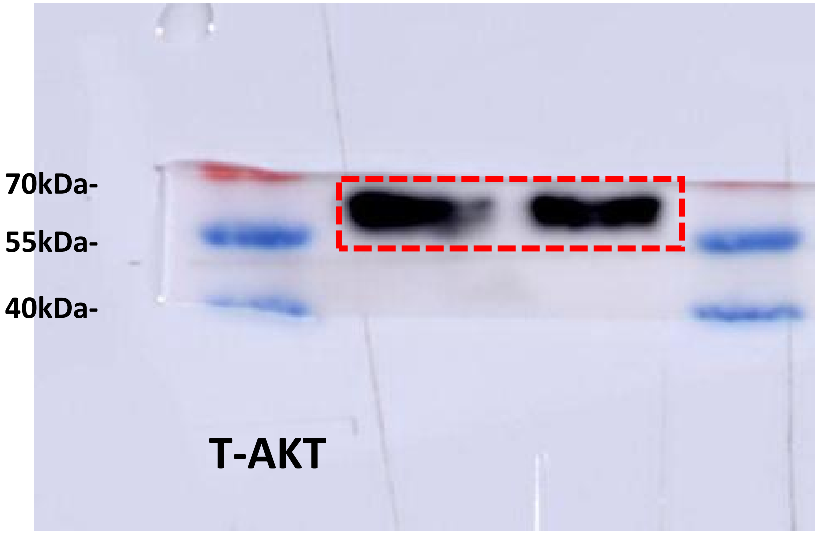

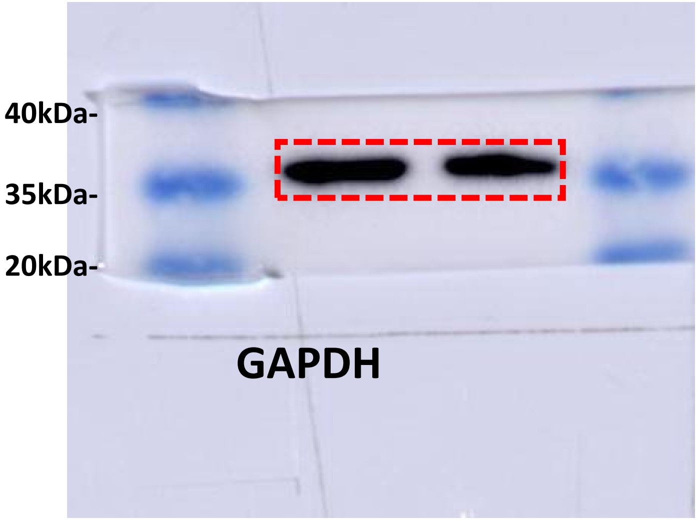

Figure 4F. （SGC-7901）

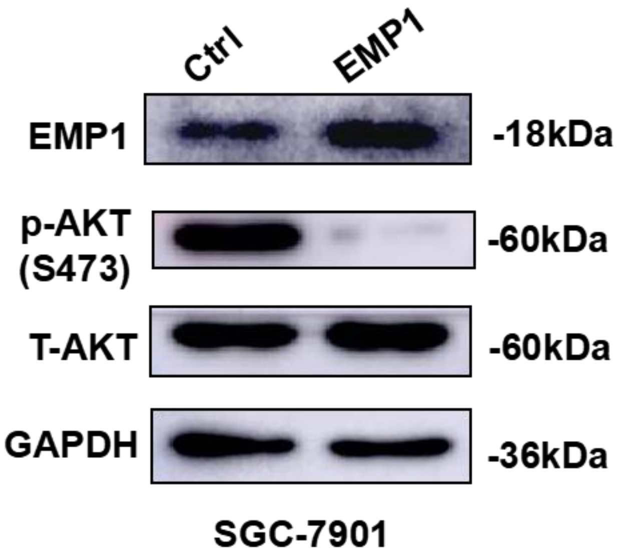

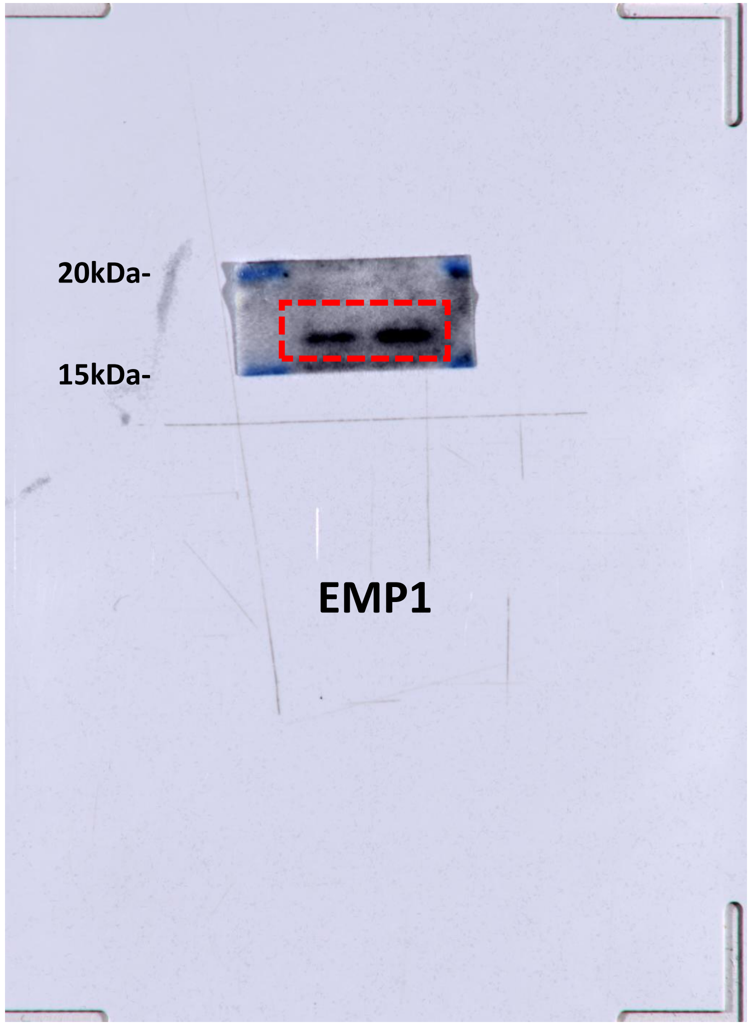

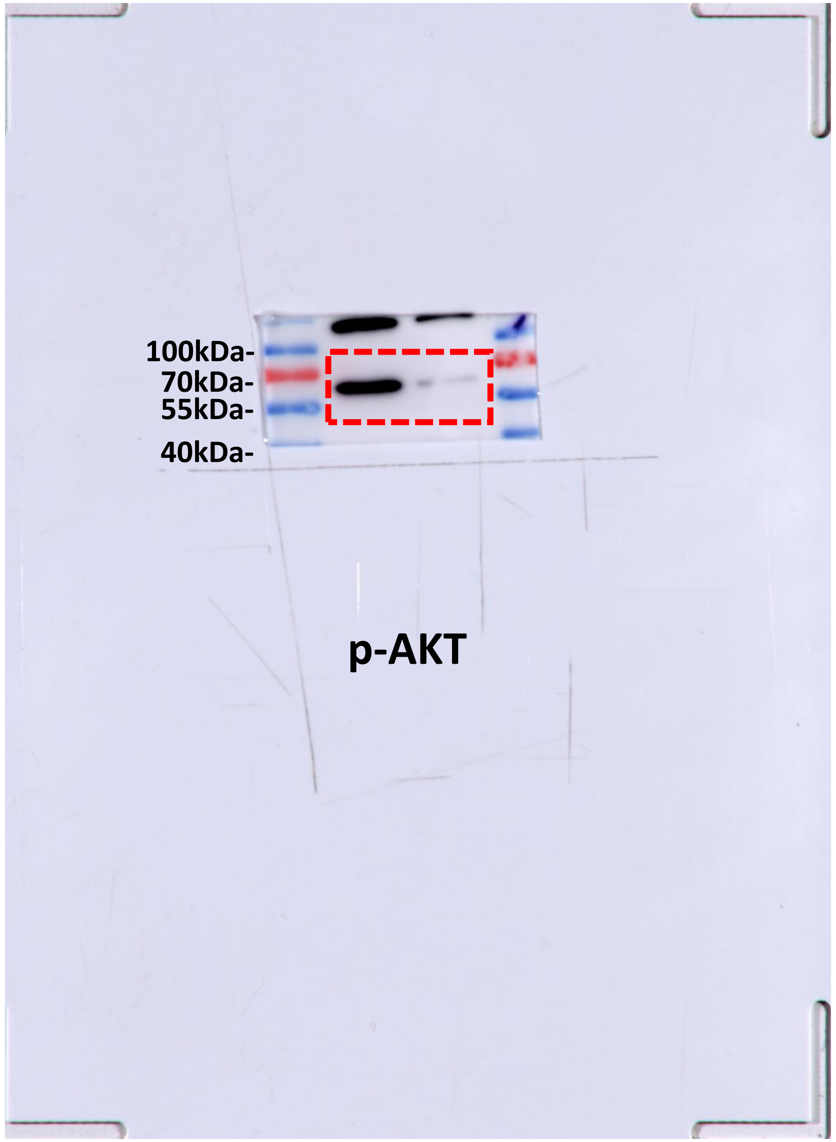

Figure 4F.（SGC-7901）

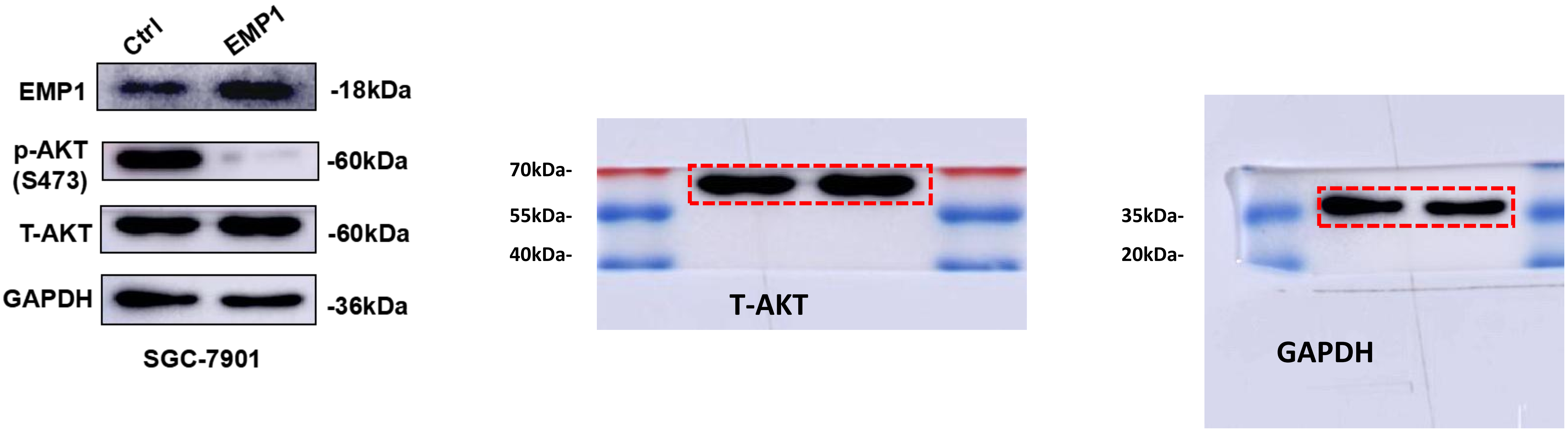

Figure 5A.（HGC-27）

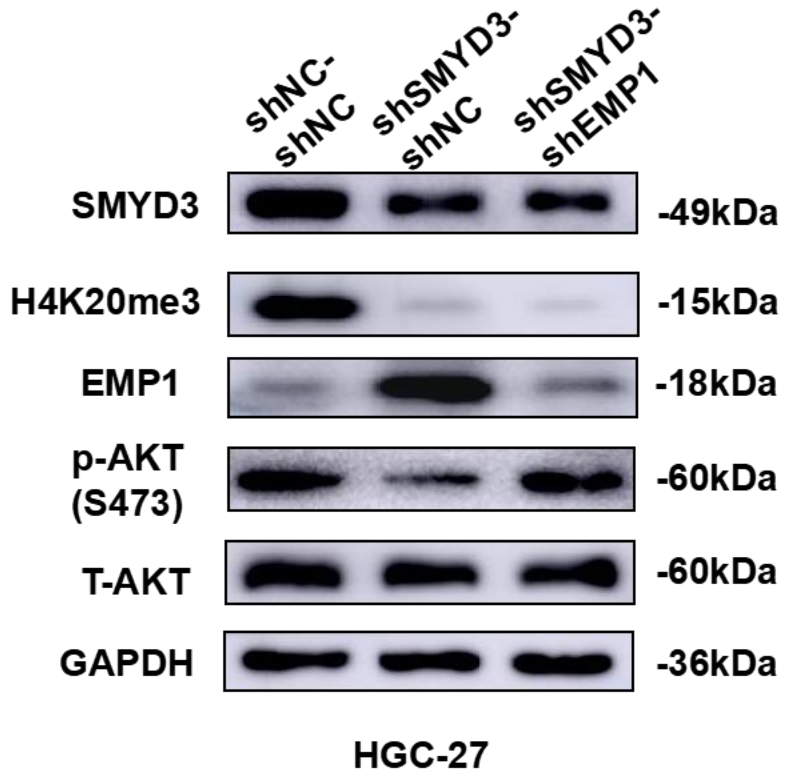

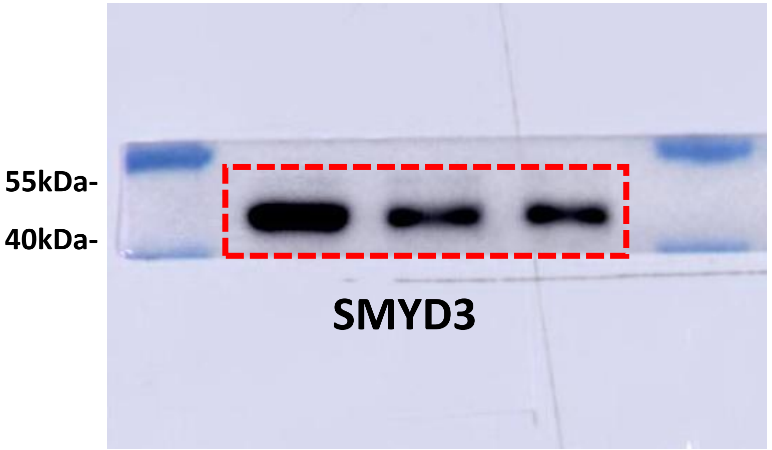

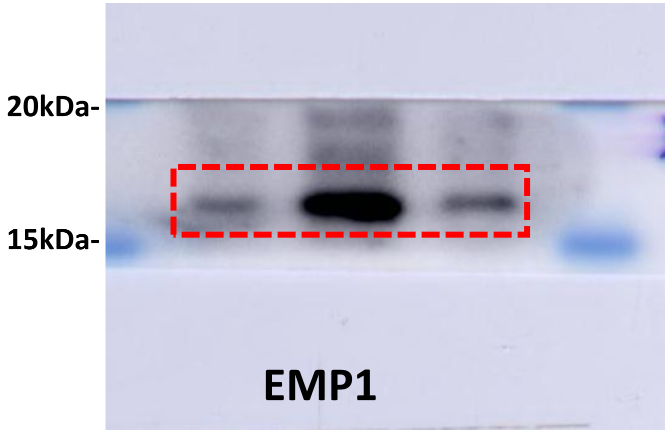

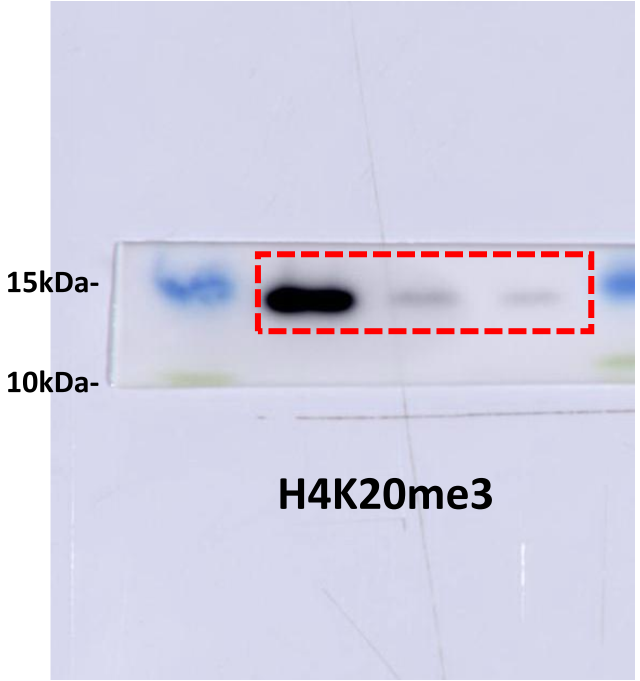

Figure 5A.（HGC-27）

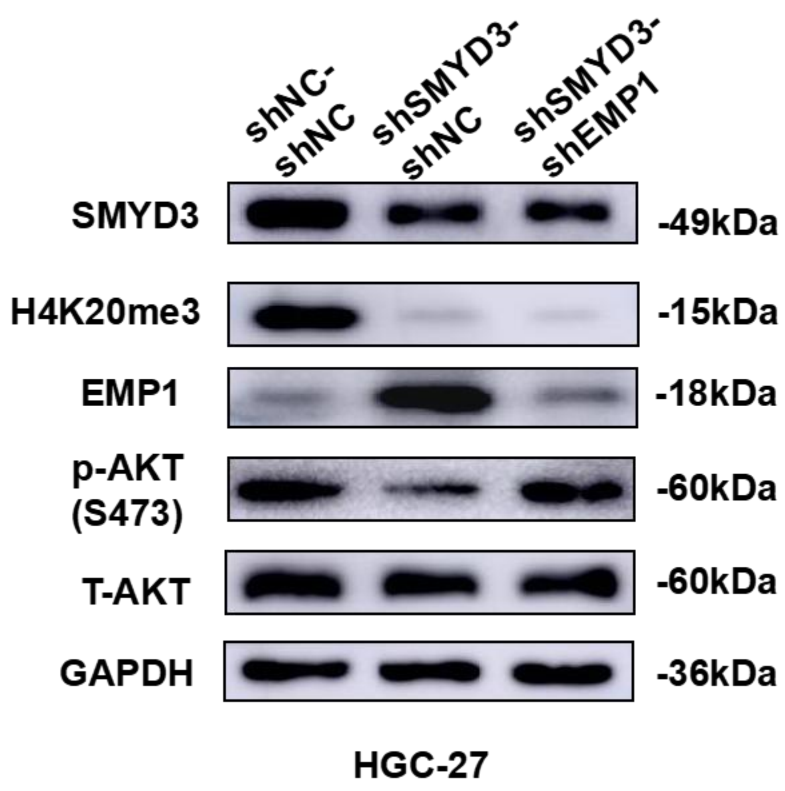

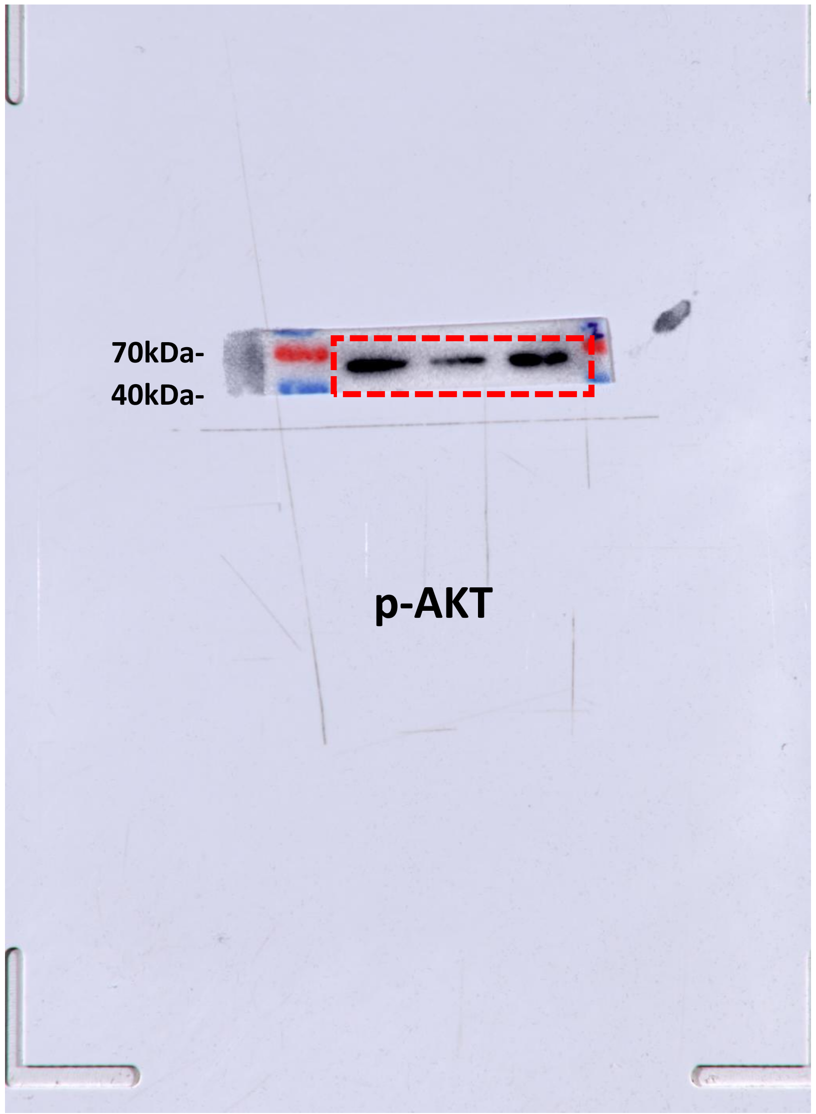

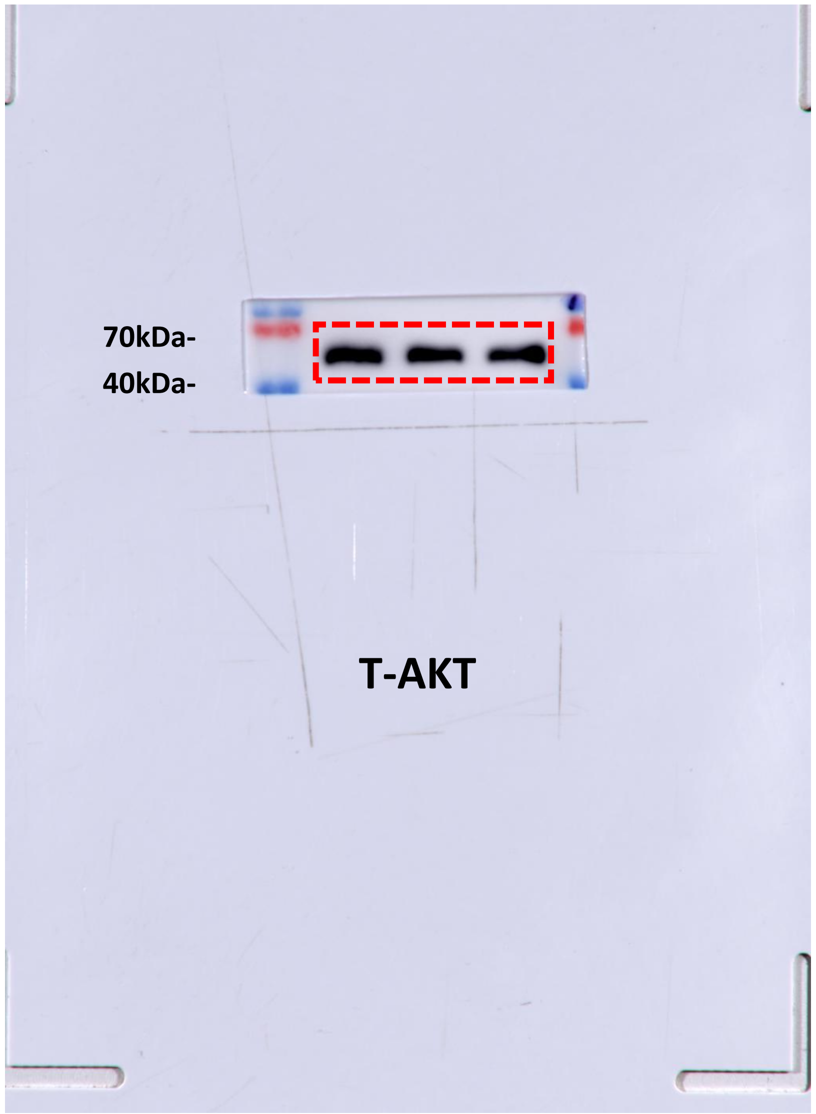

Figure 5A.（HGC-27）

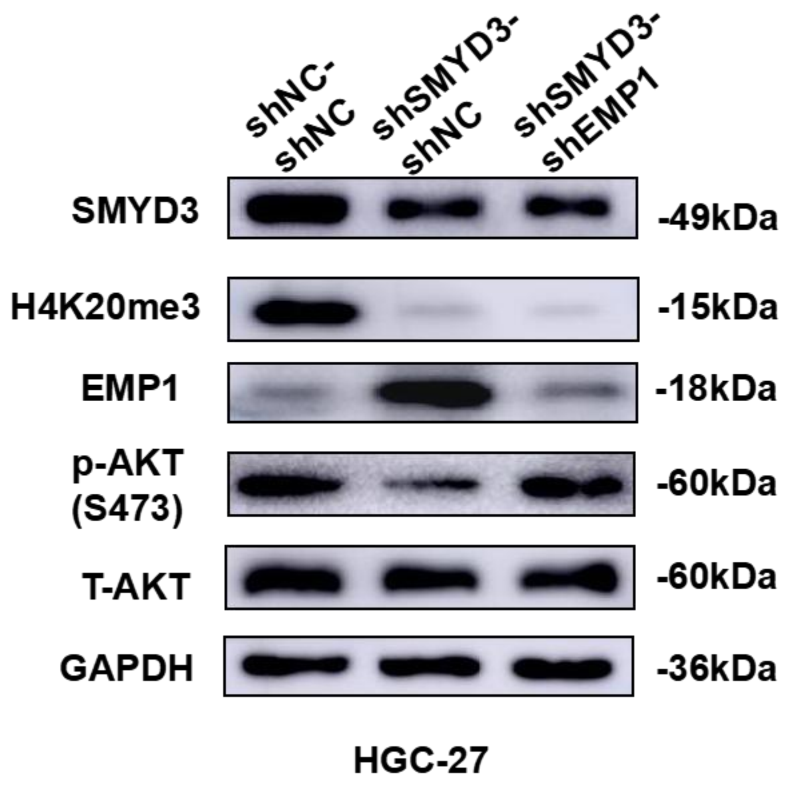

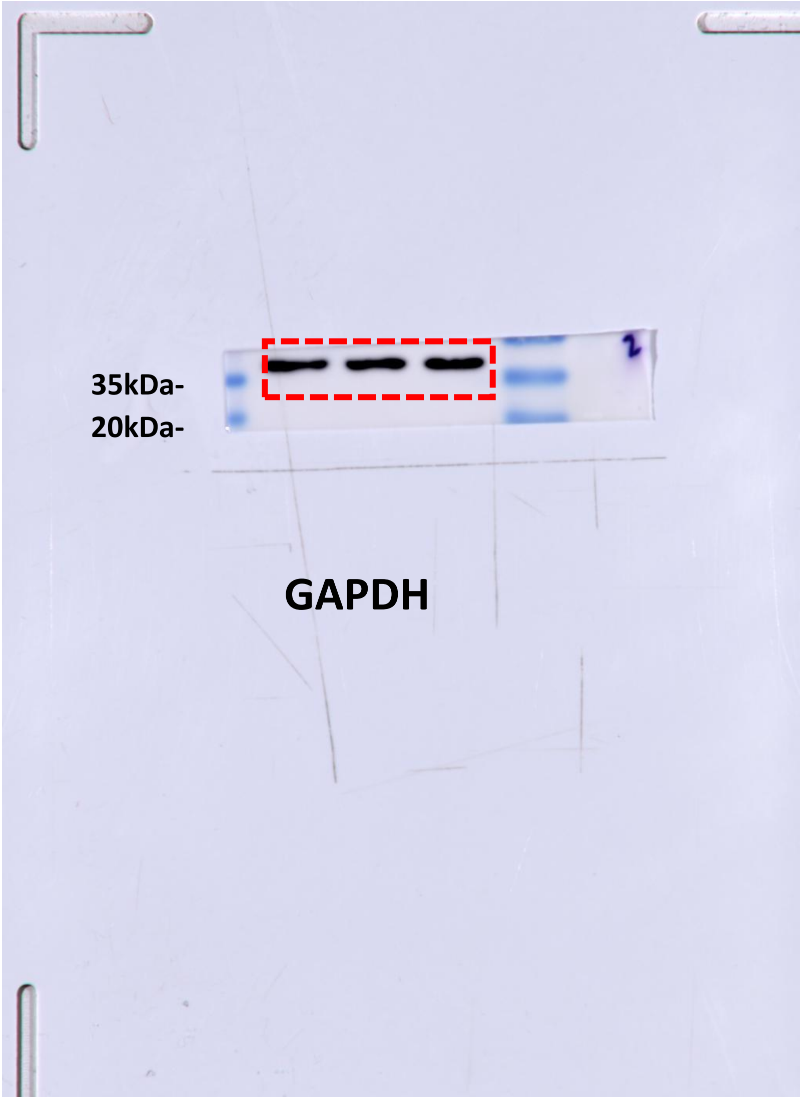

Figure 5A. （SGC-7901）

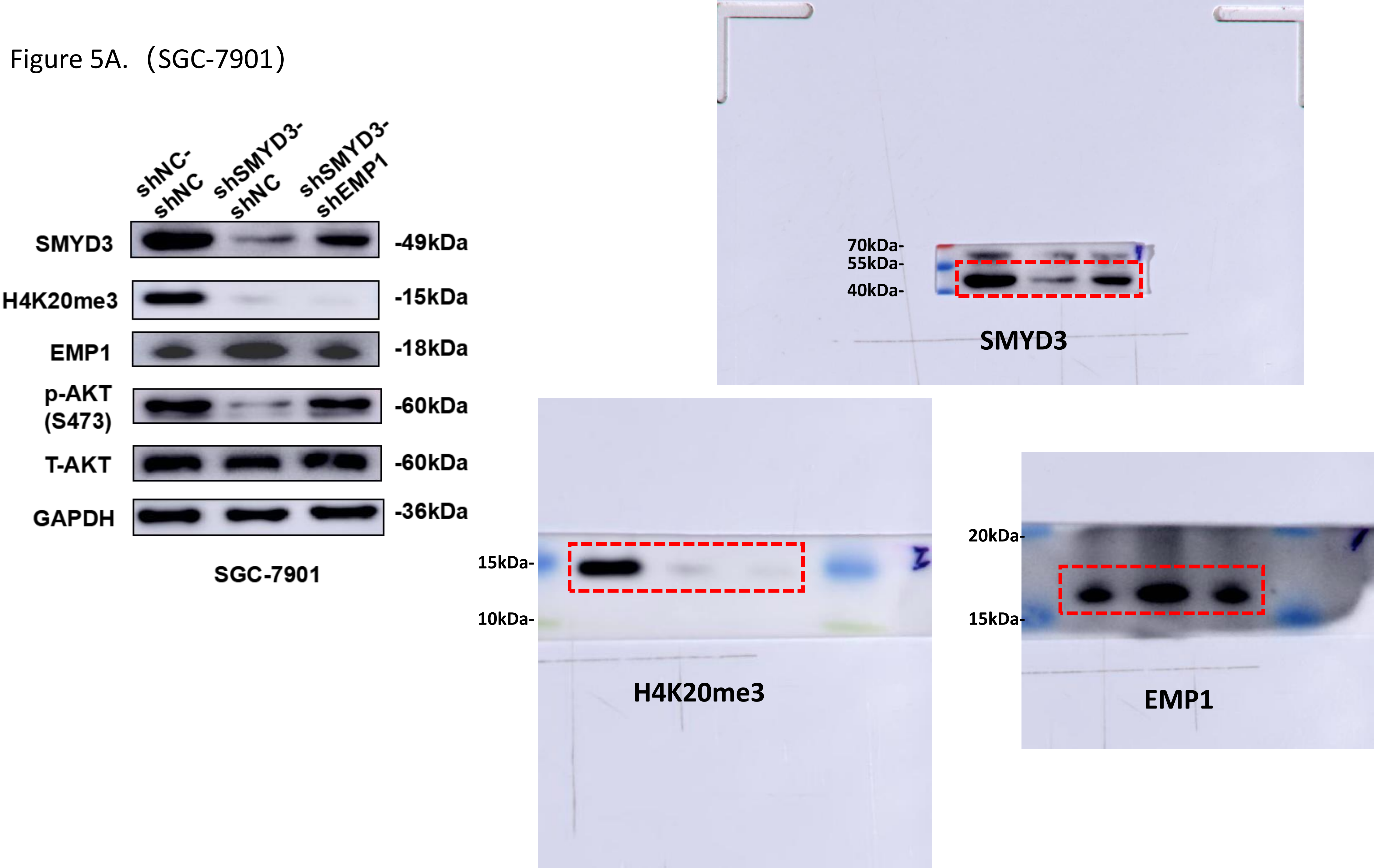

Figure 5A. （SGC-7901）

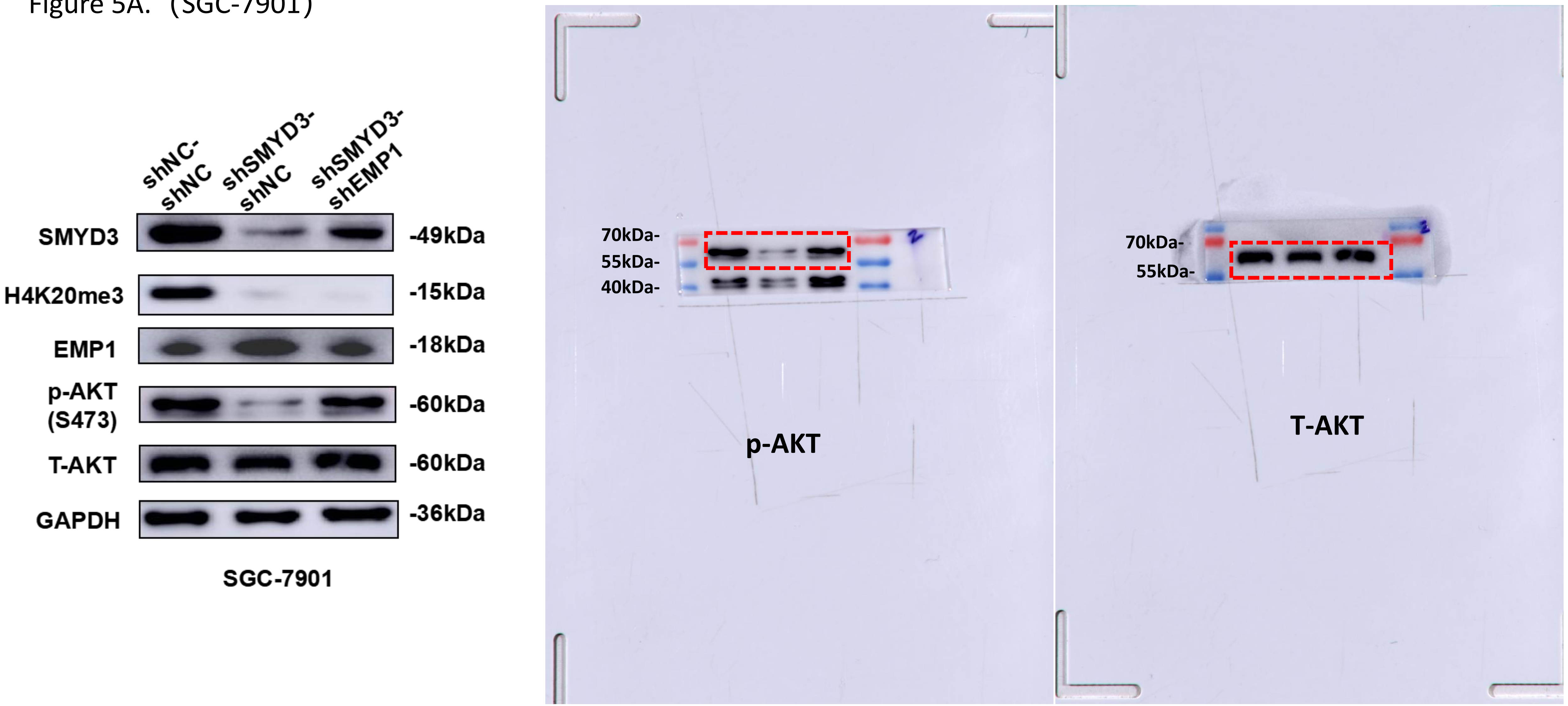

Figure 5A.（SGC-7901）

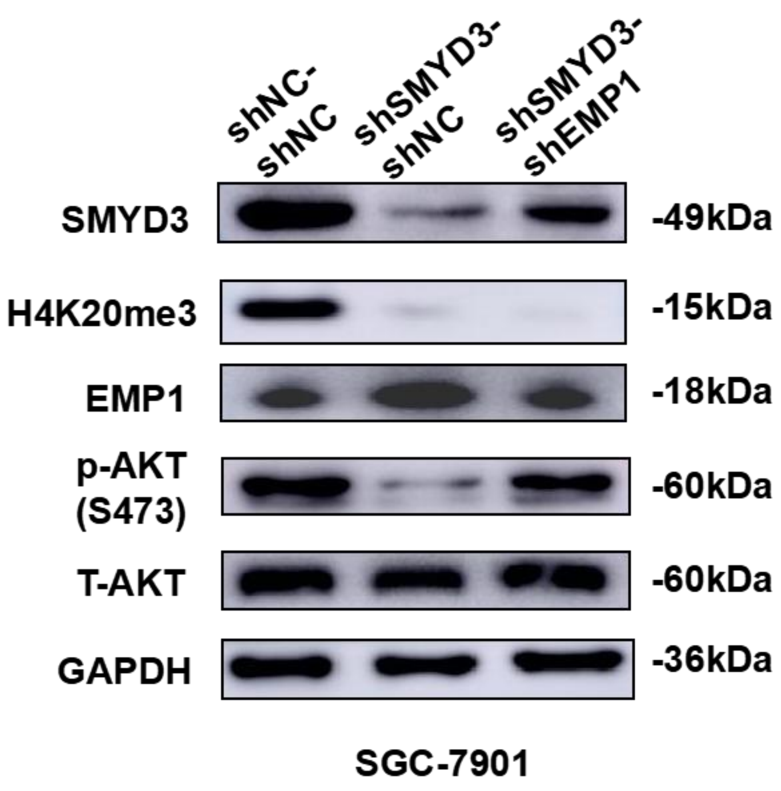

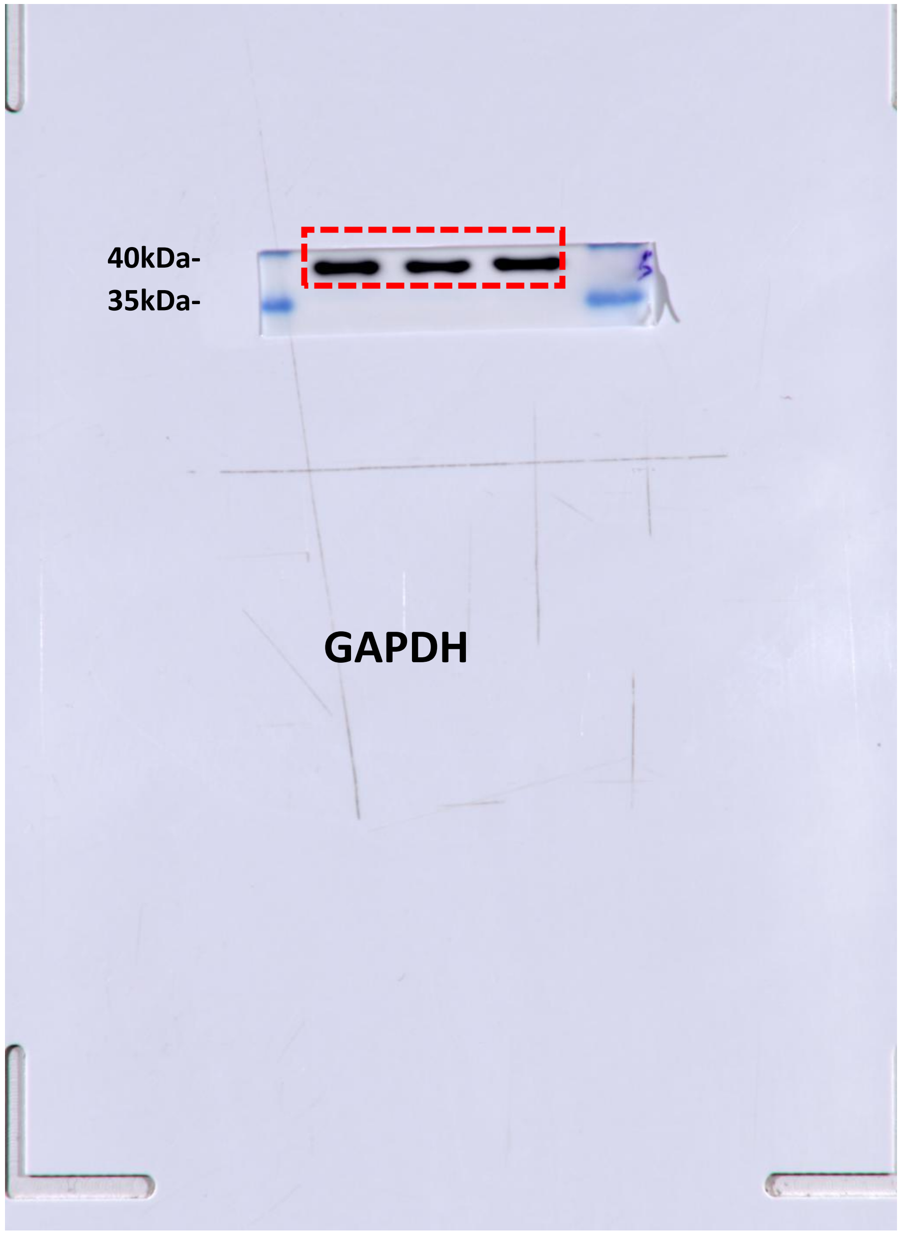

Figure 6A.（HGC-27）

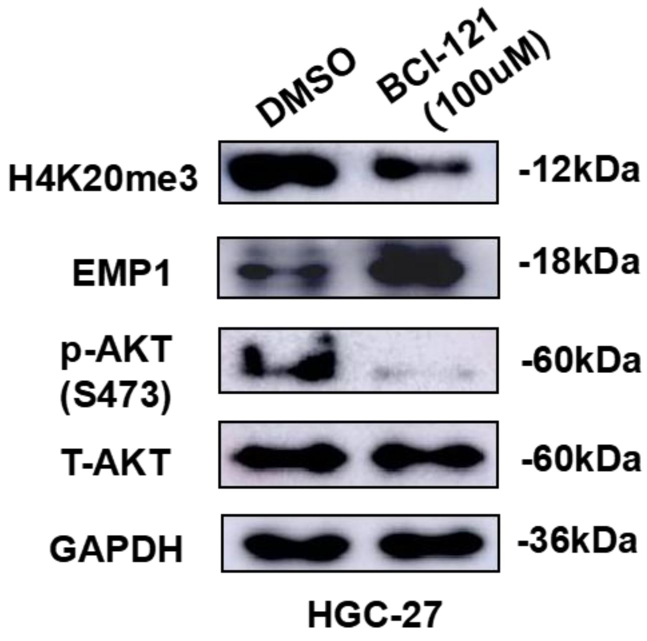

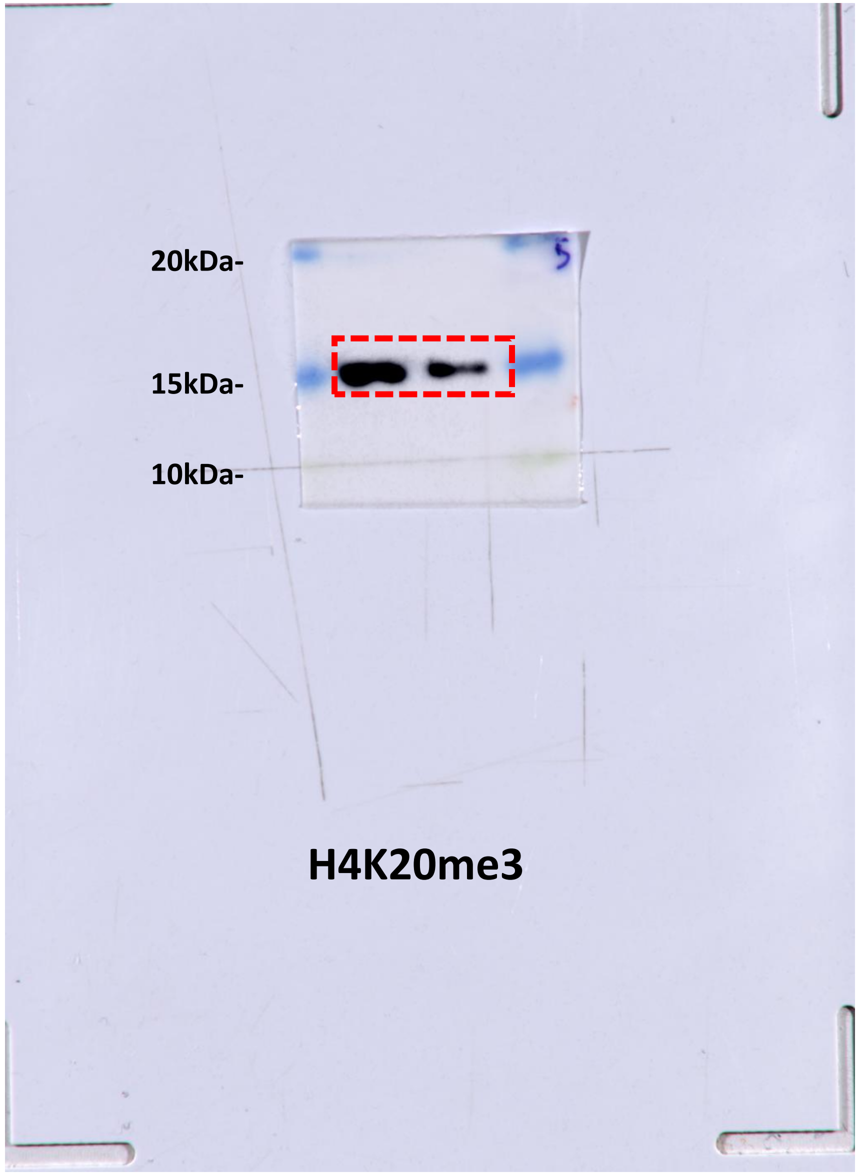

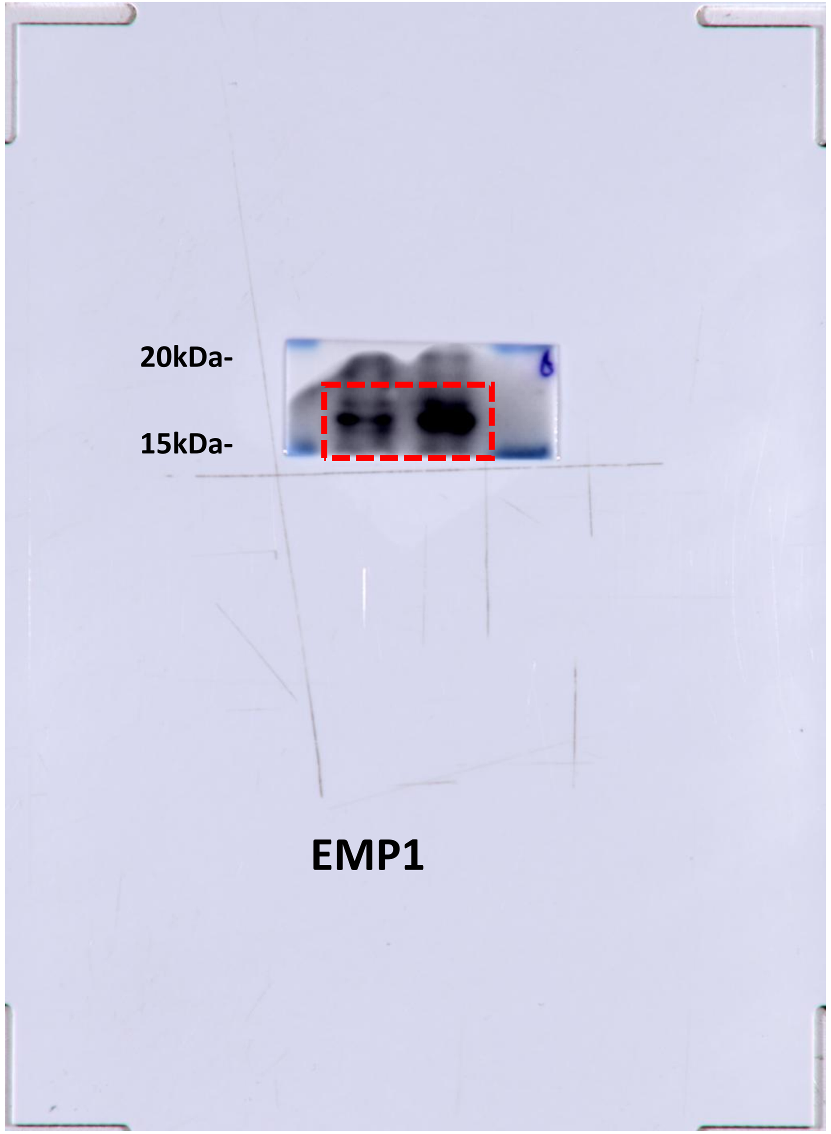

Figure 6A.（HGC-27）

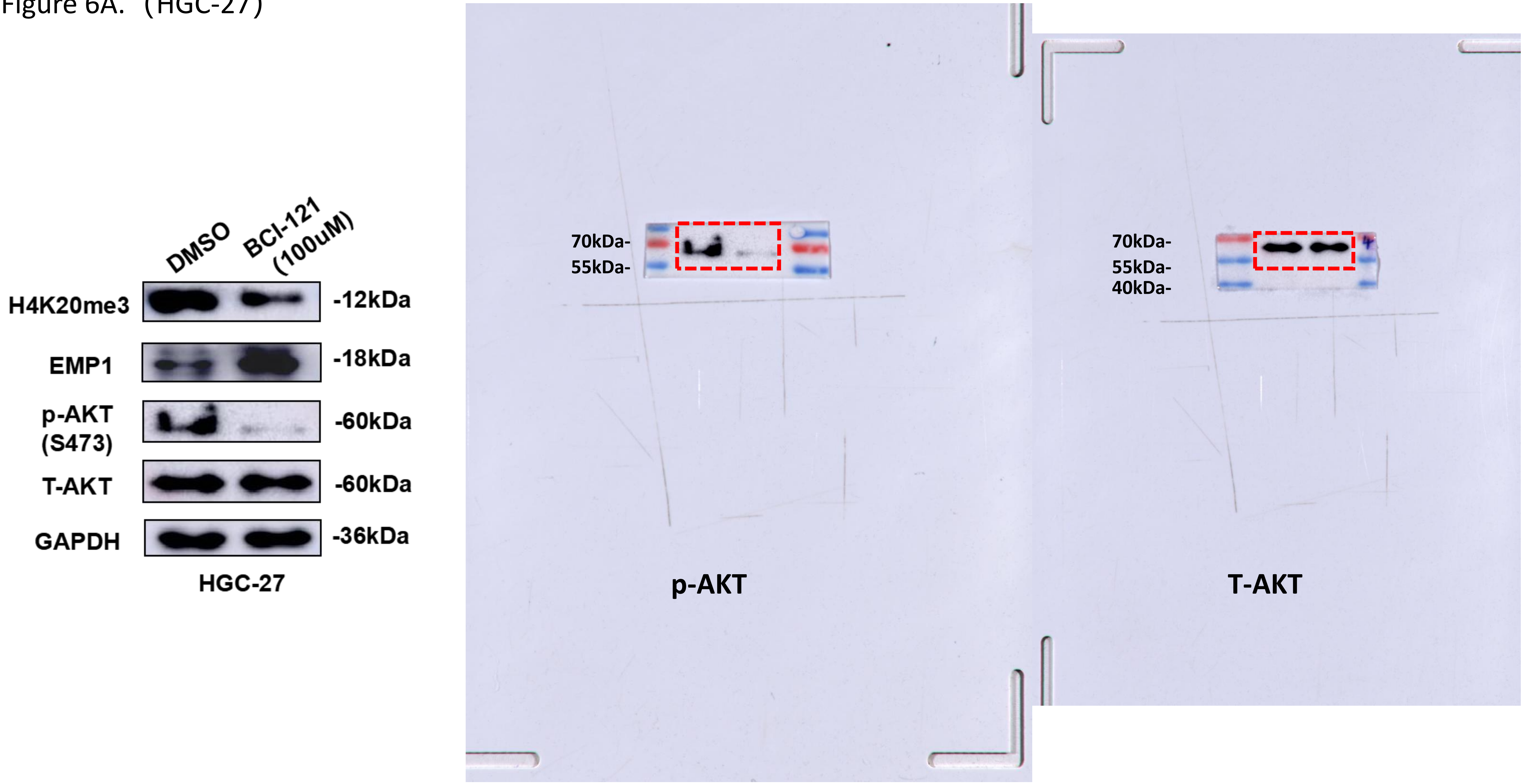

Figure 6A. （HGC-27）

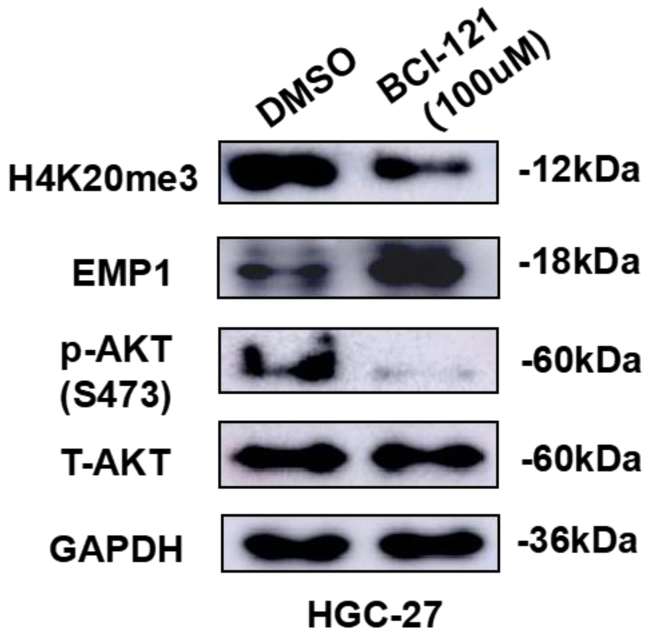

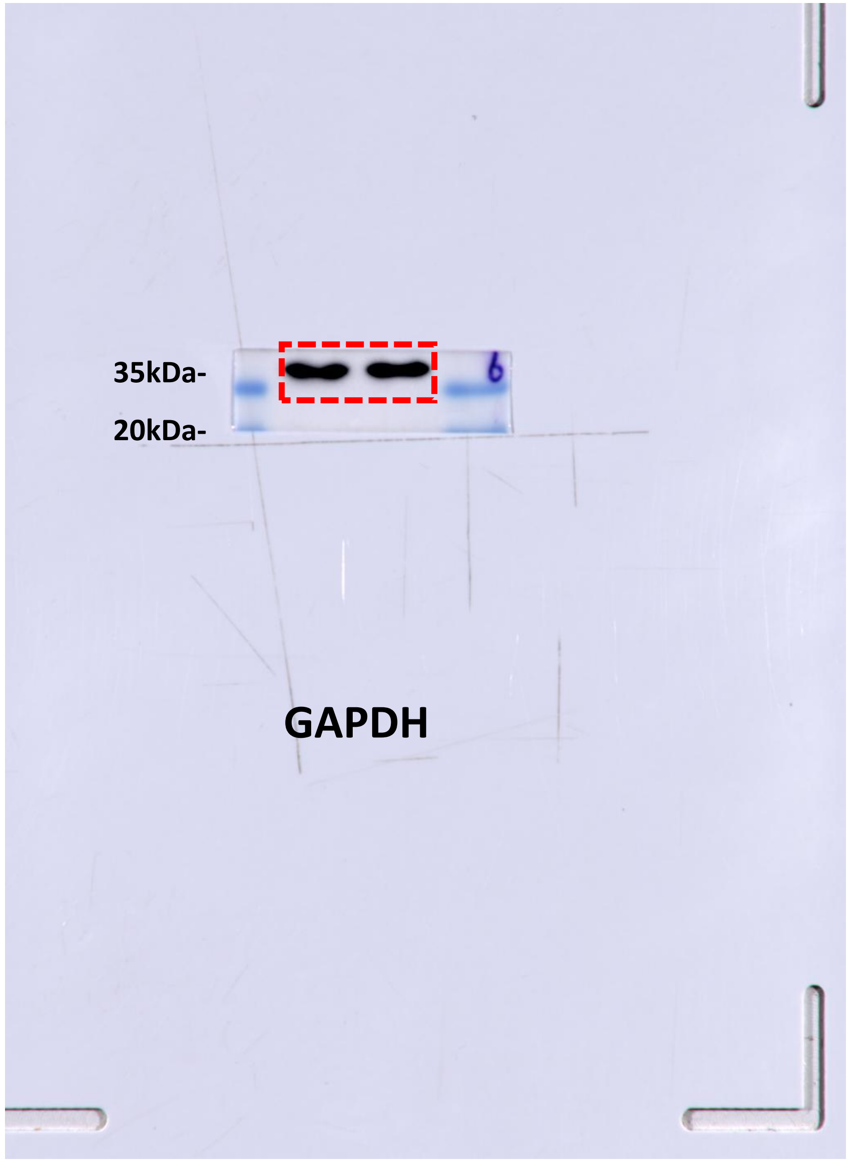

Figure 6A. （SGC-7901）

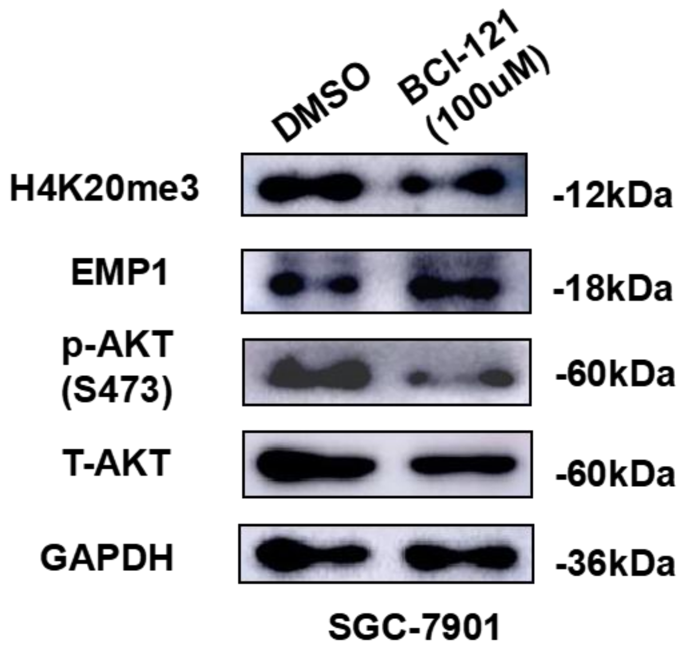

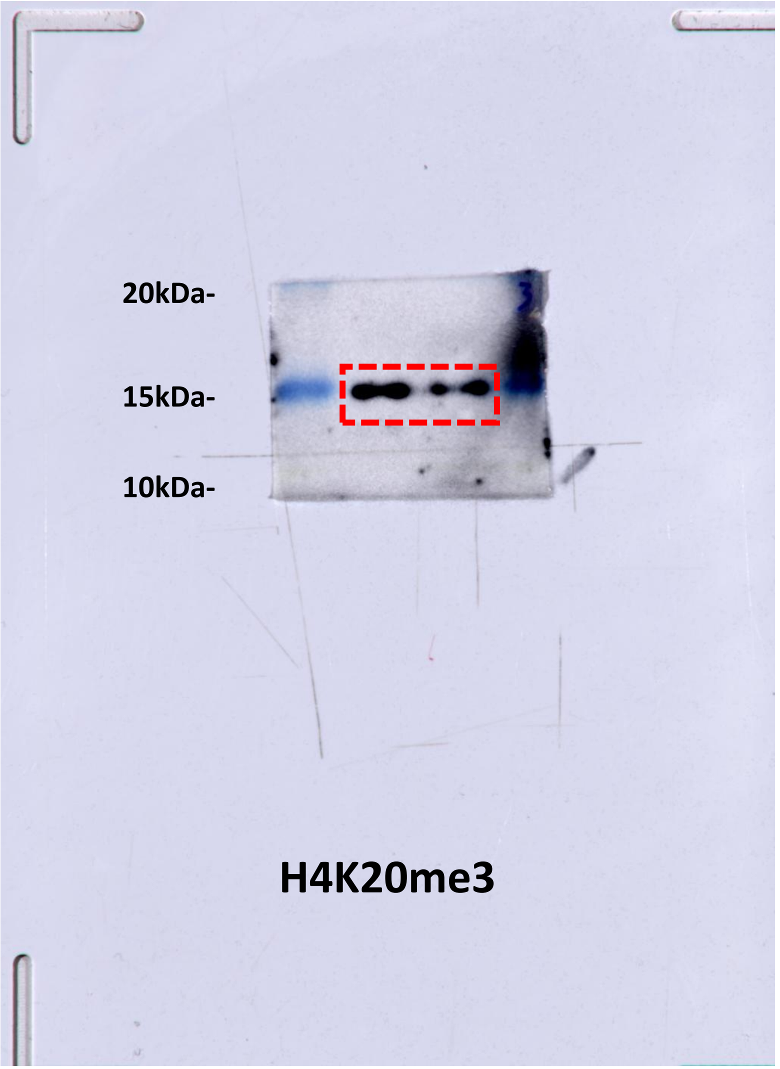

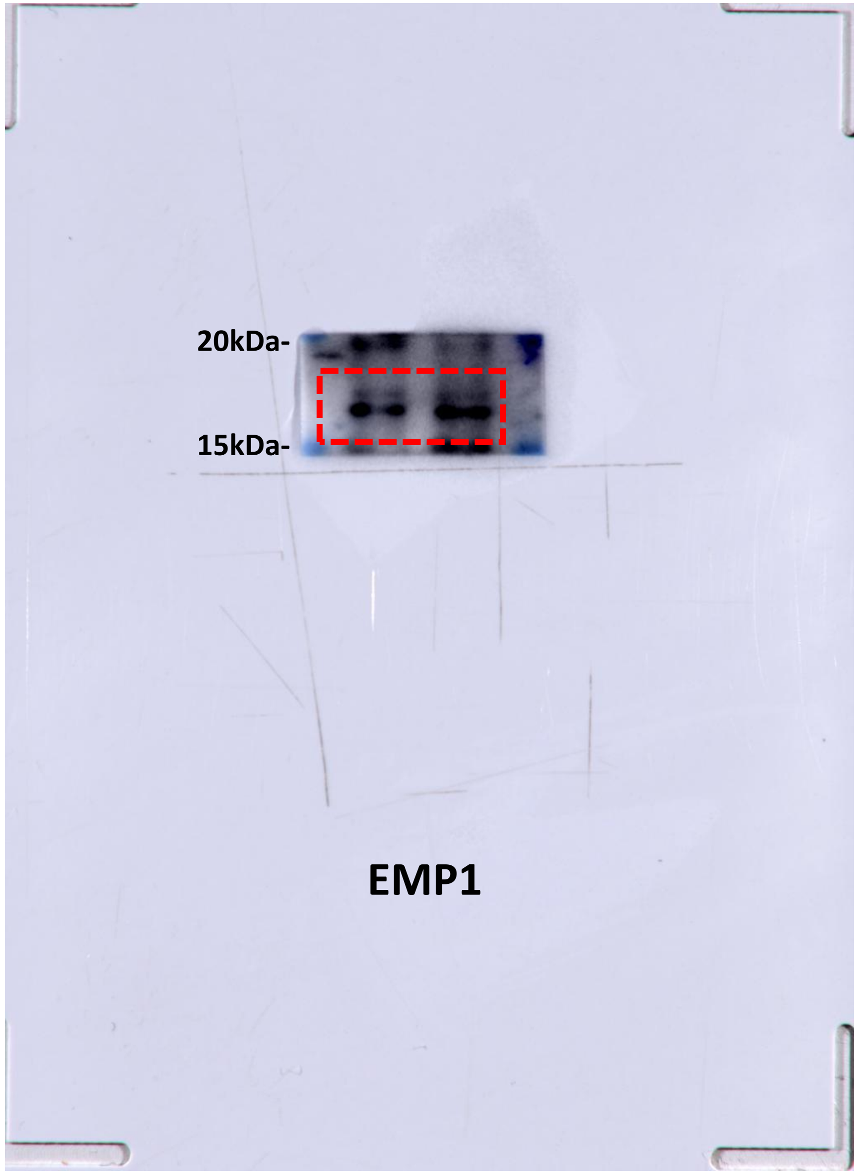

Figure 6A.（SGC-7901）

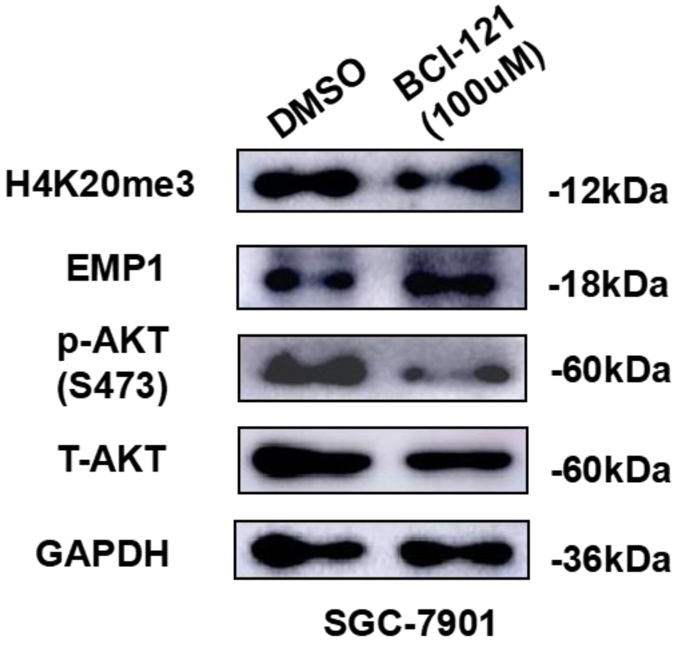

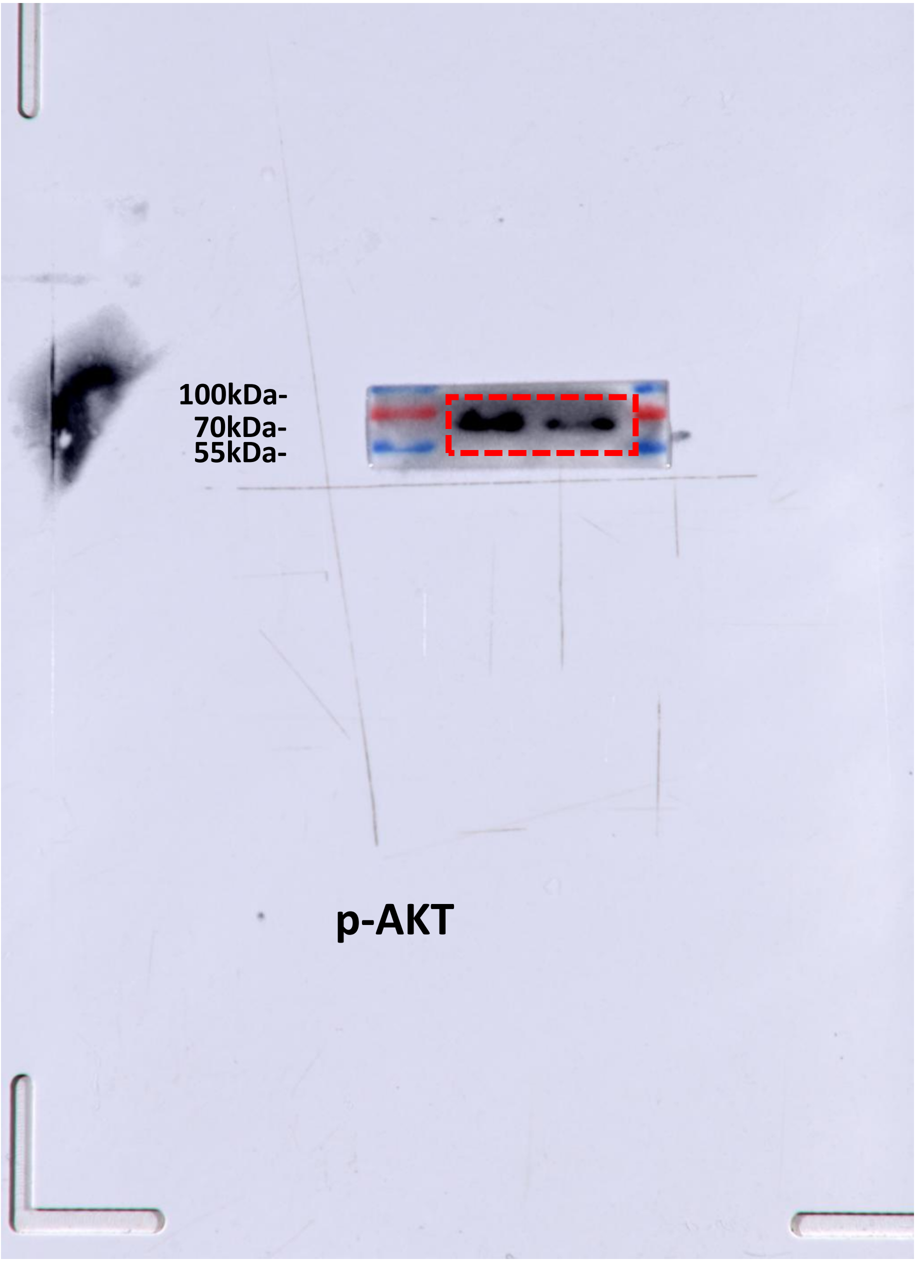

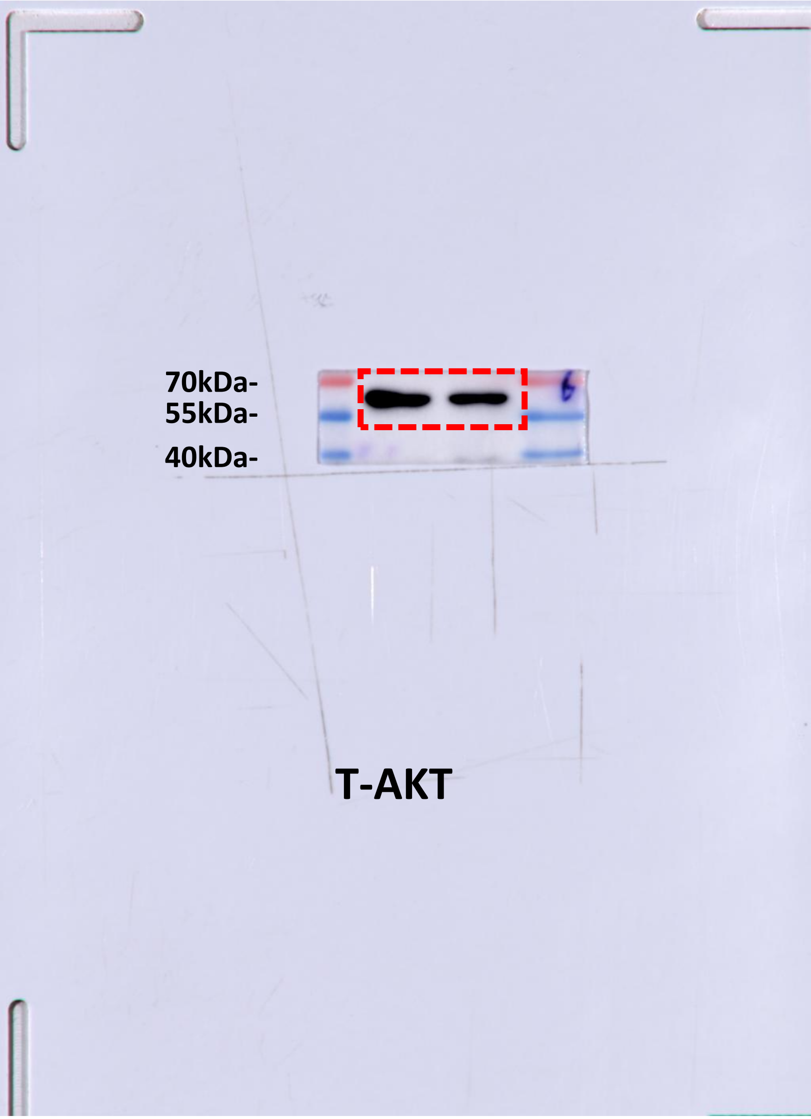

Figure 6A.（SGC-7901）

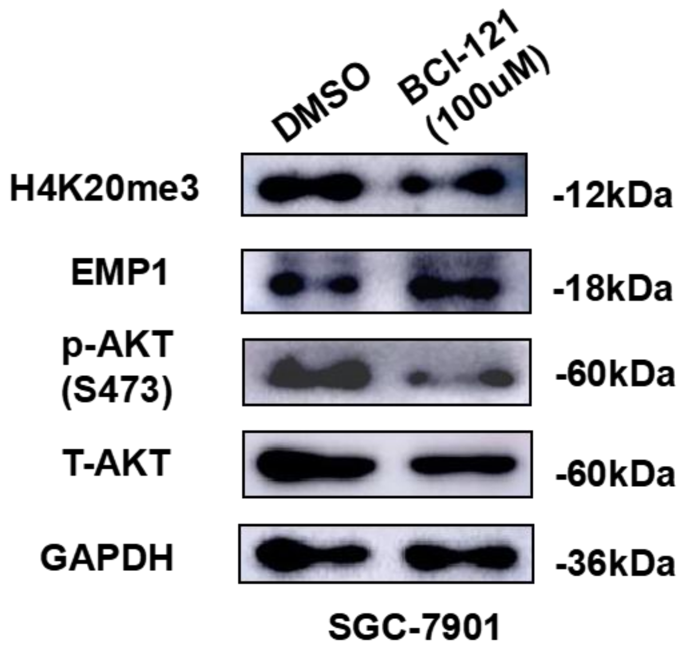

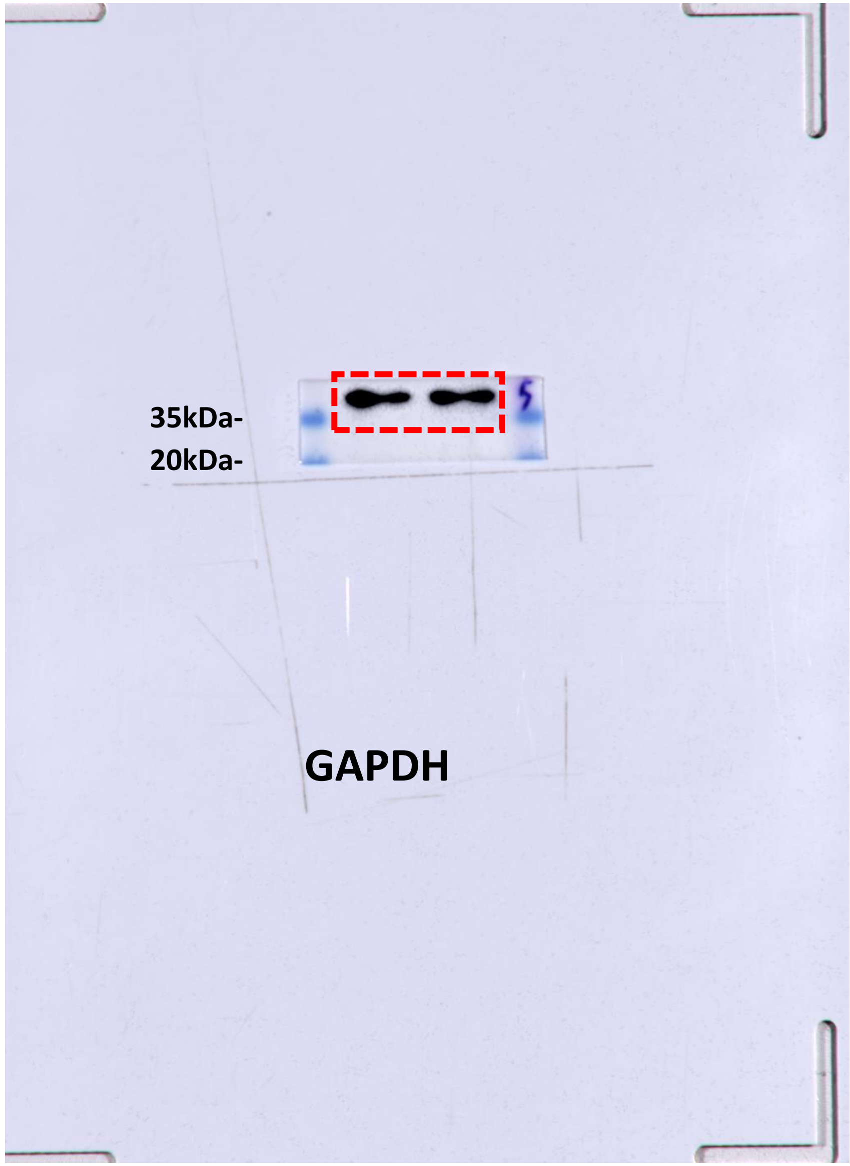

Supplement: Supplementary file 2 — Western Blot original data file [file 41419_2023_5907_MOESM2_ESM.pdf]
