# Supplementary material for: SMYD3 drives the proliferation in gastric cancer cells via reducing EMP1 expression in an H4K20me3-dependent manner
Source: Cell Death Dis. 2023 Jun 29;14(6):386. doi: 10.1038/s41419-023-05907-9 (PMC10310787; doi:10.1038/s41419-023-05907-9)
Supplement: Supplementary file 3 — Supplementary Table S1 [file 41419_2023_5907_MOESM3_ESM.pdf]

Supplementary Table S1. Real-time PCR primers in study

Gene	type	Sequence (5' to 3')
SMYD3	Forwrd primer	CACCATCTGCTACCTGGATATG
SMYD3	Reverse primer	CCGTTCAGAATTGCTGCTTATG
EMP1	Forwrd primer	TTGCTGGCTGGTATCTTT
EMP1	Reverse primer	TTGAGGGCATCTTCACTG
GAPDH	Forwrd primer	ATTCAACGGC ACAGTCAAGG
GAPDH	Reverse primer	GCAGAAGGGGCGGA GATGA
